# Supplementary material for: Identification of the missing pluripotency mediator downstream of leukaemia inhibitory factor
Source: EMBO J. 2013 Aug 13;32(19):2561–74. doi: 10.1038/emboj.2013.177 (PMC3791366; doi:10.1038/emboj.2013.177)
Supplement: Supplementary Information [file emboj2013177s1.pdf]

Figure S1

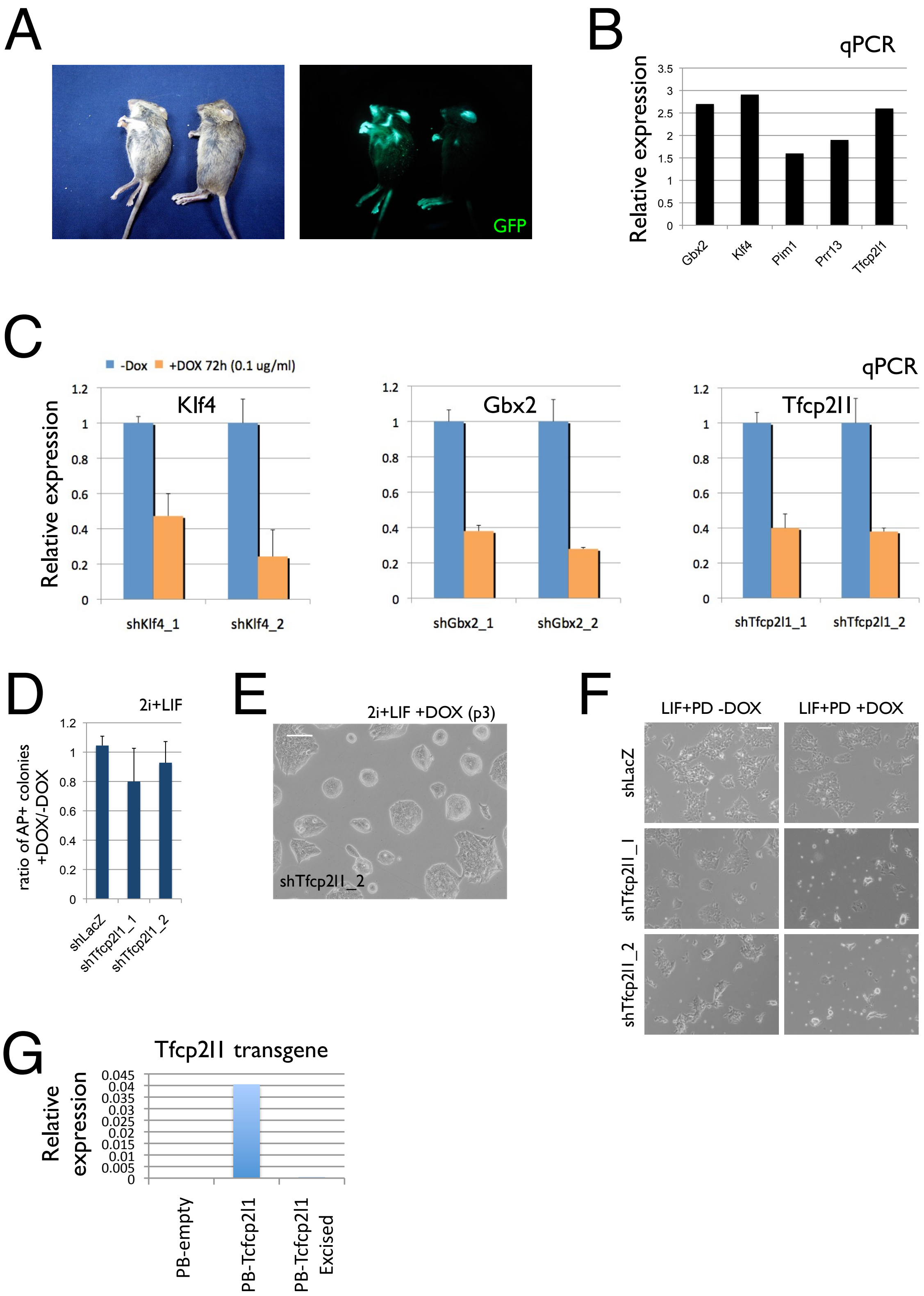
A
GFP
B
qPCR
Relative expression
3.5
3
2.5
2
1.5
1
0.5
0
Gbx2
Klf4
Pim1
Prr13
Tfcp2l1
C
-Dox
+DOX 72h (0.1 ug/ml)
qPCR
Relative expression
1.2
1
0.8
0.6
0.4
0.2
0
Klf4
shKlf4_1
shKlf4_2
Gbx2
shGbx2_1
shGbx2_2
Tfcp2l1
shTfcp2l1_1
shTfcp2l1_2
D
2i+LIF
ratio of AP+ colonies +DOX/-DOX
shLacZ
shTfcp2l1_1
shTfcp2l1_2
E
2i+LIF +DOX (p3)
shTfcp2l1_2
F
LIF+PD -DOX
LIF+PD +DOX
shLacZ
shTfcp2l1_1
shTfcp2l1_2
G
Tfcp2l1 transgene
Relative expression
0.045
0.04
0.035
0.03
0.025
0.02
0.015
0.01
0.005
0
PB-empty
PB-Tcfcp2l1
PB-Tcfcp2l1 Excised

Figure S1

A) GFP-labelled Stat3 -/- ES cells were injected into blastocyst stage embryos and gave rise to chimeric animals (4 out of 4 showed widespread GFP signal, 2 examples are shown).

B) Gene expression analysis of the indicated genes in the PB transfected cells analyzed in Figure 2A. The primers used detect the total levels (endogenous + transgenic) of the mRNA of interest, and data are normalized to PB-vector transfected cells. Note that the overexpression levels range from 1.5 to 3 fold over physiological levels. ActinB was used as endogenous control.

C) Gene expression analysis of the indicated inducible shRNA lines used in Figure 3D-G. Cells were cultured in 2i+LIF either in presence (orange bars) or absence (blue bars) of Doxycycline (0.1 μg/ml) for 72h. ActinB was used as endogenous control and mean and SD of two replicates is shown.

D) Clonogenicity assay of inducible knock-down ES cell lines in 2i+LIF. DOX-inducible shRNA constructs targeting the indicated genes were stably transfected in Rex1-GFPd2 cells. Cells were plated at clonal density in 2i+LIF media either in the presence or absence of DOX and stained for alkaline phosphatase (AP) after 5 days. Bars show the ratio between colonies obtained in presence and absence of DOX for the indicated shRNA lines. Mean and SD of 2 independent experiments is shown.

E) Inducible shTfcp2l1 cells can be cultured in the presence of DOX in 2i+LIF with no overt effects on morphology and survival. A representative picture of Rex1-GFPd2 shTfcp21_2 cells cultured for 3 passages in 2i+LIF +DOX is shown.

F) Morphology of the indicated shRNA lines cultured in N2B27 media supplemented with LIF, either in the absence or in presence of DOX for 3 days. Knockdown of Tfcp2l1 resulted in differentiation (white arrows) with minimal cell death. Similar results were obtained under LIF + Serum culture conditions. Scale bar, 100μm.

G) Gene expression analysis of parental ES cells transfected with an empty piggybac vector (PB-Vector), Tfcp2l1 expressing cells (PB-Tfcp2l1) and Tfcp2l1 expressing cells after excision of the transgene (PB-Tfcp2l1 excised). The levels of exogenous Tfcp2l1 expression were measured using primers specifically recognising the Tfcp2l1 transgene. Gapdh was used as internal control.

Figure S2

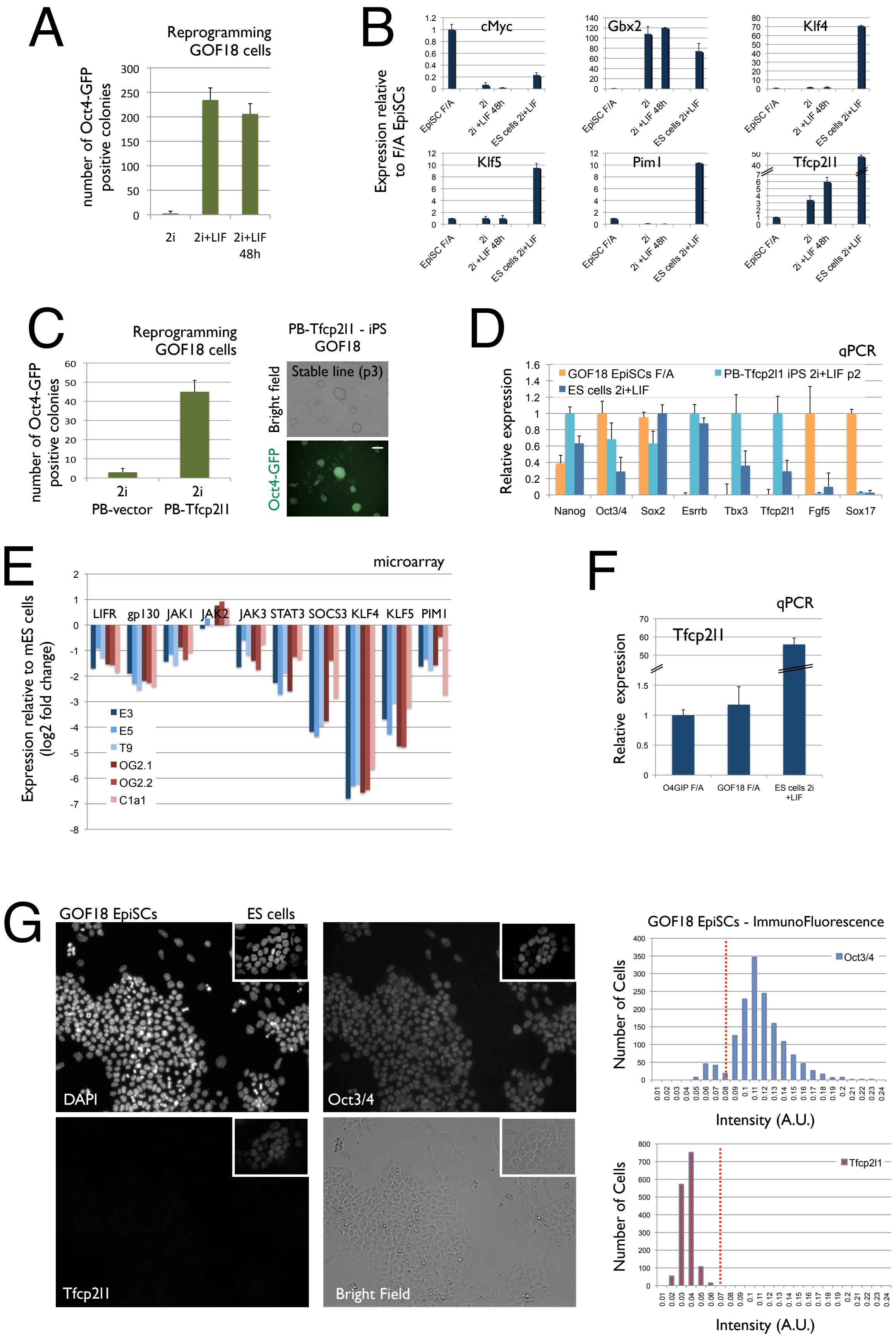
A
Reprogramming GOF18 cells
number of Oct4-GFP positive colonies
2i
2i+LIF
2i+LIF 48h
B
Expression relative to F/A EpiSCs
cMyc
Gbx2
Klf4
Klf5
Pim1
Tfcp2l1
EpiSC F/A
2i
2i +LIF 48h
ES cells 2i+LIF
C
Reprogramming GOF18 cells
number of Oct4-GFP positive colonies
2i PB-vector
2i PB-Tfcp2l1
PB-Tfcp2l1 - iPS GOF18
Stable line (p3)
Bright field
Oct4-GFP
D
qPCR
Relative expression
GOF18 EpiSCs F/A
PB-Tfcp2l1 iPS 2i+LIF p2
ES cells 2i+LIF
Nanog
Oct3/4
Sox2
Esrrb
Tbx3
Tfcp2l1
Fgf5
Sox17
E
microarray
Expression relative to mES cells (log2 fold change)
LIFR
gp130
JAK1
JAK2
JAK3
STAT3
SOCS3
KLF4
KLF5
PIM1
E3
E5
T9
OG2.1
OG2.2
C1a1
F
qPCR
Tfcp2l1
Relative expression
O4GIP F/A
GOF18 F/A
ES cells 2i +LIF
G
GOF18 EpiSCs
ES cells
DAPI
Oct3/4
Tfcp2l1
Bright Field
GOF18 EpiSCs - ImmunoFluorescence
Number of Cells
Oct3/4
Tfcp2l1
Intensity (A.U.)

Figure S2

A) GOF18 EpiSCs (Han *et al*, 2010) were cultured on fibronectin coated plates, without feeders, in bFGF/ActivinA media; after plating at low density they were exposed to the indicated culture conditions and after 6 days the number of Oct4-GFP colonies was counted. Mean and SD of 3 independent experiments is shown. Please note that exposure to LIF, either for the first 48h or for 6 days, massively increased reprogramming efficiency.

B) Gene expression analysis of GOF18 EpiSCs cultured in bFGF/ActivinA media (F/A), GOF18 cells exposed to 2i or 2i+LIF for 48h and mouse ES cells cultured in 2i+LIF. The fold change expression relative to EpiSCs cultured in bFGF and ActivinA condition (F/A) is shown and ActinB serves as an internal control. This is the same "mouse ES cell 2i+LIF" sample as used in Figure 6C to allow direct comparison between the two Figures.

C) Left: GOF18 EpiSCs were transiently transfected with PB-Tfcp2l1 and an empty piggyBac vector control (PB-Vector) in bFGF and ActivinA media; after 1 day they were replated at low density and cultured in either 2i media for 6 days; the number of Oct4-GFP positive colonies is shown as Mean and SD of 2 independent experiments. Right: Representative pictures of a stable iPS line generated after PB-Tfcp2l1 transfection in GOF18 cells. Scale bar, 100μm.

D) Gene expression analysis of parental GOF18 EpiSCs (orange) and PB-Tfcp2l1 iPS cells (light blue) and ES cells cultured in 2i+LIF (dark blue). ActinB was used as endogenous control and data are normalized to the highest value. Mean and SD of 2 independent replicates is shown.

E) Gene-expression analysis of 6 EpiSC lines and a mouse ES cell line by microarray (data from Bernemann *et al*, 2011). Three lines (E3, E5 and T9, in blue) show spontaneous conversion to naïve pluripotency as the GOF18 line, whereas the other three lines (OG2.2, OG2.1 and C1a1) do not convert

spontaneously (Bernemann et al., 2011). Expression relative to mouse ES cells levels is shown on a log2 scale. Note that all EpiSC lines show comparable levels of LIF/Stat3 pathway components and target genes.

F) Gene expression analysis of Tfcp2l1 in GOF18 and O4GIP cells under F/A culture conditions and ES cells in 2i+LIF. ActinB served as an internal control and data are Mean and SD of 2 biological replicates.

G) LEFT: Fluorescence micrographs showing immunostaining for Oct3/4 and Tfcp2l1 in GOF18 EpiSC cells. The insets on the top-right corner of each panel show immunostaining of mouse ES cells in LIF+serum and serve as positive controls. RIGHT: Distribution of Single-cells measurement of fluorescence intensity in GOF18 EpiSCs stained for Oct3/4 and Tfcp2l1. The red dashed lines separate positive from negative cells. More than 1400 cells were analyzed and we could not observe any Tfcp2l1 positive cell.

Figure S3

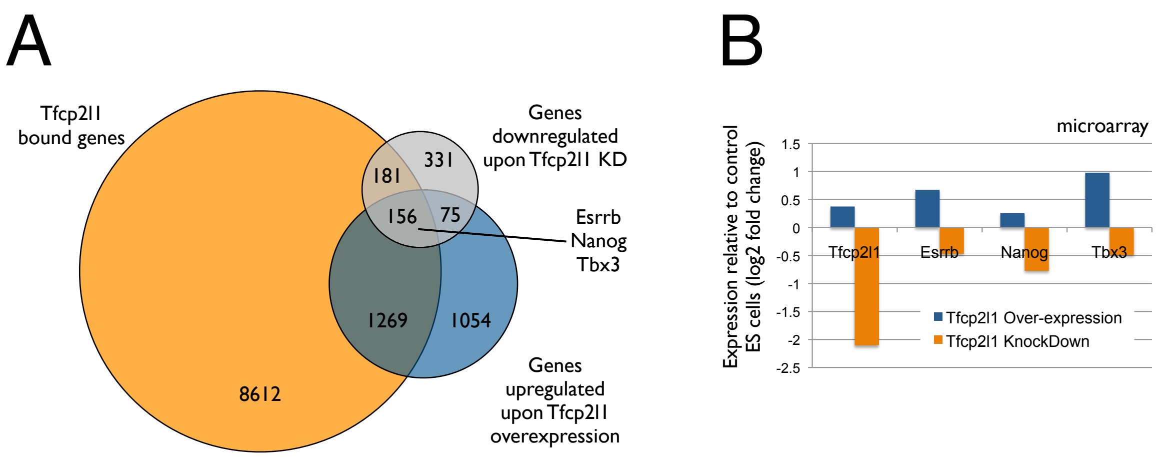

Figure S3

A) Venn diagram showing overlap between genes up-regulated upon Tfcp2l1 over-expression, downregulated upon Tfcp2l1 knockdown and genes bound by Tfcp2l1 (see Supplemental Table 5 for gene lists). Among the 156 putative Tfcp2l1 targets we found 3 genes previously involved in mouse ES cell pluripotency, namely: Esrrb, Nanog and Tbx3.

B) Gene expression analysis of Tfcp2l1 and its putative targets upon over-expression and knockdown of Tfcp2l1 itself. Microarray data (from (Nishiyama *et al*, 2013; Correa-Cerro *et al*, 2011)) are normalized to control ES cells and shown as log2 fold change.

TABLE S2 – qPCR primer sequences

Gene Name	Forward	Reverse
Actb	CTAAGGCCAACCGTGAAAAG	ACCAGAGGCATACAGGGACA
Bcor	GCTCTGTGCTTGGCGTAATTT	CCACTGGATCACGTCCAATA
cMyc	GACCAGATCCCTGAATTGGA	CGAAGCTGTTCGAGTTTGTG
Esrrb	GGCGTTCTTCAAGAGAACCA	CCCACTTTGAGGCATTTCAT
Fgf5	AAAACCTGGTGCACCCTAGA	CATCACATTCCCGAATTAAGC
Gbx2	GGCACCTCCTAGATGTGGAC	AAAACACTGCAGCTGAGATCC
Gcnt2	GCCCCATATGCATACACACATAC	TCAGTCCAATGACAGCACAAGA
Gtl2	TGGGCAGGTTTTCTGTCTTC	CGACGGAACAAGAGTCCATT
Klf4	CGGGAAGGGAGAAGACACT	GAGTTCCTCACGCCAACG
Klf5	CCGGAGACGATCTGAAACAC	CAGATACTTCTCCATTTCACATCTTG
Nanog	TTCTTGCTTACAAGGGTCTGC	AGAGGAAGGGCGAGGAGA
nMyc	AATGGTGCTTAAGTTCCAGC	CTTGCAGTCTAATACTGGCC
Pim1	GCCCTCCTTTGAAGAAATCC	GGACCTGGAGTCTGGAATGA
Pim3	AGCAGTGACCTCTGACCCCT	TCAAGTATCCACCCAGGGCA
Pou5f1	GTTGGAGAAGGTGGAACCAA	CTCCTTCTGCAGGGCTTTC
Prr13	ATCACAAGCACGGGAAGC	GAGCTGGTGAGGCTGGAG
Rex1	TCTTCTCTCAATAGAGTGAGTGTGC	GCTTTCTTCTGTGTGCAGGA
Rtn4	AACCCCTAGCAACTGTGTTT	CTGATGTCTGCACTTTCCTCA
Smad7	ATCCTGTGTGCTGAGCTCTG	TGCTCATTGAGCTAAGAACAGT
Socs2	CACTCCTAGGCGAAGAAGTTGT	GTGACTGTGCCATGAAGCAT
Socs3	ATTTCGCTTCGGGACTAGC	AACTTGCTGTGGGTGACCAT
Sox17	CACAACGCAGAGCTAAGCAA	CGCTTCTCTGCCAAGGTC
Sox2	TCCAAAAACTAATCACAACAATCG	GAAGTGCAATTGGGATGAAAA
Spry2	CACTCCTAGGCGAAGAAGTTGT	GTGACTGTGCCATGAAGCAT
Stat3	GTCCTTTTCCACCCAAGTGA	TATCTTGGCCCTTTGGAATG
Tfcp2l1	GGGGACTACTCGGAGCATCT	TTCCGATCAGCTCCCTTG
Tfcp2l1_TG	GTGCTGGTTGTTGTGCTGTC	TTGTGCTGGTTGTAGTGTTCG
Tgtp	TGAGAAGAAGGTGGGACCAT	GGAAAGCATGAATCCTCTGG
Trh	ATCCCTTCAAGCAAGGAGGT	GGAGGATGCGCTGAAGTTATAC
Vim	CCTTTACTGCAGTTTTTCAGG	GTATTCTAGCACAAGATTTC
Zfp36	ATCCTGCCTTAGCCTTTTCC	GAGGGAAATTTGAGCACCAG
Zfp36l1	AATCTTGCCTGGGTTTGTGT	CAAAACCTTCGTCCAACTTGT

TABLE S3 – siRNA used in this study.

Name	Cat. from Qiagen
siGFP	Custom (target sequence GCAAGCTGACCTGAAGTTCA)
siGbx2	GS14472
siKlf5	GS12224
siPim1	GS18712
siStat3	GS20848
siTfcp2l1	GS81879

TABLE S4 – shRNA sequences.

Name	Target sequence
shKlf4_1	TGTATACTGGGTCCAACTCCG
shKlf4_2	TATCCATTCACAAGCTGACTT
shGbx2_1	ATCAGCGAGTCTATGCTGAAG
shGbx2_2	AAACAGTGGAGTCTGACACGG
shTfcp2l1_1	ATCAAAGGACCCTCCAACTGC
shTfcp2l1_2	AATACATCAAAGGACCCTCCA
