# Supplementary material for: Identification of the missing pluripotency mediator downstream of leukaemia inhibitory factor
Source: EMBO J. 2013 Aug 13;32(19):2561–74. doi: 10.1038/emboj.2013.177 (PMC3791366; doi:10.1038/emboj.2013.177)
Supplement: Supplementary Table S1 [file emboj2013177s2.pdf]

List of genes upregulated (p-value < 0.05) after LIF treatment (1h) in Stat3 wild-type cells cultured in 2i

ENSEMBL_ID	GENE SYMBOL	Stat3_null_2i (FPKM)	Stat3_null_2i+LIF_1h	Stat3_wt_2i (FPKM)	Stat3_wt_2i+LIF_1h	Adj_pvalue	Fold Change
ENSMUSG00000000308	Ckmt1	10.87280574	11.53381236	3.975278346	9.67698789	8.88E-05	2.434291903
ENSMUSG00000000409	Lck	9.327416393	8.185729491	2.225449894	4.196919076	0.033249806	1.885874442
ENSMUSG00000002325	Irf9	0.995737078	3.752201388	0.913760545	3.390133322	9.42E-09	3.71008941
ENSMUSG00000003032	Klf4	5.604567897	6.267156598	8.808995703	16.7249052	0.004296651	1.898616569
ENSMUSG00000004040	Stat3	0.761242157	0.95023724	1.212505818	2.54080572	0.004944459	2.095499817
ENSMUSG00000005672	Kit	7.122007349	5.907731598	5.365805677	13.23797076	7.62E-06	2.467098431
ENSMUSG00000006219	Fblim1	1.663114325	1.324580365	2.260196173	5.579048025	9.52E-05	2.468391059
ENSMUSG00000006345	Ggt1	0.299940264	0.21538123	0.031174538	0.351839318	0.00626428	11.28611153
ENSMUSG00000008682	Rpl10	28.45748248	37.64902764	23.70195743	40.77779561	0.015085413	1.720439998
ENSMUSG00000010607	Pigyl	21.2825985	19.19986357	16.92906326	29.79315833	0.03194433	1.759882273
ENSMUSG00000013766	Ly6g6e	0.113805772	0.101512161	0.310528863	3.161882796	4.89E-07	10.18225091
ENSMUSG00000015243	Abca1	0.606943062	0.628804042	0.93098085	2.084421659	0.001006047	2.238952239
ENSMUSG00000017144	Rnd3	3.869265142	4.641943528	3.2974576	6.109490579	0.01523171	1.852788214
ENSMUSG00000017774	Myo1c	7.236847736	6.374918679	4.140143819	8.080001271	0.003849208	1.951623331
ENSMUSG00000018411	Mapt	0.809118953	0.715913205	0.254156727	0.716800255	0.003195931	2.820308015
ENSMUSG00000018899	Irf1	10.49490231	18.77788232	6.909445017	13.67385297	0.002348115	1.979008869
ENSMUSG00000020333	Acsl6	0.282810571	0.323450335	0.140951547	0.406803264	0.009465109	2.886121305
ENSMUSG00000020788	Atp2a3	0.701257046	0.676997001	0.261650855	0.836183671	0.001882375	3.195799496
ENSMUSG00000021127	Zfp36l1	6.047486891	7.155911897	7.768739565	16.92644168	0.00020762	2.178788661
ENSMUSG00000021678	F2rl1	14.93914631	13.83867281	10.03091364	22.5591975	0.000108313	2.248967373
ENSMUSG00000021732	Fgf10	0.123974614	0.120667386	0.264279915	0.885658379	0.007306894	3.351213354
ENSMUSG00000022101	Fgf17	0.932073528	1.617117616	2.648082703	5.514387573	0.037399519	2.08240761
ENSMUSG00000022114	Spry2	11.367465	12.86278733	9.769656079	22.96248437	2.77E-05	2.3503882
ENSMUSG00000022438	Parvb	0.393681379	0.237895484	0.32010949	0.942579628	0.013163269	2.94455384
ENSMUSG00000022750	Klhl22	16.42070233	16.45729405	11.98661591	20.29997655	0.035306178	1.693553602
ENSMUSG00000022900	Ildr1	4.630518024	5.007822437	2.418881427	4.820816793	0.005946475	1.992994258
ENSMUSG00000023048	Prr13	9.370745243	9.438909786	4.191927341	13.76315413	1.14E-07	3.283252073
ENSMUSG00000023942	Slc29a1	49.97322082	48.72308634	26.14760645	52.57783632	0.001181505	2.010808768
ENSMUSG00000023951	Vegfa	2.175045223	2.35123266	0.438579866	1.66023673	1.39E-06	3.785483235
ENSMUSG00000023987	Pgc	0.410956195	0.424130968	0.503383876	1.712521336	0.031257586	3.402018651
ENSMUSG00000023990	Tfeb	0.299738576	0.395112552	0.436931913	1.698125748	5.62E-06	3.886476808
ENSMUSG00000024014	Pim1	41.42322639	44.10523027	16.98457044	34.46519752	0.00067086	2.02920631

ENSMUSG00000024209	1700061G19Rik	4.858421669	5.577474223	5.778207807	10.82661763	0.014660988	1.87369821
ENSMUSG00000024349	Tmem173	0.658151287	0.65699785	0.296992068	1.175200191	0.007286651	3.957008684
ENSMUSG00000024617	Camk2a	0.236930229	0.256134363	0.105749189	0.343643547	0.01863589	3.249609289
ENSMUSG00000024738	Pga5	0.809940389	0.819654687	0.289777064	1.72278076	0.000891257	5.945193647
ENSMUSG00000025868	Higd2a	33.13581195	34.35583906	13.87614721	25.37159845	0.025477546	1.828432494
ENSMUSG00000026142	Rhbdd1	5.474147358	5.406554938	2.949859859	5.817153986	0.007306894	1.972010287
ENSMUSG00000026235	Epha4	13.23649528	12.57640133	8.504565831	14.7323933	0.023954413	1.732292229
ENSMUSG00000026380	Tfcp2l1	81.30807865	70.11931171	39.48077653	88.74400635	0.003082944	2.247777632
ENSMUSG00000026576	Atp1b1	43.69703345	38.86786336	27.56769709	48.32776284	0.016125918	1.753057671
ENSMUSG00000026656	Fcgr2b	0.052537939	0.034195576	0.039489214	0.508826717	0.000197505	12.88520741
ENSMUSG00000026959	Grin1	0.046981406	0.033305407	0.053202709	0.22739473	0.007766283	4.274119375
ENSMUSG00000027313	Chac1	1.290548596	1.353668391	0.417639426	2.621215689	4.44E-07	6.276264949
ENSMUSG00000027463	Slc52a3	0.210527498	0.208429647	0.18650751	0.797475433	0.007951845	4.275835511
ENSMUSG00000028159	Dapp1	0.442039978	0.478755773	1.230987975	2.704509543	0.031174023	2.197023526
ENSMUSG00000028194	Ddah1	1.335128926	1.209442958	5.307061102	9.490586128	0.03296606	1.788294113
ENSMUSG00000028199	Cryz	13.0189953	12.05479534	4.315898279	9.843694403	0.000268685	2.280798519
ENSMUSG00000028435	Aqp3	21.87444967	20.09298248	1.917939195	15.33546684	1.40E-21	7.995804497
ENSMUSG00000028451	1700022I11Rik	0.930545283	0.549241415	0.077930612	0.386260147	0.007232926	4.956462362
ENSMUSG00000028640	Tfap2c	5.963083778	5.339652338	1.193536649	2.788579358	0.002383024	2.336400279
ENSMUSG00000028709	Mob3c	1.295434639	1.090748686	0.577141878	1.649281185	0.00642679	2.857670268
ENSMUSG00000028927	Padi2	0.449144302	0.425814078	0.036312816	0.317008139	0.001006047	8.729924413
ENSMUSG00000028949	Smarcd3	0.369197758	0.489667818	1.54111482	2.953819166	0.044836382	1.916676893
ENSMUSG00000029718	Pcolce	1.930233523	2.223414422	4.833955008	12.78599403	4.45E-06	2.645037864
ENSMUSG00000030208	Emp1	0.561793038	0.637939032	0.65816651	1.827873333	0.006284103	2.777220209
ENSMUSG00000030523	Trpm1	5.377201491	5.383334219	8.645222576	16.31664919	0.006055377	1.887360221
ENSMUSG00000030717	Nupr1	3.44143141	3.276290034	1.718006341	5.630577177	4.34E-06	3.277390219
ENSMUSG00000030825	Hsd17b14	11.14448195	12.37464858	7.699025709	14.8121529	0.005895866	1.923899654
ENSMUSG00000031216	Stard8	0.731043277	0.642759893	0.608086505	1.507239583	0.007286651	2.478659814
ENSMUSG00000031381	Piga	6.063592573	6.069119017	5.809535855	9.69719274	0.048418844	1.669185453
ENSMUSG00000031548	Sfrp1	18.01296416	14.89033981	7.177067563	12.42569331	0.030200412	1.731305049
ENSMUSG00000031758	Cdyl2	4.349665397	4.127698751	2.425217629	4.121624549	0.040504802	1.699486471
ENSMUSG00000031770	Herpud1	2.111231824	2.228932132	1.419394751	3.294054	0.006967793	2.320745513
ENSMUSG00000032092	Mpzl2	23.36812762	21.1361825	25.76731953	44.2714196	0.026214575	1.718122816
ENSMUSG00000032291	Crabp1	11.16200544	9.10371778	5.947032806	11.74533287	0.034951791	1.974990429
ENSMUSG00000032434	Cmtm6	47.77014372	51.57852448	19.40624968	33.09657652	0.020719819	1.705459688

ENSMUSG00000032470	Mras	1.428692377	1.283790312	1.61132279	5.599098389	3.58E-08	3.474845898
ENSMUSG00000032796	Lama1	0.585370581	0.465386245	0.873566504	2.140128214	0.00044267	2.449874399
ENSMUSG00000033857	Engase	0.738209923	0.780603587	0.292968957	0.902429917	0.002456958	3.080291944
ENSMUSG00000034165	Ccnd3	11.106899	9.274332929	8.448586571	15.79937153	0.010595574	1.870060915
ENSMUSG00000034486	Gbx2	0.144508673	0.22801735	1.100368287	2.854976548	0.001507864	2.59456455
ENSMUSG00000034714	Ttyh2	2.482075879	1.949516573	0.532162105	1.272399515	0.020403861	2.390999854
ENSMUSG00000034799	Unc13a	0.324210072	0.340862328	0.110702182	0.300703187	0.018679628	2.716325748
ENSMUSG00000034993	Vat1	29.80269972	30.10864217	13.65587589	34.99966843	1.90E-06	2.562974993
ENSMUSG00000035212	Leprot	82.08131332	93.70356761	32.08864763	74.43773856	2.98E-05	2.319753061
ENSMUSG00000035722	Abca7	0.883723704	0.808081273	0.294867135	0.689021262	0.048069456	2.336717725
ENSMUSG00000035781	C030046I01Rik	13.53592687	14.45798385	4.403650653	9.029827547	0.003447455	2.05053222
ENSMUSG00000036106	Prr5	9.838620857	6.885768916	3.478020217	6.382225472	0.038087265	1.835016784
ENSMUSG00000036327	Qsox2	13.83873925	10.41727823	4.223369802	7.249706745	0.034951791	1.716569253
ENSMUSG00000037188	Grhl3	2.311405708	2.237748254	0.762114818	2.376729999	0.000161635	3.118598331
ENSMUSG00000037405	Icam1	2.002131022	2.050106344	1.830687426	6.06385062	2.15E-06	3.31233532
ENSMUSG00000037824	Tspan14	11.80121622	12.1199606	8.519046309	15.58903615	0.010564476	1.82990391
ENSMUSG00000040010	Slc7a5	30.57988173	26.10964037	11.91466385	19.99953722	0.04044095	1.678564957
ENSMUSG00000040483	Xaf1	0.327609226	0.522092827	0.398590547	2.092766361	9.29E-09	5.250416447
ENSMUSG00000040618	Pck2	9.435699732	8.526277958	3.682535059	6.590778441	0.030073384	1.789739496
ENSMUSG00000040726	Hesx1	0.449661191	0.285237723	0.040015456	0.714736393	0.033249806	17.86150819
ENSMUSG00000040746	Rnf167	34.76791399	37.25891598	19.19663154	34.34984862	0.011125957	1.789368543
ENSMUSG00000040857	Erf	15.72542684	17.12223625	6.494050272	11.04235276	0.032099038	1.700379931
ENSMUSG00000040964	Arhgef10l	0.380100235	0.576720193	0.437832863	1.463387099	0.000137069	3.342341843
ENSMUSG00000041323	Ak7	0.508376347	0.45648226	0.265132315	0.881892471	0.014660988	3.326235318
ENSMUSG00000041548	Hspb8	11.02554753	11.9382957	9.064193243	20.93097647	0.000157915	2.309193539
ENSMUSG00000042312	S100a13	3.178373429	3.621380693	6.759679714	20.03034784	5.53E-05	2.963209603
ENSMUSG00000042357	Gjb5	0.415619957	0.657708005	0.423784646	3.056352262	2.05E-07	7.212041055
ENSMUSG00000042367	Gjb3	35.6596344	34.7673829	9.075075023	34.76928803	6.85E-12	3.831294831
ENSMUSG00000042808	Gpx2	1.31244302	1.762833731	9.858309259	21.72698852	0.003470372	2.203926449
ENSMUSG00000042842	Serpinb6b	0.076818519	0	0.276352702	1.225258547	0.006239573	4.433676744
ENSMUSG00000044041	Krt13	0.125961075	0.17478027	0.125037772	0.803094255	0.011714083	6.422813243
ENSMUSG00000044080	S100a1	12.580423	16.32031191	11.205167	25.01952898	0.000837755	2.232856411
ENSMUSG00000044734	Serpinb1a	3.459322375	3.992947167	4.043970788	15.00670882	4.12E-09	3.710884577
ENSMUSG00000045005	Fzd5	5.155066558	4.912478397	3.498843932	10.16219096	2.80E-08	2.904442484
ENSMUSG00000045827	Serpinb9	0.400794706	0.50888391	0.60884986	1.484187007	0.038936514	2.43768966

ENSMUSG00000045999	AY036118	127.5681638	164.0308142	49.91508554	92.44246046	0.00059009	1.851994432
ENSMUSG00000046623	Gjb4	0.039656832	0	0	0.561529506	0.007148629	Inf
ENSMUSG00000046711	Hmga1	22.48260764	16.32439266	17.27658847	62.04972381	2.82E-12	3.591549565
ENSMUSG00000046805	Mpeg1	0.051514169	0.06734789	0	0.159094049	0.018945722	Inf
ENSMUSG00000046879	Irgm1	2.879222096	5.472718914	2.401311767	4.3327115	0.034056781	1.804310277
ENSMUSG00000047904	Sstr2	0.271602011	0.199325277	0.138043321	0.841857948	0.005045113	6.098505466
ENSMUSG00000048232	Fbxo10	2.22232373	2.268065864	0.903397688	1.837978157	0.025858064	2.034517223
ENSMUSG00000049742	C330006D17Rik	6.58705956	12.96564493	2.811558502	8.126894584	0.020996594	2.890530138
ENSMUSG00000051853	Arf3	60.79824712	46.64361557	18.97810844	41.82796662	8.58E-05	2.204011361
ENSMUSG00000052180	Serpinb6c	0.156184565	0.13590934	5.494166041	11.96179533	0.006457101	2.177181257
ENSMUSG00000053113	Socs3	0.120059936	0.078637361	0.110345573	5.837248174	3.27E-29	52.8997041
ENSMUSG00000053175	Bcl3	0.059519253	0.052002004	0	0.886904469	8.43E-08	Inf
ENSMUSG00000053654	Krt42	1.190830185	1.634701956	0.301743848	1.196783634	0.024765926	3.966223811
ENSMUSG00000053664	Uck2	537.3005673	446.2655217	125.786961	261.695194	0.001619796	2.080463602
ENSMUSG00000055629	B4galnt4	9.929559864	9.165263094	5.302652404	10.95394253	0.000862319	2.065747799
ENSMUSG00000055675	Kbtbd11	0.048928562	0.064890593	0.159113507	0.540614048	0.006365608	3.397662829
ENSMUSG00000057963	Itpk1	19.11138771	16.49288921	8.617331237	14.51309243	0.043097228	1.684174836
ENSMUSG00000058625	Gm17383	2.244054522	2.650525155	1.430646786	6.961612228	1.35E-06	4.86605939
ENSMUSG00000058672	Tubb2a	30.15455359	33.63116899	10.61926746	19.67826541	0.013794549	1.853071833
ENSMUSG00000059776	Gm10071	2.201850162	3.691185163	2.984966729	8.247752242	0.00817272	2.763096876
ENSMUSG00000059866	Tnip2	5.842357954	5.308093533	2.754485261	5.252572788	0.021336387	1.90691628
ENSMUSG00000062683	Atp5g2	6.131815525	7.575326573	4.580036895	12.8944639	0.003254398	2.815362451
ENSMUSG00000063556	Gm10132	0.99080685	1.28053219	1.21066054	4.691589766	0.014127296	3.875231422
ENSMUSG00000063586	Gm5513	0.926676526	1.760411439	0.973165298	2.9126731	0.023115879	2.992989071
ENSMUSG00000064345	mt-Nd2	409.1234549	231.2582577	522.212792	952.5193324	0.029300417	1.824006127
ENSMUSG00000064350	mt-Ty	101.9363193	30.43081038	45.0672999	276.9400227	3.23E-14	6.145032503
ENSMUSG00000064363	mt-Nd4	307.8405759	118.451784	272.2964826	616.6803702	0.000412206	2.264738657
ENSMUSG00000064367	mt-Nd5	1386.856568	608.7192209	929.1417269	3233.433233	0.002187901	3.480021551
ENSMUSG00000064369	mt-Te	58.91979616	32.08074325	24.40555009	131.6151032	2.13E-09	5.392834937
ENSMUSG00000064370	mt-Cytb	976.1721334	484.8314253	858.8159091	2057.295346	0.006012731	2.395502137
ENSMUSG00000064371	mt-Tt	176.1626166	245.8768894	184.0805185	340.3588799	0.003397389	1.848967412
ENSMUSG00000064694		179.6042413	349.5953131	347.3849655	623.0750731	0.007043902	1.793615542
ENSMUSG00000064871	Snord58b	75.49769128	94.27166001	26.89845798	91.64675296	5.23E-05	3.407137801
ENSMUSG00000065327		286.8281645	574.7200186	568.5879962	943.0537881	0.040310633	1.658588986
ENSMUSG00000065883		33.44991262	79.38662874	193.1985982	321.0491672	0.014447643	1.661757229

ENSMUSG00000066705	Fxyd6	40.6635638	43.88731567	22.36736509	47.94166817	0.000182424	2.143375761
ENSMUSG00000068480	Gm7551	2.256146135	0.767467404	0.210877609	1.396978536	0.03194433	6.624593965
ENSMUSG00000069874	Irgm2	0.109181238	0.350268386	0.117466281	0.839014157	1.33E-05	7.142595736
ENSMUSG00000071532	Gm10335	2.72230019	2.956120529	2.652793712	7.794113358	0.021631023	2.93807744
ENSMUSG00000072568	Fam84b	0.175237148	0.262406167	0.153026281	0.649906097	0.000944801	4.247022756
ENSMUSG00000075014	Gm10800	6.502203195	4.847780313	18.88524617	35.20349308	0.010173987	1.864073826
ENSMUSG00000076030		1.377127673	1.556643991	3.38884673	15.80083389	0.023101303	4.662599152
ENSMUSG00000077506	Scarna9	2979.741589	2645.341085	1078.927175	1685.113514	0.030183369	1.561841757
ENSMUSG00000077564		1190.245166	1442.857759	764.2459731	2147.231641	1.13E-08	2.809608053
ENSMUSG00000077886	Mir295	55.85216332	76.68178033	65.39679365	146.8985341	0.009652976	2.246265083
ENSMUSG00000078249	Hmga1-rs1	16.85133059	12.67312976	3.524966219	7.25078505	0.010605207	2.05698001
ENSMUSG00000078532	Nkain1	9.458366115	11.28537434	5.746373382	12.28492304	0.001187827	2.137856735
ENSMUSG00000078706	Gm53	5.791099211	4.890712761	3.25866041	6.234826844	0.006094876	1.913309784
ENSMUSG00000078922	Tgtp1	0	0.039572914	0	0.263921234	0.00505899	Inf
ENSMUSG00000079014	Serpina3i	0.024421395	0.230266341	0.109401644	2.123525295	4.96E-10	19.41036
ENSMUSG00000080775	Gm6368	9.700938219	8.94248004	5.653727926	10.16733884	0.029484886	1.798342434
ENSMUSG00000080851	Gm12094	0.773639988	1.226946237	0.805682546	3.597319046	0.048144237	4.464933568
ENSMUSG00000081043	Gm11512	4.006029322	2.284770497	2.222756909	9.279252858	2.91E-05	4.174659326
ENSMUSG00000081159	Gm6023	32.97412003	25.93266981	29.27716289	54.01373096	0.009938844	1.844910013
ENSMUSG00000081783	Gm15599	0.630886999	1.417306241	0.137208195	1.556546312	0.006457101	11.34441217
ENSMUSG00000082179	Gm11407	1.363597618	0.529270609	0.731382063	5.675620214	7.03E-09	7.760130448
ENSMUSG00000082390	Gm12136	0.821264725	1.522141439	0.88003135	2.751287113	0.022330944	3.126351254
ENSMUSG00000082600	Gm9673	0.336226509	0.588308326	0.136154098	1.247587707	0.033732648	9.163056584
ENSMUSG00000082896	Gm5844	5.139368965	7.476276916	6.213069868	15.40183182	2.86E-05	2.478940709
ENSMUSG00000083076	Gm11408	1.523717356	0.359645668	0.789460364	3.381693052	0.019258539	4.28355014
ENSMUSG00000083219	Gm11410	0.31427442	0.136086397	0.292239836	1.340330444	0.035306178	4.586405679
ENSMUSG00000083320	Gm13935	3.766771613	8.678615511	9.421867113	17.95364981	0.029072349	1.905529933
ENSMUSG00000083431	Gm13437	0.792154802	0.910833999	1.296757537	3.696964415	0.003220505	2.850929576
ENSMUSG00000083563	Gm13340	7.653683476	5.885841071	5.802762198	21.76508711	6.68E-10	3.750814935
ENSMUSG00000083863	Gm13341	4.805530437	3.553985526	8.819310512	18.02839908	0.029765737	2.044195979
ENSMUSG00000083982	Gm14650	0.380204143	0.519530475	0.495350375	2.012367361	0.034314218	4.062513045
ENSMUSG00000086219	2410137F16Rik	9.122393523	8.49650059	2.643714262	4.840218522	0.02689063	1.830840266
ENSMUSG00000086967	4930566F21Rik	28.69308696	33.66613906	14.82493081	25.12311541	0.030726498	1.694653131
ENSMUSG00000087943		102.0897524	235.191834	170.4513548	317.0848615	0.009476443	1.860266009
ENSMUSG00000088144		6.384354898	5.320091852	7.651208333	25.79470661	0.00642679	3.371324566

ENSMUSG00000088609		68.09528053	152.2739969	59.40278882	156.8516618	0.00039674	2.640476397
ENSMUSG00000088835		761.8268774	775.5749357	163.6789942	660.1411493	5.14E-14	4.033145197
ENSMUSG00000088948		58.93301252	105.4229955	125.294102	206.5934664	0.021261178	1.648868247
ENSMUSG00000089993	Gm5822	4.936471712	9.030419907	5.954066235	11.83187707	0.014127296	1.987192718
ENSMUSG00000090266	1110005A03Rik	10.49891951	8.444418564	6.500211787	11.231462	0.036067851	1.727860933
ENSMUSG00000090862	Rps13	21.7584336	28.9962867	36.15864523	64.43445515	0.025094762	1.781993068
ENSMUSG00000091021	Gm17300	8.868360311	8.943767976	3.10066994	6.128464087	0.010403992	1.976496759
ENSMUSG00000091732	Gm17541	3.788735745	3.18619676	1.013859329	5.192232636	0.001533708	5.121255471
ENSMUSG00000092019	Gm4027	8.189620688	8.45482412	7.140004298	15.7720395	0.003447455	2.208967788
ENSMUSG00000092267	Gm20417	7.463744604	6.244446512	1.96348456	9.279432569	3.22E-05	4.726002312
ENSMUSG00000092274	Neat1	0.923092842	0.787815063	0.83661326	1.461929009	0.023890687	1.747437052
ENSMUSG00000092509	Gm20394	0.544653311	0.561431924	0	0.693333218	0.024859074	Inf
ENSMUSG00000092702		24.01910391	74.99113662	50.84026622	91.81898614	0.017964896	1.806028823
ENSMUSG00000092998	Mir5099	102.24795	119.0382822	17.02468028	95.09759364	1.31E-08	5.585866639
ENSMUSG00000096449		3.714320738	1.771589354	2.353040128	16.2214583	4.60E-10	6.893829864
ENSMUSG00000096887		64.40309421	150.2692786	264.8496848	594.4436503	4.57E-05	2.244456703

List of genes downregulated (p-value < 0.05) after LIF treatment (1h) in Stat3 wild-type cells cultured in 2i

ENSEMBL_ID	GENE SYMBOL	Stat3_null_2i (FPKM)	Stat3_null_2i+LIF_1h	Stat3_wt_2i (FPKM)	Stat3_wt_2i+LIF_1h	Adj_pvalue	Fold Change
ENSMUSG00000000581	C1d	7.225479407	10.09998057	32.2041376	19.29897025	0.024448347	0.599269898
ENSMUSG00000000730	Dnmt3l	59.22538792	57.49975589	42.62447578	24.03194133	0.007298174	0.563806144
ENSMUSG00000000731	Aire	1.835280605	1.657303423	2.569569126	1.2780803	0.025094762	0.4973909
ENSMUSG00000000740	Rpl13	85.76464161	80.69665781	339.691723	132.8148784	2.09E-07	0.390986501
ENSMUSG00000001289	Pfdn5	78.94110527	69.80522024	181.0504781	87.63501445	3.22E-05	0.48403636
ENSMUSG00000001305	Rrp15	26.26051893	24.55927794	25.46363078	14.99695835	0.020754082	0.588956009
ENSMUSG00000001604	Tcea3	8.448042052	7.102230225	12.68398299	5.568898349	0.000318749	0.439049655
ENSMUSG00000003429	Rps11	277.7160315	226.8947608	734.8107901	398.205513	0.002639846	0.541915713
ENSMUSG00000003955	Fam162a	54.27593182	63.96431244	201.8372352	100.5716543	0.000119078	0.498280975
ENSMUSG00000004771	Rab11a	14.62104731	15.52844872	43.29608428	24.68102349	0.005771204	0.570052094
ENSMUSG00000005947	Itgae	0.256918576	0.382910369	1.608218809	0.747726887	0.032326086	0.464941016
ENSMUSG00000006717	Acot13	33.30260464	31.43156473	68.38883788	38.74006064	0.003478371	0.566467597
ENSMUSG00000008475	Arpc5	40.76720492	50.94066389	64.19621206	37.913573	0.01485906	0.590588943

ENSMUSG00000008683	Rps15a	53.73819492	63.0726902	428.4205981	165.4364361	7.87E-06	0.386154253
ENSMUSG00000009013	Dynll1	39.40542578	43.46343767	80.99557033	48.1834757	0.018604734	0.594890258
ENSMUSG00000009734	Pou6f2	0.026032154	0	0.280400719	0	1.16E-07	0
ENSMUSG00000009927	Rps25	435.5536401	459.9467551	1594.428917	911.1590805	0.029765737	0.571464222
ENSMUSG00000010406	Mrpl52	54.38384958	39.15431672	110.8791677	60.69532249	0.002336916	0.547400596
ENSMUSG00000014226	Cacybp	175.3812312	207.6757019	645.1611106	305.1772677	0.000535693	0.473024897
ENSMUSG00000014294	Ndufa2	86.0506804	95.98967728	263.7659169	124.4469014	1.17E-05	0.47180812
ENSMUSG00000014846	Tppp3	1.084416497	1.209478027	1.093606633	0.333640196	0.02792998	0.305082455
ENSMUSG00000014980	Tsen15	3.629529018	4.326987496	10.06186951	5.057649298	0.004176983	0.502655028
ENSMUSG00000015672	Mrpl32	23.67558786	26.37663061	80.60370254	45.63692354	0.005207427	0.566188923
ENSMUSG00000015980	Lrrc27	1.160075587	1.027756196	1.734736432	0.72275575	0.004253858	0.416637212
ENSMUSG00000016252	Atp5e	37.93713333	46.29112257	166.7081093	88.13571066	0.000987086	0.528682804
ENSMUSG00000016503	Gtf3a	8.128836946	7.720126721	14.36230726	8.198213483	0.011982948	0.570814517
ENSMUSG00000018196	Glrx2	5.146510019	4.912856711	9.216718685	5.422949432	0.014127296	0.588381789
ENSMUSG00000018923	Med11	7.799206193	7.755686722	21.55930543	11.98070601	0.017480394	0.555709276
ENSMUSG00000020059	Sycp3	7.406137319	8.161791098	20.28927847	11.37969546	0.019992261	0.560872358
ENSMUSG00000020180	Snrpd3	138.6286798	126.2217569	236.4558611	147.3828264	0.034951791	0.623299527
ENSMUSG00000020267	Hint1	250.7464933	295.2842959	727.8891278	394.0764915	0.003311349	0.541396315
ENSMUSG00000020386	Sar1b	33.91634353	44.25728535	88.78166638	52.40921855	0.014546311	0.590315779
ENSMUSG00000020462	Ccdc104	16.44271128	19.14011254	33.40459413	19.85834899	0.015551614	0.594479577
ENSMUSG00000020477	Mrps24	26.75306672	31.23209302	57.55444508	32.9762556	0.006457101	0.572957581
ENSMUSG00000020660	Pomc	0.428593491	0.679658018	1.055971706	0.163024441	0.021336387	0.154383342
ENSMUSG00000020857	Nme2	23.34170478	30.76448572	68.07206503	27.41299515	1.14E-07	0.402705502
ENSMUSG00000021024	Psma6	210.667473	221.140142	354.3131218	214.0828374	0.034683025	0.604219331
ENSMUSG00000021290	2010107E04Rik	57.41427597	67.9169097	220.669398	136.1718624	0.030626652	0.617085394
ENSMUSG00000021414	1700026J04Rik	0.036547377	0.053956151	0.35494631	0.027565971	0.018602005	0.077662369
ENSMUSG00000021537	Cetn3	74.36088048	86.03515891	154.0827054	85.8797637	0.004841643	0.557361474
ENSMUSG00000021967	Mrp63	3.493908377	3.343670995	6.14278006	3.489871178	0.038447657	0.568125693
ENSMUSG00000022013	Dnajc15	22.3319968	26.38274374	108.4237	61.51215762	0.005768644	0.56733129
ENSMUSG00000022205	Sub1	63.1244744	74.64259662	150.7407107	68.24706008	4.45E-05	0.452744715
ENSMUSG00000022677	0610037P05Rik	20.54272072	24.70806668	66.2625673	38.95539367	0.014546311	0.587894422
ENSMUSG00000022773	Ypel1	0.812622625	0.749668121	1.76368093	0.781938298	0.007343331	0.443355873
ENSMUSG00000023089	Ndufa5	4.249421141	3.475959411	17.46648917	9.263341785	0.011356424	0.530349385
ENSMUSG00000023919	Cenpq	15.1009211	16.47668044	28.99144832	15.91667707	0.002740385	0.549012829
ENSMUSG00000024067	Dpy30	172.6080036	184.0462045	447.8470888	264.7526457	0.021147014	0.591167504

ENSMUSG00000024346	Pfdn1	62.61554287	72.15360043	128.7175312	75.74221809	0.008951664	0.588437468
ENSMUSG00000024538	Ppic	68.92985805	68.98533034	165.6112322	89.11503121	0.001505877	0.538097749
ENSMUSG00000024608	Rps14	830.7548718	726.21578	691.8202744	382.3665515	0.034056781	0.552696366
ENSMUSG00000024646	Cyb5	17.80384496	19.58219107	53.19581086	33.31900241	0.034056781	0.626346358
ENSMUSG00000024661	Fth1	155.3421566	135.8318378	213.382244	127.1477596	0.017978292	0.595868509
ENSMUSG00000025260	Hsd17b10	18.55005382	18.38854447	40.88460411	24.62383111	0.024972824	0.602276374
ENSMUSG00000025290	Rps24	1242.786736	1175.877869	5792.031351	2597.154922	0.004393128	0.448401392
ENSMUSG00000025362	Rps26	860.8301761	891.7021462	3470.541591	1947.258625	0.014898611	0.56108206
ENSMUSG00000025794	Rpl14	24.18426488	19.63335632	109.7165449	35.29517867	3.76E-12	0.321694223
ENSMUSG00000025894	Aasdhppt	5.190558865	6.602179753	16.31352602	9.199219435	0.012866132	0.563901355
ENSMUSG00000025978	Rftn2	0.563922028	0.709642094	1.208801042	0.596982379	0.041096332	0.493863224
ENSMUSG00000026154	1110058L19Rik	9.271374561	16.24486407	26.36381463	14.36683798	0.020996594	0.544945342
ENSMUSG00000026429	Ube2t	6.100179704	6.536640998	22.0126307	12.34355209	0.014589488	0.560748611
ENSMUSG00000026688	Mgst3	7.343013423	10.54515906	23.49077756	11.93042744	0.002822955	0.507877077
ENSMUSG00000027108	Ola1	19.56932378	19.95624657	45.97296636	28.9766973	0.039169447	0.630298621
ENSMUSG00000027166	Dnajc24	15.9761821	21.16520874	28.41571317	16.35468598	0.026214575	0.575550783
ENSMUSG00000027239	Mdk	41.19878157	41.39925125	24.97408513	11.97025993	0.000200191	0.479307245
ENSMUSG00000027679	Dnajc19	10.08163538	11.8353721	27.07701874	17.25086584	0.04011114	0.63710359
ENSMUSG00000027835	Pdcd10	10.19540492	14.49681234	50.88918731	27.84622688	0.002435556	0.547193389
ENSMUSG00000028145	Them4	1.035476656	0.950680684	2.253666969	1.092433777	0.018756316	0.484736117
ENSMUSG00000028261	Ndufaf4	3.917617724	4.412011215	11.01184193	6.351845609	0.0173346	0.576819541
ENSMUSG00000028691	Prdx1	201.8029408	252.447072	556.9097162	314.4215889	0.044709859	0.564582696
ENSMUSG00000028790	Khdrbs1	4.881234116	6.421263499	12.44233589	7.652053105	0.032574588	0.615001329
ENSMUSG00000028832	Stmn1	0.902886363	1.528965358	3.550218262	1.743215387	0.033637424	0.491016399
ENSMUSG00000028936	Rpl22	205.3043173	256.0115128	631.1827337	342.6542669	0.022366456	0.54287649
ENSMUSG00000029142	Mrpl33	5.481968834	6.549917346	12.84757762	7.133803209	0.003170688	0.555264457
ENSMUSG00000029235	Pdcl2	9.180175352	11.9673395	35.94089384	15.27448758	5.85E-06	0.424989085
ENSMUSG00000029484	Anxa3	2.188366419	2.447006069	3.501163271	1.319714322	0.003536068	0.376935955
ENSMUSG00000029563	Foxp2	0	0.004979288	0.039465509	0	0.034898996	0
ENSMUSG00000029616	Erp29	8.858570836	9.971572382	9.379820141	5.758523055	0.036800866	0.61392681
ENSMUSG00000029784	1700025E21Rik	0.166991201	0.301945481	0.731758848	0.145163655	0.015857937	0.198376357
ENSMUSG00000029911	Ssbp1	29.008137	28.50904206	42.51575721	26.55377406	0.046643654	0.624563122
ENSMUSG00000030122	Ptms	202.4819971	153.2513593	82.24509674	34.92912966	1.54E-05	0.424695587
ENSMUSG00000030613	Ccdc90b	9.187892733	10.86774399	25.7092745	13.93224733	0.003311349	0.541915227
ENSMUSG00000031590	Frg1	39.22193599	34.65851544	55.34178366	30.15557123	0.005761869	0.544896988

ENSMUSG00000031917	Nip7	23.35508471	25.36748391	54.30924464	31.30001138	0.009440139	0.576329345
ENSMUSG00000032221	Mns1	9.287004063	6.414174456	6.708975337	3.612638682	0.032249841	0.538478456
ENSMUSG00000032330	Cox7a2	180.9502426	213.8796518	538.9331886	340.2847111	0.048410573	0.63140426
ENSMUSG00000032397	Tipin	107.3780152	107.6460687	227.4188581	126.9249505	0.004250539	0.55811093
ENSMUSG00000032999	Nlrp4f	13.77303411	16.23168121	20.20318925	12.50751739	0.046488114	0.619086285
ENSMUSG00000033752	Mnd1	21.50923797	21.40887867	25.03092595	13.42997848	0.01152182	0.536535425
ENSMUSG00000033931	Rbm34	13.25834425	12.87324856	18.41292241	11.13434131	0.035306178	0.604702559
ENSMUSG00000034192	Lsm3	92.35648085	110.9621833	161.1875807	99.54162831	0.035086194	0.617551476
ENSMUSG00000034932	Mrpl54	14.35487055	16.980475	33.27517082	20.27546712	0.042877446	0.609327214
ENSMUSG00000035202	Lars2	116.0965569	140.5635118	364.438191	148.1685845	5.53E-05	0.406567117
ENSMUSG00000035235	Trim13	34.58789731	38.72138491	85.35757653	53.03135513	0.035596043	0.621284686
ENSMUSG00000035443	Thyn1	22.30609767	26.08406555	70.23033959	42.52343956	0.025098452	0.605485319
ENSMUSG00000035637	Grhpr	3.524490829	3.403720994	4.426130839	1.984392092	0.011714083	0.448335615
ENSMUSG00000036223	Ska1	8.422812126	10.79970832	29.73361369	15.77716184	0.001006047	0.530617032
ENSMUSG00000037361	0610009D07Rik	45.52541201	55.89441988	257.9354885	128.9366035	0.000100094	0.499879269
ENSMUSG00000038510	Rpf2	23.19988571	27.48754055	78.66490789	47.36599244	0.019258539	0.602123535
ENSMUSG00000038543	BC028528	14.3907274	12.40904192	8.039271603	4.008239971	0.013794549	0.49858248
ENSMUSG00000038722	Bud31	26.89691963	38.67570416	76.40002003	46.29371337	0.027968508	0.605938498
ENSMUSG00000038793	Lefty1	97.01823153	123.7768496	70.19861953	31.08417224	3.18E-06	0.442803184
ENSMUSG00000039001	Rps21	205.5238058	266.1069423	636.0294593	391.0012052	0.032260694	0.614753294
ENSMUSG00000039099	Wdr93	3.467191845	4.201728323	8.186746166	3.438196373	6.16E-05	0.419971049
ENSMUSG00000039221	Rpl22l1	35.43421389	41.80770536	303.8359274	136.0840967	1.79E-06	0.447886785
ENSMUSG00000039246	Lyplal1	15.99041077	18.48628793	41.51430239	25.05672856	0.025094762	0.60356858
ENSMUSG00000039323	Igfbp2	197.9006202	256.1382734	173.2185638	97.69843421	0.014560972	0.564018267
ENSMUSG00000039697	Ncoa7	0.831440484	0.739981875	1.517350776	0.769518961	0.048418844	0.507146385
ENSMUSG00000039737	Prkrip1	6.102386376	7.211736977	7.162281491	3.991036313	0.013163269	0.557229748
ENSMUSG00000040269	Mrps28	36.13213272	47.2621709	58.99425373	31.47330995	0.002559688	0.533497891
ENSMUSG00000040952	Rps19	63.90244701	65.96729363	208.9650784	82.59259879	2.19E-07	0.39524594
ENSMUSG00000041378	Cldn5	3.047918298	2.289813275	2.149003487	0.715561844	0.010566664	0.332973794
ENSMUSG00000041453	Rpl21	60.1248407	62.32701907	128.9399647	65.53143429	0.000412206	0.508232141
ENSMUSG00000041840	Haus1	85.51459448	89.28440382	169.3513968	93.50616322	0.00255921	0.552142852
ENSMUSG00000042541	Shfm1	365.6036634	355.5264865	580.2375708	346.7857101	0.016623354	0.597661592
ENSMUSG00000044141	E130201H02Rik	0.535510739	0.638333365	2.178578134	0.573523665	0.018648086	0.263255954
ENSMUSG00000044609	Gm9294	4.941988464	8.955797181	37.65806237	21.80026575	0.031378833	0.578900357
ENSMUSG00000044627	Swi5	38.81829799	40.15373444	99.01197244	59.33826348	0.013794549	0.599303923

ENSMUSG00000045394	Epcam	26.50275352	27.51075603	44.84941846	26.81198836	0.018342884	0.597822431
ENSMUSG00000045799	Gm9800	304.5459112	247.8053348	232.2786386	100.2511307	2.98E-05	0.43159858
ENSMUSG00000046402	Rbp1	15.0240873	16.08938372	11.88894157	5.347484211	3.44E-05	0.449786398
ENSMUSG00000046721	Rpl14-ps1	3.821105455	2.875039327	11.06764737	4.90992047	0.006094876	0.443628199
ENSMUSG00000046909	1110002N22Rik	20.65106599	25.69106393	53.9526132	30.91414881	0.011765255	0.572987053
ENSMUSG00000047635	2810006K23Rik	3.638594645	3.364274872	7.325897492	4.038587432	0.015150001	0.551275449
ENSMUSG00000047675	Rps8	220.3305542	215.1542478	730.6269202	367.4094681	0.001543426	0.50286878
ENSMUSG00000047676	Rpsa-ps10	137.2266857	153.4478091	100.280501	58.97926275	0.006174008	0.588142881
ENSMUSG00000047844	Bex4	104.8414167	135.2007533	186.6667692	102.1356425	0.004440571	0.547154927
ENSMUSG00000048709	Gm8666	29.61228877	27.29888431	29.6720945	16.03638661	0.045713572	0.540453476
ENSMUSG00000048982	Gphb5	0	0	0.375952389	0	0.019763184	0
ENSMUSG00000049751	Rpl36al	42.6595181	41.98722983	86.36402239	50.86761094	0.010834174	0.588990757
ENSMUSG00000049760	2410015M20Rik	148.7970219	100.2257804	101.3030102	53.20659567	0.000862819	0.525222257
ENSMUSG00000050621	Gm9846	5.08010848	9.733679848	35.20745369	17.0838261	0.009726078	0.485233219
ENSMUSG00000050623	1700019N12Rik	0.931375588	0.870202361	5.236121555	2.283842184	0.040504802	0.436170582
ENSMUSG00000050856	Atp5k	54.74428501	36.33864851	78.84712808	41.59767397	0.002520846	0.527573736
ENSMUSG00000050957	Insl6	1.879218923	2.152051957	2.695350385	0.835277546	0.026948407	0.309895719
ENSMUSG00000051355	Commd1	3.803330507	4.440332622	9.972910952	6.257912665	0.045755114	0.62749108
ENSMUSG00000051627	Hist1h1e	41.96974935	65.74211372	101.5720819	36.13141393	1.87E-09	0.3557219
ENSMUSG00000052013	Btla	2.620586473	2.589231468	9.013556748	5.226658092	0.028465345	0.579866332
ENSMUSG00000052033	Pfdn4	9.071838964	11.55602068	30.76135132	16.28186221	0.002072703	0.529296065
ENSMUSG00000052144	Ppp4r2	14.75662306	16.02778272	27.61297958	16.25836846	0.01863589	0.588794426
ENSMUSG00000052146	Rps10	26.89873827	29.9508187	70.60469865	39.7394509	0.004563281	0.562844282
ENSMUSG00000052565	Hist1h1d	41.13269094	61.16736231	60.73932849	35.33808472	0.021305458	0.581799068
ENSMUSG00000053173	Rpl18-ps2	3.085735362	4.294436399	7.727109402	3.347032421	0.046488114	0.433154527
ENSMUSG00000053916	Nanp	0.225114843	0.154391399	1.950079318	0.505477582	0.002922576	0.259208729
ENSMUSG00000054091	1810037I17Rik	62.62365694	91.27288557	125.3648475	69.78268209	0.006399753	0.556636756
ENSMUSG00000054626	Xlr	0.049158834	0.070776644	1.758274513	0.531978106	0.002158074	0.302556911
ENSMUSG00000054727	1700013H16Rik	0.636182124	0.67647332	8.192840449	3.786206848	0.011765255	0.462136041
ENSMUSG00000055447	Cd47	1.996540857	1.461588861	2.680746829	1.509268039	0.048069456	0.563002826
ENSMUSG00000055555	4930502E18Rik	2.478551635	2.713364935	21.13553211	9.123167415	4.53E-05	0.431650709
ENSMUSG00000055963	Gm11818	0.5930364	0.914708	1.686685588	0.595610555	0.047516841	0.353124826
ENSMUSG00000056300	Gm13247	26.19339646	30.2377671	161.5677448	98.21297787	0.030493751	0.607874907
ENSMUSG00000056436	Cyct	130.2153557	114.2126034	278.4166446	167.9810609	0.015940882	0.603344175
ENSMUSG00000057130	Txnl4a	7.281771545	5.93498871	15.322734	8.249670206	0.004097237	0.538394141

ENSMUSG00000057278	Snrpg	143.9947736	132.2058822	482.6032493	275.3443696	0.004253858	0.570539817
ENSMUSG00000058064	Gm10036	165.7992828	203.5476614	544.5720478	250.7270777	4.04E-06	0.460411214
ENSMUSG00000058407	Txndc9	13.78076744	16.22991949	25.44697973	15.46395415	0.027968508	0.607693106
ENSMUSG00000058773	Hist1h1b	142.1878119	182.8261763	336.9034669	148.7863678	1.39E-05	0.441629079
ENSMUSG00000059058	Gm15431	51.7931413	71.50417339	70.63640864	21.79314598	2.79E-09	0.30852568
ENSMUSG00000059159	Gm8129	5.692254944	7.004972419	55.86775887	26.97408361	0.004681469	0.48282022
ENSMUSG00000059751	Gm9000	189.2019416	321.7296399	953.648139	422.1248149	2.72E-05	0.4426421
ENSMUSG00000060152	Pop5	8.533447345	8.099489671	16.10901629	9.450786916	0.023101303	0.586676849
ENSMUSG00000060288	Ppih	10.90558095	12.71200155	31.06321102	18.39696397	0.01485906	0.592242829
ENSMUSG00000060438	Rps10-ps1	7.374851686	9.932602975	15.26282748	6.057861266	0.000773919	0.396902951
ENSMUSG00000060636	Rpl35a	99.52061966	96.20438617	269.3823064	108.2620512	1.19E-06	0.401889985
ENSMUSG00000060730	Gm10086	40.12889607	40.81063657	75.80879338	29.53023278	1.39E-06	0.389535718
ENSMUSG00000060743	H3f3a	18.16695343	33.28923489	53.02874533	28.56885752	0.002068171	0.538742852
ENSMUSG00000060938	Rpl26	508.7513825	529.257345	2099.506418	802.9812167	1.95E-05	0.382461902
ENSMUSG00000060981	Hist1h4h	292.2996011	463.4248382	728.8475343	393.9958513	0.003140217	0.540573759
ENSMUSG00000061315	Naca	12.87039754	11.27838207	13.83523649	8.335399177	0.01688036	0.602476089
ENSMUSG00000061482	Hist1h4d	856.5900687	1068.133324	2110.665466	1244.524571	0.036067851	0.589636108
ENSMUSG00000061669	Gm6404	3.41887165	4.143917736	10.72732224	2.568449542	0.000209602	0.239430632
ENSMUSG00000062353	Gm15772	10.51958124	12.60618139	86.19870588	27.99394642	1.74E-09	0.324760635
ENSMUSG00000062691	1110001A16Rik	31.77742648	35.57252359	59.27289996	31.46796445	0.001164758	0.530899694
ENSMUSG00000063021	Hist1h2ak	58.34552396	85.14672258	138.2237545	78.23647673	0.007903584	0.566013251
ENSMUSG00000063172	Hspb11	1.199346847	1.379677604	5.480314125	2.550518221	0.007105439	0.465396355
ENSMUSG00000063179	Pstk	3.844781035	4.267747112	12.45293772	7.326000382	0.044785197	0.588294951
ENSMUSG00000063412	Gm10131	5.222874956	6.067250238	8.012212763	2.962166485	0.002370734	0.369706419
ENSMUSG00000063543	Gm5616	2.167810112	3.178374955	3.26304473	0.670940065	0.010489925	0.205617796
ENSMUSG00000063689	Hist2h2ab	18.98410288	29.23942152	80.83352474	44.0539244	0.012352357	0.544995712
ENSMUSG00000063838	Cdc42ep5	9.302562533	10.12771896	17.60694135	9.361926756	0.005448752	0.53171795
ENSMUSG00000063882	Uqcrh	120.0537962	160.5323826	312.3545149	183.1790686	0.019763184	0.586446041
ENSMUSG00000064264	Zfp428	118.1617947	110.3014438	59.93126905	36.51945241	0.033737538	0.609355567
ENSMUSG00000064288	Hist1h4k	247.366758	299.3233886	366.8946697	225.3385751	0.036202057	0.614177838
ENSMUSG00000064339	mt-Rnr2	46355.78883	58486.82405	245585.6001	194783.8474	0.029484886	0.793140344
ENSMUSG00000064344	mt-Tm	67.44837584	56.67032702	673.4556729	186.6485238	8.52E-11	0.277150422
ENSMUSG00000064364	mt-Th	1.621289028	2.50023434	22.87437857	4.847592035	0.04416774	0.211922349
ENSMUSG00000064373	Sepp1	0.786041694	1.011055437	2.39800728	1.130320581	0.032921939	0.471358277
ENSMUSG00000064380		1183.242316	2129.702932	4357.6	2206.442779	0.004440571	0.506343579

ENSMUSG00000064387	Snora73a	749.2966421	1708.624086	3947.255174	2106.128247	0.009706183	0.533567797
ENSMUSG00000064427		53.12121135	93.6634983	291.7253061	145.2776644	0.005207427	0.497994728
ENSMUSG00000064451	Snora23	247.7828875	398.7552415	1986.200603	957.040863	9.77E-05	0.481845017
ENSMUSG00000064495		23.18861172	26.97120507	129.3514333	67.94493963	0.036892065	0.52527396
ENSMUSG00000064605		7.108898257	14.10142786	73.64040511	30.45599564	0.00101271	0.413577242
ENSMUSG00000064620		10.98167837	10.9060699	70.52728282	22.53236693	0.000325978	0.319484404
ENSMUSG00000064634		391.4943535	523.4668856	1848.06566	446.4616762	5.61E-18	0.241583233
ENSMUSG00000064637	Snora20	24.61344476	35.18518884	171.9392147	69.98449947	0.000306896	0.407030471
ENSMUSG00000064682		12.41922291	25.88524694	127.4962301	40.93633646	1.33E-07	0.321078799
ENSMUSG00000064741	Snord14a	36.62816701	53.00488181	141.6929835	52.22134698	0.000214054	0.368552808
ENSMUSG00000064772		5.040428606	29.98237308	83.01034863	41.75411057	0.018358524	0.502998858
ENSMUSG00000064795		8.053513459	41.4626139	201.8734946	51.82749416	2.28E-12	0.256732536
ENSMUSG00000064796		8.840069208	15.17868645	33.74412203	13.90302015	0.000674812	0.412013095
ENSMUSG00000064837	Snora75	33.71686676	50.398362	169.7378324	71.52624596	8.55E-05	0.421392479
ENSMUSG00000064841		66.41829752	146.8156456	191.6608279	102.1629416	0.030073384	0.533040281
ENSMUSG00000064856		33.9843938	43.60120362	216.5053372	121.9920776	0.040524582	0.563459909
ENSMUSG00000064899	Snord118	248.6230969	537.8376254	2367.157209	731.4153179	4.93E-12	0.308984682
ENSMUSG00000064901	Snora21	116.4993375	198.552915	562.3569173	300.4015115	0.011441114	0.534183011
ENSMUSG00000064923		42.76338868	91.83584946	276.768566	76.83117625	1.30E-11	0.277600803
ENSMUSG00000064925	Snora62	21.44873224	33.35808284	246.2241009	49.7498902	1.21E-17	0.202051261
ENSMUSG00000064938		6.945134421	11.43740698	43.22894005	11.06103886	1.71E-05	0.255871156
ENSMUSG00000064941		121.1198068	237.0162515	794.1317035	455.6705782	0.047516841	0.573797238
ENSMUSG00000064945		2845.550584	2093.004829	8918.564026	2471.106184	2.28E-12	0.277074446
ENSMUSG00000065045		20.53932024	50.79104864	156.5657948	71.24017181	0.001725113	0.45501747
ENSMUSG00000065089		283.0088811	343.5437678	1646.729009	730.9864306	9.28E-05	0.443902079
ENSMUSG00000065118		11.45238048	14.35836591	53.54470003	21.8703214	0.020546998	0.408449788
ENSMUSG00000065126	Snord104	260.2100232	247.9876637	299.7263153	152.057639	0.013200246	0.507321617
ENSMUSG00000065145		83.61691049	184.9345388	685.1621874	338.7810023	0.000525264	0.494453735
ENSMUSG00000065176		76.4836944	184.6294903	1825.305502	542.7544284	1.04E-15	0.297349911
ENSMUSG00000065219	Snord32a	4.909248805	4.843827492	45.15245398	5.88034505	2.02E-06	0.130233122
ENSMUSG00000065232		366.289926	882.8051977	3238.654524	1155.744905	4.10E-09	0.356859584
ENSMUSG00000065265		441.4377896	319.0171454	1509.055472	782.7076712	0.00066139	0.51867389
ENSMUSG00000065280		81.08351432	97.86365267	336.5426598	175.0422581	0.00852926	0.520119079
ENSMUSG00000065305		48.016049	63.57696453	157.11499	78.51681046	0.027973503	0.499741052
ENSMUSG00000065353	Snora73b	2456.781279	3882.102501	8610.77248	4318.208204	0.011714083	0.501489061

ENSMUSG00000065362		25.5943377	42.16176238	139.1869894	48.69303969	8.96E-06	0.349839018
ENSMUSG00000065642	Snora69	132.4770014	157.6500627	487.6524975	250.7912488	0.009476443	0.514282712
ENSMUSG00000065653		17.76898943	21.0374746	58.32910145	22.37955623	0.00642679	0.38367737
ENSMUSG00000065687		8.555363994	10.34062291	54.2959188	9.863926878	5.16E-07	0.181669766
ENSMUSG00000065701		992.8496314	1166.636885	4613.175049	2102.965052	0.000175839	0.455860666
ENSMUSG00000065767		1.595726166	5.675704272	25.44006492	5.115303236	6.92E-06	0.201072727
ENSMUSG00000065817		18.31572757	26.65482049	81.37739641	31.87378172	0.002264587	0.391678563
ENSMUSG00000065818	Snord35a	14.98861483	18.79870868	41.83391485	15.5550744	0.036630417	0.371829279
ENSMUSG00000065820		52.78714326	119.7413426	430.395893	187.0793054	1.45E-05	0.434667961
ENSMUSG00000065845		1.876916431	1.79479812	14.63462459	3.775756105	0.007496788	0.258001569
ENSMUSG00000065870		3492.856517	5597.403429	47678.84375	16216.68224	0.000100094	0.340123228
ENSMUSG00000065905		20.50186583	44.65598505	262.1548616	57.55250641	1.87E-17	0.219536293
ENSMUSG00000065922	n-R5-8s1	11.52376181	5.707769966	18.0958446	0.651174542	5.05E-11	0.035984755
ENSMUSG00000065944		12.2611748	76.87921838	88.02803018	23.74959199	1.56E-08	0.269795791
ENSMUSG00000066000	2610305D13Rik	30.67084976	38.34725275	116.2831002	69.49604639	0.026421154	0.597645284
ENSMUSG00000067058	Rps15a-ps5	10.23568398	14.47878711	90.60822599	44.72389549	0.000525264	0.493596415
ENSMUSG00000067288	Rps28	75.09889587	76.65288776	237.7300821	125.4778906	0.000412206	0.527816629
ENSMUSG00000067367	Lyar	34.93368008	30.4778118	43.6511524	26.92071972	0.032351782	0.616724147
ENSMUSG00000067547	Gm7666	21.30615665	25.12834724	22.88070324	12.71856598	0.048462115	0.555864295
ENSMUSG00000067719	Gm10221	103.1148828	102.7645793	33.22844734	16.43911265	0.000739451	0.494730087
ENSMUSG00000068184	Ndufaf2	22.34458848	24.19835524	28.69001062	16.16611805	0.026671758	0.563475499
ENSMUSG00000068396	Rpl34-ps1	38.36475084	52.7151077	105.2963193	53.10533418	0.000612253	0.50434179
ENSMUSG00000068749	Psma5	127.6591819	160.8004699	249.9832817	141.7517267	0.01220392	0.567044827
ENSMUSG00000069117	Gm17352	27.27478762	31.8812689	59.22089782	22.40431132	5.80E-07	0.378317657
ENSMUSG00000069266	Hist1h4b	74.29560419	79.37013901	146.916561	55.22670281	6.90E-09	0.375905224
ENSMUSG00000070063	Snora33	117.4321093	277.2626499	902.5814348	335.3327215	2.55E-08	0.371526278
ENSMUSG00000070167		829.4945428	1252.621584	1553.334518	781.7102727	0.001581156	0.503246573
ENSMUSG00000071415	Rpl23	718.4638838	657.7141141	1066.278047	496.8189267	0.001588727	0.465937499
ENSMUSG00000071516	Hist1h2ai	15.72293784	23.59709552	31.8178366	18.05025992	0.031378833	0.567300038
ENSMUSG00000071653	1810009A15Rik	176.351965	159.0778305	219.5126317	127.037428	0.007232926	0.57872491
ENSMUSG00000074345	Tnfaip8l3	0.05154497	0.088193825	0.300518948	0.029672135	0.047516841	0.098736321
ENSMUSG00000075706	Gpx4	110.8815414	127.6933816	212.7616397	129.7760228	0.042426967	0.609959685
ENSMUSG00000076135		8.695835337	6.983440306	128.3851275	16.75691079	1.13E-16	0.130520654
ENSMUSG00000076948	Mir467e	1.283413356	1.815408519	7.326709318	0.3692484	0.045284431	0.050397577
ENSMUSG00000077167		84.19890848	171.7757582	892.3572606	376.0049542	5.69E-07	0.421361456

ENSMUSG00000077323		147.0610153	159.81605	2247.042183	470.3810565	9.43E-26	0.209333434
ENSMUSG00000077575		289.0441997	513.3484311	759.8713577	379.3583876	0.002429781	0.499240278
ENSMUSG00000077611		55.13455032	117.2475554	343.0190414	151.7417114	1.58E-05	0.442371102
ENSMUSG00000078180		17.2255148	19.19102616	27.49714914	9.105963648	8.38E-08	0.331160281
ENSMUSG00000078182	Gm6083	15.93501461	22.06034302	53.92992902	26.7401752	0.010473424	0.495831826
ENSMUSG00000078183	Gm15610	7.800034223	11.71992298	25.32775873	6.858865404	7.23E-05	0.270804278
ENSMUSG00000078193	Gm2000	104.4036709	58.26674035	344.1655996	121.0590577	1.92E-11	0.351746537
ENSMUSG00000078502	Gm13212	7.407669166	8.404031252	27.10292233	17.12300245	0.047516841	0.631776981
ENSMUSG00000078566	Bnip3	21.135941	27.27242548	54.07260972	25.85310673	3.35E-05	0.478118346
ENSMUSG00000078689	Mup6	2.133723448	2.555793974	18.17325005	7.311045969	5.66E-05	0.40229711
ENSMUSG00000078713	Tomm5	58.61440517	81.19855685	163.6133785	97.12469591	0.01707937	0.593623191
ENSMUSG00000078784	1810022K09Rik	47.27958369	42.70323077	311.0572561	116.2668983	2.32E-10	0.373779733
ENSMUSG00000078861	Zfp931	5.149796112	7.80894589	17.40062084	10.09449895	0.022366456	0.580122919
ENSMUSG00000078872	Gm14401	26.49456716	28.38181403	76.00819321	46.45267898	0.023101303	0.611153575
ENSMUSG00000078905	Gm14393	7.16653771	8.20332167	23.41778351	13.52288645	0.036355144	0.577462271
ENSMUSG00000079316	Rab9	24.57201809	22.6621854	61.2254744	28.60188715	1.80E-05	0.467156644
ENSMUSG00000079523	Tmsb10	27.33746679	33.83839421	76.79388917	41.74620618	0.002327884	0.543613647
ENSMUSG00000079604	Gm13219	0.005530874	0.00682345	0.284238383	0	7.72E-06	0
ENSMUSG00000080152	Gm12260	2.981301137	4.168924308	21.21986361	10.26665631	0.029765737	0.483822917
ENSMUSG00000080303	Gm12419	0.090163175	0.101591966	1.670086038	0.050510394	0.001069407	0.030244187
ENSMUSG00000080465		953.3212434	1374.359448	3639.707395	1019.377945	1.21E-12	0.2800714
ENSMUSG00000080538		704.1183651	775.198764	2116.520274	472.1890849	9.93E-20	0.223096887
ENSMUSG00000080664		42.81393946	59.97518622	474.6758468	283.0692639	0.045755114	0.596342253
ENSMUSG00000080684		12.21983185	17.08488707	28.02436961	9.91693402	0.013204115	0.353868228
ENSMUSG00000080740	Gm15013	4.012391889	3.465597125	29.43062131	10.74740442	1.31E-07	0.365177626
ENSMUSG00000080811	Gm14513	76.25063788	127.1621205	242.6090371	31.78750367	3.21E-31	0.131023576
ENSMUSG00000081049	Rps24-ps3	40.23406768	35.09808719	196.0634339	94.18356194	3.11E-05	0.480372908
ENSMUSG00000081344	Gm14303	57.34288643	63.78482408	142.3454931	81.91663598	0.0106286	0.575477553
ENSMUSG00000081603	Gm14681	284.8086678	240.6798257	471.1560442	251.771581	0.006348121	0.534369842
ENSMUSG00000081627	Gm13158	0.885702407	1.186773798	1.528846483	0.351587181	0.036801745	0.229968925
ENSMUSG00000081684	Rps2-ps13	2.60062675	4.606578162	2.831938801	1.012752741	0.049093396	0.357618159
ENSMUSG00000082419	Gm11425	51.88193951	58.98056564	151.7058108	92.47106115	0.023295434	0.609541986
ENSMUSG00000082765	Gm14411	3.939057065	4.503357662	16.85897082	7.742266583	0.001454342	0.459237202
ENSMUSG00000082895	Rpsa-ps9	15.3381398	13.81210147	22.51667886	9.122237549	1.53E-06	0.405132462
ENSMUSG00000083022	Rps15a-ps6	44.11122058	36.80328784	96.4718769	28.68550444	1.92E-11	0.297345769

ENSMUSG00000083328	Gm11826	2.480596379	4.059452924	21.3178727	7.720773846	0.000873667	0.362173748
ENSMUSG00000083696	Gm12563	36.4855102	57.92629355	104.7529754	28.72728544	7.03E-07	0.274238372
ENSMUSG00000084700		9.276414797	24.44073406	125.0839225	57.76925906	0.006457101	0.461844
ENSMUSG00000084744		43.61486371	52.49764845	122.9127519	52.11582829	0.000775192	0.424006683
ENSMUSG00000084762	Gm14074	73.65772272	71.1527433	109.5324126	64.36803531	0.021389277	0.587661988
ENSMUSG00000085790	Gm12729	0	0	4.375868913	0.052901042	5.55E-10	0.012089266
ENSMUSG00000085893	Gm12091	1.396239996	1.340680913	3.805138772	1.057821888	0.004961153	0.277998242
ENSMUSG00000086147	Gm13150	7.517991827	8.217355804	22.15214357	13.31187663	0.023101303	0.600929503
ENSMUSG00000086324	Gm15564	32.72973765	36.19929305	330.8694397	11.71123016	1.32E-94	0.035395321
ENSMUSG00000086470	Gm12580	0.067229101	0.019133718	29.55964843	0	1.83E-101	0
ENSMUSG00000086567	Gm16247	121.9156302	117.2966347	393.8567014	211.0909886	0.000867044	0.53595886
ENSMUSG00000088025		1047.096421	2478.607771	91.14515071	44.40031456	0.003133859	0.487138528
ENSMUSG00000088059		219.6730586	298.2834291	288.1931571	132.9894562	0.000514771	0.461459452
ENSMUSG00000088108	Snora47	39.01461244	53.44484889	137.1467282	59.84668679	0.001797133	0.436369774
ENSMUSG00000088148	Mir1983	30.01747615	26.86746668	121.8756572	56.4675352	0.011356424	0.463320867
ENSMUSG00000088208		6.700885999	9.325822859	48.58731491	16.82125661	0.001094012	0.346206755
ENSMUSG00000088252	Snord13	30.06034762	38.84113024	168.5181046	39.448179	5.55E-10	0.23408867
ENSMUSG00000088323		53.69605248	49.43879134	119.0516391	60.37565961	0.039182002	0.507138415
ENSMUSG00000088595		2828.264972	1328.488609	1504.080408	24.95277714	2.83E-126	0.016590055
ENSMUSG00000088604		256.1485118	348.7276405	841.7587722	272.7418896	2.77E-10	0.324014312
ENSMUSG00000088675		5.352486267	13.09967744	5.665997567	1.492564646	0.018464726	0.263424865
ENSMUSG00000088712		9.615108295	16.3618449	58.4617007	13.87504049	5.65E-06	0.23733556
ENSMUSG00000089855	Gm15662	27.58264266	29.92085627	75.05889246	22.68840792	1.39E-06	0.30227475
ENSMUSG00000090137	Uba52	9.374530045	9.423626557	30.59643221	16.67700809	0.001307299	0.545063816
ENSMUSG00000090553	Snrpe	157.1142428	221.3377805	341.3034005	175.1133022	0.00255921	0.513072246
ENSMUSG00000091537	Ccdc72	20.40086129	22.89755989	44.89285908	21.09637787	0.000862819	0.469927251
ENSMUSG00000091993	B930036N10Rik	1.174022709	2.029381561	10.08801974	5.223176576	0.034056781	0.517760344
ENSMUSG00000092072	Gm4540	7.495047021	6.670251562	28.0079525	15.16840909	0.048144237	0.541575079
ENSMUSG00000092680	Snord55	8.099662861	11.72289945	125.9960371	12.33374682	1.39E-15	0.097889958
ENSMUSG00000092773		95.03915676	181.7677839	398.0061266	197.6372554	0.000395847	0.496568375
ENSMUSG00000092887	Snord53	24.0261841	26.53837798	62.12582693	26.74470305	0.036441448	0.430492508
ENSMUSG00000093044	Snord8	23.94933558	36.3309924	89.83770813	36.12829248	0.003012286	0.402150647
ENSMUSG00000093077	Mir5105	62472.03134	118606.2593	285859.0771	87242.83648	0.010657952	0.305195264
ENSMUSG00000093098	Mir5102	855.357693	978.5680877	2799.190648	871.5289313	1.80E-12	0.31135033
ENSMUSG00000093107		20.5076952	14.67863828	56.75219887	19.40548858	0.002447943	0.341933687

ENSMUSG00000093183		41.19184786	92.28322362	149.4294659	90.83518433	0.030110056	0.607880004
ENSMUSG00000093413	Snora15	277.2533302	438.3700646	998.9007823	333.007117	1.39E-09	0.333373567
ENSMUSG00000093834		32.33652705	79.56590261	86.46442965	43.70381562	0.036179049	0.505454275
ENSMUSG00000093842		8.573660654	13.86638699	35.18462747	11.16532042	0.000300854	0.317335189
ENSMUSG00000093843		43.26100917	108.3396984	545.9412864	249.0543548	2.98E-05	0.456192563
ENSMUSG00000093956		18.57998956	37.57488885	141.5376573	60.18648482	0.000306372	0.425233015
ENSMUSG00000094131		12.30529751	12.04781731	132.5114659	68.23252167	0.026816267	0.514917869
ENSMUSG00000094327		14.92327984	17.14704216	27.0393242	10.50343461	0.031825494	0.388450337
ENSMUSG00000094330		12.54633587	7.78210832	19.26244458	5.114782147	0.005448752	0.26553131
ENSMUSG00000094344	Gm11942	678.4649816	546.17828	2615.08199	1184.94666	2.98E-05	0.453120271
ENSMUSG00000094377		79.32335089	146.9102635	821.1364385	288.2107258	9.30E-10	0.350990057
ENSMUSG00000094405		19.56266241	27.74703078	284.342871	130.1184129	0.001104389	0.457610956
ENSMUSG00000094411		59.5616647	74.95829449	486.1086848	131.0236145	1.23E-11	0.269535638
ENSMUSG00000094655		524.3112907	1010.475524	8571.864065	1744.030068	3.57E-21	0.203459837
ENSMUSG00000094705		29.81065905	42.67250081	128.0039648	38.71273985	2.79E-09	0.302433912
ENSMUSG00000094812		17.92432107	47.68739758	121.2474052	43.48228345	1.42E-05	0.358624445
ENSMUSG00000095513		3.401911557	4.87312391	23.45215458	4.915672886	0.000214054	0.209604319
ENSMUSG00000095580		8.212342601	28.77491408	47.10079241	20.95967952	0.031946594	0.444996325
ENSMUSG00000095590		162.6123429	219.8695538	1264.053393	558.7716448	4.95E-05	0.442047502
ENSMUSG00000095676		29.89058822	34.946899	517.8111655	221.6475927	3.61E-05	0.428047148
ENSMUSG00000095692		0.797185499	0.588456197	1.74213618	0.238898656	0.036800866	0.137129725
ENSMUSG00000095699		2.022240544	4.342042088	7.364044274	0.68062372	0.030073384	0.092425262
ENSMUSG00000095701		6.689809793	18.25075612	75.737241	29.27756082	0.000535966	0.386567565
ENSMUSG00000095868		64.78073468	164.7354365	139.4594765	54.75114863	0.000883426	0.392595398
ENSMUSG00000095892		6.399308227	8.520152373	127.704887	35.39578959	4.02E-08	0.277168638
ENSMUSG00000096010	Hist4h4	80.40961259	102.5947045	122.3519299	61.46743166	0.001253816	0.502382199
ENSMUSG00000096205		8.538158051	15.7446082	36.8486521	16.14723619	0.039169447	0.438204256
ENSMUSG00000096243		129.8325763	243.9895794	1436.399105	517.717908	1.43E-09	0.360427618
ENSMUSG00000096255	Dynlt1b	1.97653135	2.464704041	4.58792482	1.654391424	0.022366456	0.36059689
ENSMUSG00000096280		14.09978836	20.0522377	209.7637769	92.39052795	0.001148226	0.440450345
ENSMUSG00000096349		4235.361596	6985.199753	41687.13883	10014.12452	4.20E-10	0.24022096
ENSMUSG00000096428		21.16674511	34.76645549	135.1905671	53.5346209	1.09E-05	0.395993759

List of genes upregulated (p-value < 0.05) after LIF treatment (1h) in Stat3 null cells cultured in 2i

ENSEMBL_ID	GENE SYMBOL	Stat3_null_2i (FPKM)	Stat3_null_2i+LIF_1h (	Stat3_wt_2i (FPKM)	Stat3_wt_2i+LIF_1h (	Adj_pvalue	Fold Change
ENSMUSG00000002325	Irf9	0.995737078	3.752201388	0.913760545	3.390133322	7.10E-07	3.768265208
ENSMUSG00000007872	Id3	2.301893318	6.177045605	4.124194418	3.746879946	0.011826551	2.683463025
ENSMUSG00000064387	Snora73a	749.2966421	1708.624086	3947.255174	2106.128247	0.034117206	2.28030394
ENSMUSG00000064717		3.017399025	18.24056179	17.21761953	12.51169032	0.003339894	6.045127489
ENSMUSG00000064772		5.040428606	29.98237308	83.01034863	41.75411057	5.53E-07	5.948377693
ENSMUSG00000064795		8.053513459	41.4626139	201.8734946	51.82749416	3.16E-07	5.14838823
ENSMUSG00000064841		66.41829752	146.8156456	191.6608279	102.1629416	0.04407075	2.210469872
ENSMUSG00000064899	Snord118	248.6230969	537.8376254	2367.157209	731.4153179	0.030924048	2.163264926
ENSMUSG00000065045		20.53932024	50.79104864	156.5657948	71.24017181	0.046869976	2.472869017
ENSMUSG00000065145		83.61691049	184.9345388	685.1621874	338.7810023	0.02043451	2.211688255
ENSMUSG00000065176		76.4836944	184.6294903	1825.305502	542.7544284	0.005898041	2.413971916
ENSMUSG00000065232		366.289926	882.8051977	3238.654524	1155.744905	0.006366396	2.410126883
ENSMUSG00000065251		2.069870485	16.03737474	15.57301493	7.016417136	3.45E-05	7.748008804
ENSMUSG00000065622		1.048357525	10.65548016	15.09844155	8.264851507	0.000118633	10.16397546
ENSMUSG00000065649	Snora74a	479.8601781	1028.121107	2085.736856	1342.142337	0.031378351	2.142543086
ENSMUSG00000065820		52.78714326	119.7413426	430.395893	187.0793054	0.031229884	2.268380805
ENSMUSG00000065883		33.44991262	79.38662874	193.1985982	321.0491672	0.016418566	2.373298539
ENSMUSG00000065944		12.2611748	76.87921838	88.02803018	23.74959199	9.96E-11	6.270134765
ENSMUSG00000070063	Snora33	117.4321093	277.2626499	902.5814348	335.3327215	0.012001962	2.361046324
ENSMUSG00000076281		56.23141157	199.6320925	185.344471	195.5910341	2.87E-05	3.550188177
ENSMUSG00000084379	Gm12957	10.69530336	25.60639946	25.76915543	41.77311983	0.04407075	2.394172338
ENSMUSG00000084722		13.00738984	34.87538271	58.33509599	69.98168275	0.004504655	2.681197622
ENSMUSG00000084729		70.9237249	169.0006837	217.1410923	239.0033874	0.003382569	2.382851209
ENSMUSG00000084749		4.544224038	14.90915908	16.83985045	28.73426145	0.012001962	3.280903176
ENSMUSG00000087775		3.808848324	13.65700714	1.779538895	2.590568976	0.01549514	3.585600155
ENSMUSG00000087943		102.0897524	235.191834	170.4513548	317.0848615	0.029890281	2.303775143
ENSMUSG00000088025		1047.096421	2478.607771	91.14515071	44.40031456	0.012001962	2.367124671
ENSMUSG00000088246		804.3717242	1730.307522	1261.478199	1222.292248	0.01549514	2.151129223
ENSMUSG00000089255	Snora78	112.272552	269.6481984	335.3674439	305.0598819	0.005898041	2.401728593
ENSMUSG00000092674		95.77635609	227.917221	216.0800986	227.4318044	0.004673372	2.379681482
ENSMUSG00000092702		24.01910391	74.99113662	50.84026622	91.81898614	3.45E-05	3.122145476
ENSMUSG00000092805		25.23495954	63.34817484	79.79165969	78.78407084	0.005898041	2.510333917

ENSMUSG00000093056		71.8557525	204.0393964	168.9040715	128.3151153	5.90E-05	2.839569405
ENSMUSG00000093064		10.35196628	29.52844586	34.08235163	27.33240904	0.008793648	2.852448034
ENSMUSG00000093112		292.9887318	885.1747016	960.7454496	1091.740835	1.61E-05	3.021190256
ENSMUSG00000093183		41.19184786	92.28322362	149.4294659	90.83518433	0.025782133	2.240327356
ENSMUSG00000093277		0.396446587	6.096238788	0.442910333	1.660797039	0.034117206	15.37720082
ENSMUSG00000093834		32.33652705	79.56590261	86.46442965	43.70381562	0.031419354	2.460558071
ENSMUSG00000093843		43.26100917	108.3396984	545.9412864	249.0543548	0.005898041	2.504326656
ENSMUSG00000094812		17.92432107	47.68739758	121.2474052	43.48228345	0.030384098	2.660485571
ENSMUSG00000095580		8.212342601	28.77491408	47.10079241	20.95967952	0.00772767	3.503861868
ENSMUSG00000095868		64.78073468	164.7354365	139.4594765	54.75114863	0.01549514	2.542969562
ENSMUSG00000096887		64.40309421	150.2692786	264.8496848	594.4436503	0.010668259	2.333261785

List of genes downregulated (p-value < 0.05) after LIF treatment (1h) in Stat3 null cells cultured in 2i

ENSEMBL_ID	GENE SYMBOL	Stat3_null_2i (FPKM)	Stat3_null_2i+LIF_1h	Stat3_wt_2i (FPKM)	Stat3_wt_2i+LIF_1h	Adj_pvalue	Fold Change
ENSMUSG00000064350	mt-Ty	101.9363193	30.43081038	45.0672999	276.9400227	0.00627727	0.298527655
ENSMUSG00000064363	mt-Nd4	307.8405759	118.451784	272.2964826	616.6803702	0.030924048	0.384782882
