# Supplementary material for: Identification of the missing pluripotency mediator downstream of leukaemia inhibitory factor
Source: EMBO J. 2013 Aug 13;32(19):2561–74. doi: 10.1038/emboj.2013.177 (PMC3791366; doi:10.1038/emboj.2013.177)
Supplement: Supplementary Table S5 [file emboj2013177s3.pdf]

Gene down-regulated upon Tfcp2l1 Knockdown<br>Data from Nishiyama et al, 2013 -<br>Series GSE26520 - FC <0.75	Genes upregulated upon Tfcp2l1 over-expression<br>Data from Correa Cerro et al., 2011 -<br>http://lgsun.grc.nia.nih.gov/ANOVA/output/public-TF_indu	Genes bound by Tfcp2l1<br>Data from ES Cell ChIP-compendium<br>http://bioinformatics.cscr.cam.ac.uk/ES_Cell_ChIP-seq_co	Genes Upregulated after Tfcp2l1 OE, downregualted after Tfcp2l1 KD and bound by Tfcp2l1
39691	35673	0610007P08RIK	2200002K05RIK
1110036E04RIK	38595	0610007P14RIK	2300002M23RIK
1110051M20RIK	40786	0610009B22RIK	4930583H14RIK
1600010M07RIK	0610006I08RIK	0610010O12RIK	8430419L09RIK
1700001K23RIK	0610007C21RIK	0610011F06RIK	ABLIM1
1700003E24RIK	0610009L18RIK	0610011L14RIK	ADIPOQ
1700003M02RIK	0610009O20RIK	0610012H03RIK	AFF1
1700012B09RIK	0610010E21RIK	0610040J01RIK	AGRP
1700013H16RIK	0610010O12RIK	100043580	AGTPBP1
1700014N06RIK	0610011F06RIK	1110002B05RIK	ALOX12
1700016C19RIK	0610011L14RIK	1110002L01RIK	ANKRD35
1700019N12RIK	0610031G08RIK	1110003E01RIK	APOBEC3
1700026N04RIK	0610031J06RIK	1110004E09RIK	APOD
1700028I16RIK	0610037P05RIK	1110004F10RIK	ARHGAP9
1700049G17RIK	0610040B10RIK	1110007L15RIK	B3GALNT1
1700086P04RIK	0710008K08RIK	1110008L16RIK	B4GALNT1
1700090G07RIK	1110001A16RIK	1110012J17RIK	BC028528
1700109G14RIK	1110001J03RIK	1110014N23RIK	BMP7
1700110C19RIK	1110003E01RIK	1110017F19RIK	BTNL1
1700127D06RIK	1110005A23RIK	1110018G07RIK	CABP1
1810007E14RIK	1110008E08RIK	1110020G09RIK	CAPN5
1810007I06RIK	1110012L19RIK	1110020P15RIK	CAR8
1810022C23RIK	1110014K08RIK	1110021J02RIK	CBS
1810043G02RIK	1110017I16RIK	1110021L09RIK	CCDC19
1810062G17RIK	1110020P15RIK	1110028C15RIK	CCDC68
1810063I02RIK	1110032A03RIK	1110032A13RIK	CCNG2
2010004A03RIK	1110032O16RIK	1110034B05RIK	CD97
2010106C02RIK	1110039B18RIK	1110034G24RIK	CDC42EP2
2010205J10RIK	1110054O05RIK	1110036O03RIK	CDC42EP4
2010209O12RIK	1110065P20RIK	1110037F02RIK	CENPB
2200002K05RIK	1110067I12RIK	1110038B12RIK	CKMT1
2210409D07RIK	1190002N15RIK	1110038D17RIK	CMTM7
2300002M23RIK	1190005F20RIK	1110038F14RIK	COBL
2310016D23RIK	1200003C05RIK	1110051M20RIK	COL13A1
2310042E22RIK	1200009I06RIK	1110059E24RIK	CPA2
2310051F07RIK	1200014J11RIK	1110067D22RIK	CPT1A
2410004A20RIK	1200015F23RIK	1190002A17RIK	CRTAC1
2610028H24RIK	1300017J02RIK	1190002H23RIK	DCLK3
2610203C20RIK	1500005A01RIK	1190002N15RIK	DKKL1
2610203C22RIK	1500009C09RIK	1190003M12RIK	DLGAP3
2700008G24RIK	1500009L16RIK	1190005I06RIK	DNAHC10
2810434M15RIK	1500010J02RIK	1190007F08RIK	DOCK10
2900006F19RIK	1500011H22RIK	1200009I06RIK	DUOX1
3300002A11RIK	1600014C23RIK	1200009O22RIK	E030011O05RIK
4430402I18RIK	1600021P15RIK	1200011I18RIK	ENO1
4831440D22RIK	1700001C19RIK	1200014M14RIK	ESRRB
4833432E10RIK	1700001L05RIK	1200016B10RIK	FBXO6
4921511C10RIK	1700001P01RIK	1300002K09RIK	FFAR2
4930412L05RIK	1700007N14RIK	1300010F03RIK	FXYD4
4930458A03RIK	1700008P20RIK	1300014I06RIK	GALNT6

4930465M20RIK
4930519G04RIK
4930522L14RIK
4930542C12RIK
4930550L24RIK
4930558O21RIK
4930583H14RIK
4930583K01RIK
4931403E03RIK
4931406H21RIK
4931420L22RIK
4932412D23RIK
4932425I24RIK
4933406B17RIK
4933406F09RIK
4933411K20RIK
4933413A10RIK
4933424L07RIK
4933424L21RIK
4933432B09RIK
4933432I09RIK
4933436H12RIK
5430427N15RIK
5730403I07RIK
5730416O20RIK
5730507A09RIK
5830444B04RIK
6030455H03RIK
6330500D04RIK
6430514L14RIK
8030451F13RIK
8430419L09RIK
8430427H17RIK
8430430B14RIK
9030625G05RIK
9130208D14RIK
9230109A22RIK
9530008L14RIK
9630026C02RIK
9930005F22RIK
A
A030004J04RIK
A2M
A330023F24RIK
A330048O09RIK
A430089I19RIK
A630010A05RIK
A630081D01RIK
A630095N17RIK
A730006G06RIK
ABCA4
ABCA8B
ABCC3
1700009P17RIK
1700011J10RIK
1700012A16RIK
1700013D24RIK
1700016F12RIK
1700016M24RIK
1700019B21RIK
1700019N12RIK
1700020D05RIK
1700020O03RIK
1700021F05RIK
1700021K14RIK
1700021P04RIK
1700021P22RIK
1700022C21RIK
1700023D19RIK
1700025E21RIK
1700025K23RIK
1700026H06RIK
1700026L06RIK
1700028B04RIK
1700028I16RIK
1700029J07RIK
1700029P11RIK
1700030J22RIK
1700037C18RIK
1700040I03RIK
1700042O10RIK
1700049E22RIK
1700049L16RIK
1700052K11RIK
1700065D16RIK
1700071A11RIK
1700072H12RIK
1700084J12RIK
1700108F19RIK
1700113A16RIK
1700120B06RIK
1700125H20RIK
1700127D06RIK
1810009J06RIK
1810009O10RIK
1810011O10RIK
1810020D17RIK
1810021J13RIK
1810027O10RIK
1810030N24RIK
1810035I16RIK
1810049H13RIK
1810055E12RIK
1810055G02RIK
1810059H22RIK
1810063I02RIK
1300017J02RIK
1500001M20RIK
1500009L16RIK
1500010J02RIK
1500031L02RIK
1500032L24RIK
1600021P15RIK
1600029D21RIK
1700001C02RIK
1700001C19RIK
1700001K19RIK
1700001O22RIK
1700001P01RIK
1700003M02RIK
1700007B14RIK
1700007G11RIK
1700007K13RIK
1700008A04RIK
1700008J07RIK
1700008O03RIK
1700009J07RIK
1700009P17RIK
1700010I14RIK
1700011E24RIK
1700011H14RIK
1700011I03RIK
1700011L22RIK
1700012A16RIK
1700013B16RIK
1700013G24RIK
1700016C15RIK
1700016D06RIK
1700016H13RIK
1700016K19RIK
1700017B05RIK
1700018C11RIK
1700019D03RIK
1700019E19RIK
1700019O17RIK
1700020C07RIK
1700020D05RIK
1700020I14RIK
1700020N01RIK
1700020O03RIK
1700021F05RIK
1700021F07RIK
1700021K19RIK
1700022A21RIK
1700022C21RIK
1700022P22RIK
1700024G13RIK
1700024P04RIK
1700025G04RIK
GCM2
GFRA3
GGT5
GJB3
GPI1
GRIK4
GSS
HAP1
HEBP1
HES1
HK1
IDH2
ILDR1
INHA
INHBB
ITPKA
JAK3
KCNJ12
KDELR3
KDR
KIT
KLHDC7A
KRT23
LAPTM5
LDHB
LDLR
LGALS3
LONRF2
LY6G6E
LY6K
LY75
MOBKL2C
MRAS
MT2
MTAP1A
MYL4
MYO1F
MYOM2
MYOT
NANOG
NFATC2IP
NID1
NKX6-3
NQO1
NSG2
PADI4
PDE8A
PECAM1
PFKP
PIK3IP1
PKM2
PPP1R14D
PQLC1

ABCD2
ABCG5
ABHD2
ABLIM1
ABLIM2
ACCN1
ACOT5
ACPP
ADAMTSL1
ADCY2
ADCY4
ADH7
ADIPOQ
AF067061
AFF1
AGRP
AGTPBP1
AKAP4
AKP5
ALDOA
ALDOC
ALOX12
AMT
ANK1
ANKRD35
APOBEC3
APOC2
APOD
ARHGAP9
ARMC2
ARX
ASGR2
ATP6
ATP7B
ATP8B4
AU018829
AU019157
AU035318
AW011738
AW146299
AW549542
B020031M17RIK
B230311B06RIK
B3GALNT1
B4GALNT1
BAALC
BATF2
BB146404
BC006662
BC014805
BC026682
BC028528
BC029169

2010004A03RIK
2010109N14RIK
2010111I01RIK
2010200O16RIK
2010316F05RIK
2200001I15RIK
2200001K16RIK
2200002K05RIK
2210010N04RIK
2210011C24RIK
2210015D19RIK
2210403K04RIK
2210408F21RIK
2210409E12RIK
2300002M23RIK
2310002J15RIK
2310003L22RIK
2310005C01RIK
2310005N03RIK
2310008M10RIK
2310010J17RIK
2310011J03RIK
2310014G06RIK
2310014H01RIK
2310016M24RIK
2310030G06RIK
2310034G01RIK
2310039H08RIK
2310040G24RIK
2310043M15RIK
2310046K01RIK
2310051F07RIK
2310065F04RIK
2310067P03RIK
2310069B03RIK
2400006E01RIK
2410003L11RIK
2410004A20RIK
2410015M20RIK
2410018E23RIK
2410018G20RIK
2410076I21RIK
2410091C18RIK
2410114N07RIK
2410137F16RIK
2410137M14RIK
2410152P15RIK
2410198J08RIK
2500002B13RIK
2510039O18RIK
2610002J02RIK
2610019A05RIK
2610027C15RIK

1700026D08RIK
1700029F12RIK
1700029G01RIK
1700029H14RIK
1700029I01RIK
1700029J11RIK
1700029M20RIK
1700029P11RIK
1700030F18RIK
1700034H15RIK
1700039E15RIK
1700040I03RIK
1700040L02RIK
1700042G07RIK
1700052K11RIK
1700054N08RIK
1700057K13RIK
1700065D16RIK
1700065I17RIK
1700066M21RIK
1700067K01RIK
1700067P10RIK
1700072E05RIK
1700073E17RIK
1700081L11RIK
1700084E18RIK
1700086O06RIK
1700090G07RIK
1700094C09RIK
1700096J18RIK
1700096K18RIK
1700102P08RIK
1700106J16RIK
1700106N22RIK
1700108F19RIK
1700112E06RIK
1700113I22RIK
1700113O17RIK
1700125D06RIK
1810007M14RIK
1810010H24RIK
1810011H11RIK
1810011O10RIK
1810012P15RIK
1810013D10RIK
1810013L24RIK
1810019J16RIK
1810020D17RIK
1810022C23RIK
1810022K09RIK
1810032O08RIK
1810033B17RIK
1810035L17RIK

PRDM14
PRKAG1
PRMT8
PTGES
RAPGEF3
RND1
RND2
RPL10A
SCD1
SCGB1C1
SEMA5B
SERPINB1A
SERPINB6C
SFRP1
SGCD
SH3BP1
SLC15A1
SLC16A9
SLC30A10
SLC44A1
SLC44A3
SLC6A8
SLIT2
SMAD7
SMARCD3
SMCR7
SNAP91
SPIC
SPIN4
SPINT1
SPNS3
SPP1
STRA8
SULT5A1
TBX3
TCFCP2L1
TCL1
TEAD2
TFPI
TGFBI
TGM2
TMEM117
TRAIP
TRIM16
TRIML1
TUBB2A
VEGFA
WFDC2
WIPI1
WSCD2
ZAP70
ZFP459
ZFP516

BC049265	2610029G23RIK	1810041L15RIK
BC049352	2610029I01RIK	1810055G02RIK
BC051142	2610036L11RIK	1810063B07RIK
BC065085	2610205E22RIK	2010001E11RIK
BC080695	2610209M04RIK	2010001M09RIK
BCL9	2700029M09RIK	2010002N04RIK
BHMT2	2700038C09RIK	2010005J08RIK
BIN2	2700060E02RIK	2010007H12RIK
BMP7	2700099C18RIK	2010011I20RIK
BTNL1	2810001A02RIK	2010106G01RIK
C130074G19RIK	2810004N23RIK	2010107G12RIK
C130090K23RIK	2810008D09RIK	2010107G23RIK
C230093N12RIK	2810025M15RIK	2010107H07RIK
C330004P14RIK	2810048G17RIK	2010109I03RIK
C330024D12RIK	2810405K02RIK	2010110P09RIK
C330026N13RIK	2810416G20RIK	2010111I01RIK
C430049B03RIK	2810417H13RIK	2010204K13RIK
C530044C16RIK	2810422J05RIK	2010300C02RIK
C80719	2810422O20RIK	2010305A19RIK
C86695	2810442I21RIK	2200001I15RIK
CABP1	2810451A06RIK	2200002K05RIK
CABP4	2900042B11RIK	2210009G21RIK
CABP7	2900083I11RIK	2210010C04RIK
CACNG8	3010026O09RIK	2210010C17RIK
CALR4	3100002J23RIK	2210018M11RIK
CAMK2B	3100002L24RIK	2210020M01RIK
CAMK2N1	3110009E22RIK	2210021J22RIK
CAPN5	3110040N11RIK	2210023G05RIK
CAR8	3110043A19RIK	2210403K04RIK
CASR	3300001G02RIK	2210404J11RIK
CBLN1	3321401G04RIK	2210404O07RIK
CBS	3830408C21RIK	2210407C18RIK
CCDC148	4632419K20RIK	2210417A02RIK
CCDC19	4833426J09RIK	2300002M23RIK
CCDC60	4921524J17RIK	2310001K24RIK
CCDC68	4921530G04RIK	2310004N24RIK
CCDC79	4922501C03RIK	2310007B03RIK
CCNG2	4930401A07RIK	2310007H09RIK
CD164L2	4930402D18RIK	2310008H04RIK
CD302	4930402H24RIK	2310008H09RIK
CD37	4930405M20RIK	2310009B15RIK
CD86	4930412M03RIK	2310014L17RIK
CD97	4930429E23RIK	2310016C08RIK
CDC42EP2	4930430E16RIK	2310016E02RIK
CDC42EP4	4930431P19RIK	2310016M24RIK
CDH20	4930432K21RIK	2310022A10RIK
CDX1	4930434E21RIK	2310028H24RIK
CENPB	4930455B14RIK	2310028O11RIK
CHI3L1	4930479M11RIK	2310030G06RIK
CHRNG	4930480K15RIK	2310030N02RIK
CHST2	4930484I04RIK	2310033P09RIK
CKMT1	4930500J02RIK	2310042E22RIK
CLDN10	4930500O09RIK	2310043J07RIK

CLEC4B1
CMTM7
CNR2
CNTNAP3
COBL
COL13A1
COX2
CPA1
CPA2
CPB2
CPNE4
CPT1A
CRTAC1
CSF2RB2
CST7
CTSG
CXCL17
CXCR4
D130059P03RIK
D330027H18RIK
D430041D05RIK
D630039A03RIK
D730048J04RIK
DAZL
DCHS2
DCLK3
DEFB42
DKKL1
DLEU7
DLGAP1
DLGAP2
DLGAP3
DLX4
DNAHC10
DNAHC17
DNAJB13
DOCK10
DUB1
DUOX1
DUSP13
E030011O05RIK
E130113E03RIK
E130116L18RIK
EG266459
EG328314
EG333669
EG381936
EG382919
EG384356
EG434225
EG449630
EG545893
EG545929
4930503E14RIK
4930504H06RIK
4930509E16RIK
4930513N10RIK
4930518J21RIK
4930519N13RIK
4930524J08RIK
4930528F23RIK
4930535I16RIK
4930539J05RIK
4930550G17RIK
4930558O21RIK
4930570C03RIK
4930579G22RIK
4930583H14RIK
4930592I03RIK
4931420L22RIK
4931428F04RIK
4932415G12RIK
4932425I24RIK
4932442K08RIK
4933403G14RIK
4933413A10RIK
4933421I07RIK
4933426I21RIK
4933426K21RIK
4933432G23RIK
4933437F05RIK
5031439G07RIK
5133400G04RIK
5330417C22RIK
5330426P16RIK
5330437I02RIK
5430407P10RIK
5530401N12RIK
5730405O15RIK
5730409G15RIK
5730419I09RIK
5730437N04RIK
5730446C15RIK
5730455P16RIK
5730593F17RIK
5730596B20RIK
5830405N20RIK
5830482F20RIK
6030442E23RIK
6030455H03RIK
6230427J02RIK
6330403L08RIK
6330407J23RIK
6330514A18RIK
6330534C20RIK
6330549H03RIK
2310044G17RIK
2310044H10RIK
2310045N01RIK
2310046A06RIK
2310046K01RIK
2310046O06RIK
2310047M10RIK
2310057M21RIK
2310061C15RIK
2310067B10RIK
2310079N02RIK
2400003C14RIK
2410002F23RIK
2410002I01RIK
2410015M20RIK
2410016O06RIK
2410017P07RIK
2410042D21RIK
2410075B13RIK
2410076I21RIK
2410089E03RIK
2410127L17RIK
2410129H14RIK
2410137F16RIK
2410137M14RIK
2510009E07RIK
2510012J08RIK
2510027J23RIK
2510039O18RIK
2610002I17RIK
2610018G03RIK
2610019F03RIK
2610020H08RIK
2610021K21RIK
2610027L16RIK
2610028A01RIK
2610028E06RIK
2610034B18RIK
2610034M16RIK
2610035D17RIK
2610036D13RIK
2610039C10RIK
2610044O15RIK
2610101N10RIK
2610109H07RIK
2610203C22RIK
2610204M08RIK
2610301B20RIK
2610301F02RIK
2610306M01RIK
2610528E23RIK
2610528J11RIK
2700023E23RIK

EG620899
EG622644
EG624918
EG625262
EG625670
EG628871
EG629798
EG633089
EG653016
EG665871
EG666272
EG666920
EG667483
EG668730
EGFBP2
EHF
ENO1
ENSMUSG00000060247
EPHB6
ESRRB
ESX1
EYA2
F7
FADS6
FATE1
FBXL2
FBXO11
FBXO6
FER1L3
FFAR2
FXYD4
GALNT6
GCAP3
GCET2
GCM2
GDI1
GDPD4
GFAP
GFRA1
GFRA3
GGT5
GJB1
GJB3
GJB4
GLS
GM1019
GM1307
GM1408
GM1467
GM1524
GM257
GM26
GM340
6430514L14RIK
6430527G18RIK
6430531B16RIK
6530437J22RIK
6720401G13RIK
6720460F02RIK
8030402F09RIK
8030423J24RIK
8030474K03RIK
8430416G17RIK
8430419L09RIK
8430430B14RIK
9030607L17RIK
9030617O03RIK
9030625G05RIK
9130011E15RIK
9130017N09RIK
9130230N09RIK
9230109A22RIK
9230110K08RIK
9330129D05RIK
9330159F19RIK
9330161A08RIK
9330199F22RIK
9430069I07RIK
9430081H08RIK
9530020O07RIK
9530048O09RIK
9530077C05RIK
9530080O11RIK
9630033F20RIK
A130015J22RIK
A130022J15RIK
A2M
A430018G15RIK
A430089I19RIK
A530001N23RIK
A530082C11RIK
A630010A05RIK
A630018P17RIK
A630054L15RIK
A630081D01RIK
A730063M14RIK
A730085A09RIK
A730085K08RIK
A830012C17RIK
A830035A12RIK
A930002C04RIK
A930012O16RIK
AA415398
AARS
AB030242
ABCA2
2700060E02RIK
2700062C07RIK
2700078E11RIK
2700081O15RIK
2700094K13RIK
2810002N01RIK
2810006K23RIK
2810008D09RIK
2810008M24RIK
2810021J22RIK
2810025M15RIK
2810030E01RIK
2810055F11RIK
2810055G20RIK
2810408A11RIK
2810408P10RIK
2810410L24RIK
2810422O20RIK
2810429I04RIK
2810432D09RIK
2810453I06RIK
2810459M11RIK
2810474O19RIK
2900005J15RIK
2900006K08RIK
2900010M23RIK
2900026A02RIK
2900041M22RIK
2900052N01RIK
2900097C17RIK
3010026O09RIK
3110001D03RIK
3110003A17RIK
3110079O15RIK
3110082D06RIK
3110082I17RIK
3110099E03RIK
3230401D17RIK
3300002I08RIK
3830406C13RIK
3930402G23RIK
4432412L15RIK
4631416L12RIK
4632411B12RIK
4632428N05RIK
4732418C07RIK
4732471D19RIK
4732471J01RIK
4831426I19RIK
4833403I15RIK
4833420G17RIK
4833439L19RIK
4833442J19RIK

GM347	ABCA3	4921504E06RIK
GM364	ABCA7	4921506M07RIK
GM443	ABCB6	4921515J06RIK
GM5	ABCB9	4921517D22RIK
GM575	ABCC10	4921517L17RIK
GM749	ABCC3	4921521F21RIK
GM799	ABCF3	4921523A10RIK
GM807	ABHD11	4921524J17RIK
GM885	ABHD4	4921528O07RIK
GM967	ABI3	4921530L21RIK
GMCL1L	ABLIM1	4921539E11RIK
GOLT1A	ACAA2	4922501L14RIK
GP9	ACACB	4922505E12RIK
GPI1	ACAD10	4922505G16RIK
GPR182	ACAD11	4930404N11RIK
GPR64	ACADS	4930412C18RIK
GPR83	ACAT3	4930412O13RIK
GRIK4	ACBD4	4930417O13RIK
GRM2	ACO2	4930420K17RIK
GSS	ACOT8	4930422I07RIK
H60A	ACOT9	4930426L09RIK
HAMP	ACP5	4930429B21RIK
HAP1	ACP6	4930434E21RIK
HDRPA	ACSS2	4930442L01RIK
HEBP1	ACTN1	4930444A02RIK
HES1	ACTN4	4930452B06RIK
HESX1	ACTR10	4930453N24RIK
HFE	ACTR1A	4930455C21RIK
HHIP	ACTR2	4930465K10RIK
HIST2H2BB	ADAM15	4930470P17RIK
HK1	ADAM8	4930471G03RIK
HNF4A	ADAM9	4930471M23RIK
HOXC12	ADAMTS10	4930473A06RIK
HOXD10	ADAMTS20	4930483J18RIK
HSPB2	ADCK5	4930486L24RIK
HSPB6	ADCY3	4930488L21RIK
HUS1B	ADD1	4930503L19RIK
HYAL3	ADD3	4930506M07RIK
ICAM2	ADFP	4930507D05RIK
ID4	ADIPOQ	4930511I11RIK
IDH2	ADPRH	4930511M06RIK
IFLD5	AEBP2	4930515G01RIK
IGH-V3660	AES	4930519F16RIK
IGK-V19-14	AFAP1L1	4930519G04RIK
IGK-V23	AFF1	4930523C07RIK
IL16	AFMID	4930524B15RIK
IL1RL2	AFTPH	4930525F21RIK
IL2RB	AGA	4930526D03RIK
IL6RA	AGPAT4	4930528F23RIK
ILDR1	AGPS	4930529M08RIK
INHA	AGRP	4930534B04RIK
INHBB	AGTPBP1	4930538K18RIK
INPPL1	AHCYL1	4930539E08RIK

IQCH
IQCK
ITPKA
JAK3
KCNH1
KCNJ12
KCTD4
KDELR3
KDR
KIF21B
KIFC5C
KIT
KLHDC7A
KLK1
KLK10
KLK11
KLK1B1
KLK1B11
KLK1B24
KLK1B26
KLK1B3
KLRA1
KRT12
KRT16
KRT17
KRT23
KRT27
KRT79
LAPTM5
LAYN
LDHAL6B
LDHB
LDHD
LDLR
LEFTY2
LGALS3
LIM2
LOC215996
LOC233184
LOC235509
LOC330599
LOC382044
LOC385472
LOC432591
LOC432691
LOC432715
LOC433944
LOC434061
LOC434136
LOC434335
LOC434660
LOC434746
LOC434846
AHNAK
AHNAK2
AI413582
AI464131
AI467606
AI662250
AI837181
AI842396
AIFM1
AIFM2
AIM1L
AIP
AK2
AKAP7
AKT1S1
ALDH16A1
ALDH7A1
ALDOC
ALG5
ALG8
ALKBH2
ALKBH5
ALKBH7
ALOX12
ALPK3
ALPL
ALS2CR13
ALS2CR2
AMDHD2
AMHR2
AMPD3
ANAPC10
ANAPC11
ANGEL1
ANGPTL4
ANKK1
ANKRD1
ANKRD28
ANKRD35
ANKRD45
ANKRD52
ANP32A
ANXA1
ANXA11
ANXA5
AP1S1
AP2A1
AP3M2
AP4B1
AP4M1
APH1B
APH1C
APIP
4930539J05RIK
4930547C10RIK
4930555I21RIK
4930563D23RIK
4930567H12RIK
4930571K23RIK
4930579E17RIK
4930579G22RIK
4930579K19RIK
4930583H14RIK
4930583K01RIK
4930588N13RIK
4931406P16RIK
4931408A02RIK
4931429I11RIK
4931432M23RIK
4931440L10RIK
4931440P22RIK
4932412H11RIK
4932416H05RIK
4932418E24RIK
4932431H17RIK
4932435O22RIK
4932438H23RIK
4932441K18RIK
4932443I19RIK
4933401F05RIK
4933402J07RIK
4933403G14RIK
4933404M02RIK
4933406M09RIK
4933407C03RIK
4933407H18RIK
4933407P14RIK
4933411K20RIK
4933412E24RIK
4933413N12RIK
4933421E11RIK
4933421I07RIK
4933426M11RIK
4933427D06RIK
4933427D14RIK
4933432B09RIK
4933432I09RIK
4933433P14RIK
4933437F05RIK
4933439C10RIK
4933439F18RIK
5031414D18RIK
5031425F14RIK
5031439G07RIK
5033406O09RIK
5033411D12RIK

LOC435145
LOC435492
LOC435497
LOC435798
LOC544808
LOC545238
LOC546096
LOC547322
LOC620509
LOC622924
LOC625360
LOC627530
LOC627695
LOC627905
LOC629424
LOC630555
LOC639975
LOC640779
LOC668758
LOC673289
LOC673656
LOC674232
LOC674794
LOC675151
LOC675388
LOC676330
LONRF2
LPAR1
LRCH1
LRFN1
LRRC15
LRRC18
LRRN3
LY6G6E
LY6K
LY75
MAK
MAPK4
MCAM
MEOX2
MESDC1
MESDC2
MFRP
MFSD4
MGC118608
MIER1
MITF
MOBKL2C
MORN1
MRAP
MRAS
MS4A6B
MT2

APOA1BP
APOBEC2
APOBEC3
APOC1
APOC4
APOD
APOE
APOL8
APOM
ARAF
ARBP
ARF2
ARF5
ARFIP2
ARHGAP17
ARHGAP30
ARHGAP9
ARHGEF10L
ARHGEF12
ARHGEF15
ARHGEF16
ARIH2
ARL1
ARL13B
ARL15
ARL3
ARL4D
ARL6IP3
ARL8B
ARMC10
ARMC5
ARPC1B
ARRDC2
ARSA
ARVCF
AS3MT
ASB10
ASGR2
ASL
ASNA1
ASNS
ASPSCR1
ASRGL1
ASS1
ATAD4
ATG12
ATG4B
ATG9B
ATMIN
ATP11B
ATP12A
ATP1B1
ATP2A3

5033414D02RIK
5033414K04RIK
5133401N09RIK
5330413P13RIK
5330417C22RIK
5330426P16RIK
5330437I02RIK
5430407P10RIK
544988
5730403M16RIK
5730409E04RIK
5730410E15RIK
5730419I09RIK
5730422E09RIK
5730437N04RIK
5730455O13RIK
5730469M10RIK
5730494N06RIK
5730508B09RIK
5730522E02RIK
5730528L13RIK
5730559C18RIK
5730601F06RIK
5830405N20RIK
5830416P10RIK
5830418K08RIK
5830433M19RIK
5930412G12RIK
6030405A18RIK
6030419C18RIK
6030458C11RIK
6030468B19RIK
6130401L20RIK
6330403A02RIK
6330403K07RIK
6330406I15RIK
6330407J23RIK
6330408A02RIK
6330409N04RIK
6330512M04RIK
6330545A04RIK
6330577E15RIK
6430527G18RIK
6430531B16RIK
6430537H07RIK
6430548M08RIK
6430562O15RIK
6430571L13RIK
6430573F11RIK
6430598A04RIK
6430704M03RIK
6530402F18RIK
6530411M01RIK

MTAP1A
MUC4
MYBPC3
MYBPH
MYL4
MYL7
MYLC2PL
MYO1F
MYOCD
MYOD1
MYOM2
MYOT
MYPN
NANOG
NANOS2
NCF2
ND1
NDUFA4L2
NEBL
NFATC2IP
NFE2
NFE2L3
NID1
NKX6-3
NMU
NOBOX
NOTCH4
NQO1
NSG2
NXF2
OAS1A
OAS1D
OBOX6
OBSCN
OGDHL
OIT3
OLFR1042
OLFR1044
OLFR1054
OLFR1310
OLFR20
OLFR220
OLFR23
OLFR309
OLFR339
OLFR494
OLFR584
OLFR638
OLFR676
OLFR787
OLFR921
OLFR935
OLFR954
ATP5C1
ATP5G1
ATP5S
ATP6AP1
ATP6V0B
ATP6V0E2
ATP6V1A
ATP6V1B2
ATP6V1H
ATPBD1B
ATXN3
AU021092
AURKA
AW061290
AW146299
AW549542
AYM1
AZIN1
B230206L02RIK
B230311B06RIK
B230312A22RIK
B230369F24RIK
B330016D10RIK
B3GALNT1
B3GAT3
B3GNT2
B3GNT8
B430105G09RIK
B4GALNT1
B4GALNT4
B4GALT1
B4GALT5
B930041F14RIK
B9D1
B9D2
BAD
BAMBI
BAT2
BAT4
BB146404
BBS2
BBS4
BBS5
BBS7
BC003266
BC003993
BC008155
BC008163
BC011487
BC013491
BC017647
BC018507
BC020002
6720401G13RIK
6720456B07RIK
6720457D02RIK
7420416P09RIK
8430406I07RIK
8430410K20RIK
8430419L09RIK
8430426H19RIK
8430427H17RIK
8430429K09RIK
9030224M15RIK
9030409G11RIK
9030420J04RIK
9030425E11RIK
9030617O03RIK
9030624J02RIK
9030625A04RIK
9130011E15RIK
9130011J15RIK
9130014G24RIK
9130019O22RIK
9130024F11RIK
9130404D08RIK
9230110C19RIK
9230116N13RIK
9330111N05RIK
9330129D05RIK
9330175E14RIK
9430015G10RIK
9430020K01RIK
9430023L20RIK
9430038I01RIK
9430060I03RIK
9430070O13RIK
9430076C15RIK
9430083A17RIK
9530026P05RIK
9530068E07RIK
9530077C05RIK
9630025I21RIK
9630028H03RIK
9630033F20RIK
9830001H06RIK
9930013L23RIK
9930023K05RIK
A
A130010J15RIK
A130022J15RIK
A130040M12RIK
A130042E20RIK
A130049A11RIK
A230020J21RIK
A230051G13RIK

OLFR961
OLFR984
OPN4
OSBPL6
OTTMUSG00000000712
OTTMUSG00000000990
OTTMUSG00000010086
OTTMUSG00000010537
OTTMUSG00000015643
OTUD6A
PADI4
PAM
PAQR6
PBP2
PCDHB10
PCDHB15
PCK1
PDE11A
PDE4A
PDE5A
PDE6C
PDE8A
PDE9A
PECAM1
PFKP
PGLYRP1
PIK3IP1
PILRA
PIP5K1B
PKD2L1
PKM2
PLA2G4E
PLCD4
PLD1
PLEKHG1
POLN
PPP1R14D
PPP1R3G
PQLC1
PRAMEL4
PRAMEL6
PRDM14
PRG4
PRKAG1
PRMT8
PROK2
PROKR1
PRSS34
PRSS35
PSAP
PSG28
PSG-PS1
PTGER2
BC021614
BC022960
BC024978
BC025076
BC028528
BC029169
BC030183
BC031181
BC032203
BC037703
BC046404
BC049762
BC050196
BC051227
BC055111
BC057022
BC057893
BC061212
BC066028
BC099439
BCAN
BCAP29
BCAS2
BCDIN3D
BCKDHB
BCL2L1
BCL3
BET1L
BEX1
BEX2
BEX4
BEX6
BHLHB2
BIN2
BIRC3
BLM
BLNK
BLVRA
BLVRB
BMP5
BMP7
BPHL
BRCA1
BRCA2
BRCC3
BRE
BRP44
BRP44L
BSCL2
BSG
BTBD1
BTNL1
BUB1B
A230056P14RIK
A230065H16RIK
A230069A22RIK
A2BP1
A330040F15RIK
A330050F15RIK
A3GALT2
A430005L14RIK
A430078G23RIK
A430107O13RIK
A4GALT
A4GNT
A530013C23RIK
A530053G22RIK
A530054K11RIK
A630007B06RIK
A730008H23RIK
A730011L01RIK
A730017L22RIK
A830018L16RIK
A830080D01RIK
A930001N09RIK
A930004D18RIK
A930011G23RIK
A930015D03RIK
AA408296
AA543186
AA881470
AA960436
AAAS
AACS
AAMP
AANAT
AARS
AARS2
AASDH
AASDHPPT
AATK
ABAT
ABCA1
ABCA12
ABCA13
ABCA3
ABCA5
ABCA7
ABCA8B
ABCB10
ABCB6
ABCB9
ABCC4
ABCC5
ABCC6
ABCC9

PTGES	C030039L03RIK	ABCD3
PTGS1	C030044B11RIK	ABCD4
PTP4A3	C030046E11RIK	ABCF2
PTPN20	C130034I18RIK	ABCG1
PTPN5	C130039O16RIK	ABCG4
PTPRD	C130040J23RIK	ABHD10
PYGL	C130069I09RIK	ABHD12
RAET1E	C1QTNF4	ABHD14B
RAPGEF3	C1S	ABHD2
RASD1	C2	ABHD3
RASSF8	C230093N12RIK	ABHD5
RAX	C330001K17RIK	ABHD6
RBM44	C330002G04RIK	ABI1
RBM47	C330004M20RIK	ABI2
RIPK5	C330005M16RIK	ABI3
RLN1	C330006K01RIK	ABL1
RND1	C330016O10RIK	ABL2
RND2	C330024D21RIK	ABLIM1
RNF135	C330048F19	ABLIM2
RP23-233H9.5	C430049B03RIK	ABLIM3
RP23-394O9.3	C530044N13RIK	ABR
RPL10A	C730025P13RIK	ABRA
RPTN	C78283	ABT1
RRAS	C920006O11RIK	ABTB2
RUNDC2A	CAB39L	ACAA2
S100A13	CABC1	ACACA
SAA1	CABP1	ACACB
SAA2	CACNG7	ACADM
SAA3	CADM4	ACADSB
SAMM50	CALB2	ACAP2
SCD1	CALCOCO2	ACAP3
SCGB1C1	CALML4	ACBD3
SDC4	CALR	ACBD4
SEC16B	CAMK1D	ACBD6
SEC31B	CANT1	ACCN1
SEMA4B	CAPN1	ACCN2
SEMA5B	CAPN5	ACCN4
SEMA6C	CAPNS1	ACCS
SERPINB1A	CAPSL	ACCSL
SERPINB1B	CAR13	ACE
SERPINB1C	CAR8	ACER1
SERPINB6C	CASP3	ACER2
SFI1	CASQ1	ACHE
SFRP1	CAST	ACLY
SGCD	CAT	ACMSD
SH3BP1	CATSPER2	ACO1
SIDT2	CBLC	ACOT1
SLC13A5	CBR3	ACOT11
SLC15A1	CBR4	ACOT12
SLC16A9	CBS	ACOT2
SLC30A10	CCBL2	ACOT7
SLC44A1	CCDC103	ACOT8
SLC44A3	CCDC104	ACOXL

SLC46A2
SLC5A3
SLC6A13
SLC6A7
SLC6A8
SLIT2
SMAD7
SMARCD3
SMCR7
SNAP91
SNCG
SNF1LK
SNORD116
SNRPD3
SORBS3
SORL1
SPARCL1
SPEER4B
SPEG
SPIC
SPIN4
SPINT1
SPNS3
SPP1
SSTR3
ST3GAL3
ST8SIA6
STAB1
STRA8
SULT1A1
SULT5A1
SYT10
SYT8
TAAR3
TAC2
TAOK1
TBX3
TCFCP2L1
TCL1
TCSTV1
TCSTV3
TCTEX1D1
TDPOZ1
TDPOZ3
TDPOZ4
TEAD2
TEX13
TEX18
TFF3
TFPI
TGFBI
TGM2
TGM3

CCDC113
CCDC115
CCDC130
CCDC14
CCDC19
CCDC22
CCDC24
CCDC28B
CCDC3
CCDC32
CCDC44
CCDC5
CCDC55
CCDC68
CCDC77
CCDC85B
CCDC88A
CCDC88C
CCDC89
CCDC94
CCDC96
CCHCR1
CCNA1
CCND1
CCND3
CCNE1
CCNF
CCNG2
CCRK
CCT8
CD109
CD248
CD2BP2
CD38
CD55
CD59A
CD59B
CD63
CD68
CD79B
CD9
CD97
CDC123
CDC25B
CDC2L1
CDC37L1
CDC42EP1
CDC42EP2
CDC42EP4
CDC42EP5
CDC6
CDK2
CDK5RAP1

ACP2
ACP5
ACP6
ACPL2
ACRBP
ACSBG1
ACSBG2
ACSF2
ACSF3
ACSL1
ACSL3
ACSL4
ACSL5
ACSL6
ACSS1
ACTB
ACTBL2
ACTG1
ACTL6A
ACTL6B
ACTL7A
ACTN1
ACTN4
ACTR10
ACTR1B
ACTR2
ACTR3
ACTR6
ACTR8
ACTRT2
ACVR1
ACVR1B
ACVR2B
ACVRL1
ACY1
ACY3
ACYP1
ACYP2
ADA
ADAM11
ADAM12
ADAM15
ADAM17
ADAM19
ADAM22
ADAM23
ADAM24
ADAM6B
ADAM9
ADAMTS1
ADAMTS14
ADAMTS16
ADAMTS17

THEM5
TINAGL
TMEM117
TMEM132D
TMEM178
TNFRSF13C
TNFRSF1B
TPO
TRAIP
TRF
TRH
TRIM16
TRIM38
TRIM66
TRIM7
TRIML1
TRPC7
TRPM1
TRPS1
TSLP
TTYH2
TUBB2A
TYRO3
UBE2K
UCP1
UGCG
USP46
V1RH18
VAX1
VAX2
VAX2OS1
VEGFA
VGLL2
VIT
WDR6
WFDC2
WFIKKN1
WFIKKN2
WIPI1
WSCD2
XAF1
XM_145358
ZAN
ZAP70
ZBP1
ZC3H12A
ZFP148
ZFP169
ZFP295
ZFP459
ZFP516
ZFP518
ZFP526
CDK7
CDKAL1
CDKN1A
CDKN2D
CDO1
CDS2
CDYL2
CEACAM20
CEBPD
CEBPE
CELSR1
CENPB
CENPH
CENPM
CENPN
CENTA1
CENTB1
CEP55
CEP68
CEPT1
CETN2
CETN3
CETN4
CFD
CGA
CGGBP1
CHAF1B
CHCHD1
CHD2
CHEK1
CHID1
CHKB
CHRNA4
CHRNA9
CHST5
CIB1
CIRBP
CITED2
CITED4
CKLF
CKMT1
CKMT2
CLCN2
CLCNKB
CLDN3
CLDN4
CLEC11A
CLGN
CLN6
CLYBL
CMBL
CMPK1
CMTM5
ADAMTS19
ADAMTS2
ADAMTS20
ADAMTS5
ADAMTS7
ADAMTS8
ADAMTS9
ADAMTSL1
ADAMTSL2
ADAMTSL4
ADAMTSL5
ADAP1
ADAP2
ADAR
ADARB1
ADARB2
ADAT1
ADAT3
ADC
ADCK1
ADCK2
ADCY1
ADCY2
ADCY3
ADCY6
ADCY7
ADCY9
ADD1
ADD3
ADH1
ADH5
ADH7
ADHFE1
ADI1
ADIPOQ
ADIPOR1
ADIPOR2
ADK
ADM
ADNP
ADNP2
ADORA1
ADORA2B
ADPGK
ADPRHL1
ADPRHL2
ADRA1B
ADRA1D
ADRA2A
ADRA2B
ADRA2C
ADRB1
ADRB2

ZFP820
ZFYVE28
ZSCAN2
ZSWIM5

CMTM6
CMTM7
CNDP2
CNN3
CNTROB
COASY
COBL
COG4
COIL
COL13A1
COL16A1
COL3A1
COPS4
COPS7A
COPZ1
COQ6
COQ7
CORO7
COX1
COX11
COX17
COX18
COX2
COX5A
COX6C
COX7A1
COX7A2L
COX8A
CPA2
CPD
CPLX3
CPNE9
CPSF4L
CPT1A
CPT1B
CPVL
CREB5
CREBL2
CREG1
CRTAC1
CRXOS1
CRY2
CRYAB
CRYZL1
CSDE1
CSF1
CSGALNACT1
CSL
CSNK1E
CSPG5
CSRP1
CSTF3
CTBS

ADRB3
ADRBK1
ADRBK2
ADSS
AEBP1
AEBP2
AEN
AES
AF529169
AFAP1L1
AFAP1L2
AFF1
AFF3
AFF4
AFMID
AFTPH
AGAP1
AGAP3
AGBL3
AGBL4
AGFG1
AGFG2
AGGF1
AGL
AGPAT2
AGPAT3
AGPAT4
AGPAT5
AGPAT6
AGPAT9
AGPS
AGRN
AGRP
AGTPBP1
AGTRAP
AGXT
AHCTF1
AHCY
AHCYL1
AHCYL2
AHDC1
AHI1
AHNAK
AHR
AHRR
AHSA1
AI118078
AI314180
AI314831
AI314976
AI414108
AI427809
AI429214

CTH
CTNNA1
CTSA
CTSD
CUEDC1
CUL7
CUX1
CXCR7
CYBASC3
CYC1
CYHR1
CYLC1
CYP2C39
CYP2S1
CYP46A1
CYP4F13
CYP4F39
CYP7B1
D10BWG1070E
D10BWG1364E
D130020L05RIK
D14ERTD668E
D17WSU104E
D19ERTD652E
D330017J20RIK
D330046F09RIK
D430040L24RIK
D4ERTD196E
D5ERTD579E
D630004A14RIK
D730001G18RIK
D730003I15RIK
D730045A05RIK
D730048J04RIK
DACT1
DAF2
DALRD3
DAP3
DAPK1
DBI
DBT
DCLK3
DCP2
DCTN4
DCUN1D5
DDAH2
DDB1
DDIT3
DDIT4L
DDX19A
DDX26B
DDX28
DDX4
AI464131
AI467606
AI480653
AI506816
AI661453
AI836003
AI837181
AI846148
AI854703
AI894139
AICDA
AIG1
AIM1
AIM1L
AIM2
AIPL1
AIRN
AJAP1
AK1
AK129341
AK2
AK3
AK3L1
AK5
AK7
AKAP1
AKAP10
AKAP11
AKAP12
AKAP13
AKAP2
AKAP5
AKAP6
AKAP7
AKAP8
AKAP9
AKR1A4
AKR1B10
AKR1B3
AKR1B8
AKR1C20
AKR1C21
AKR1D1
AKR7A5
AKT1S1
AKT2
AKTIP
ALAS1
ALDH1A2
ALDH1A3
ALDH1B1
ALDH1L1
ALDH2

DDX50
DDX58
DDX59
DEDD2
DEF6
DEFB35
DENND2C
DENND3
DENND4B
DERL2
DET1
DGKA
DHX32
DIAP1
DIS3L
DIXDC1
DKK3
DKKL1
DLG2
DLGAP3
DLGAP4
DLX1AS
DMBT1
DNA2
DNAHC10
DNAJB1
DNAJB5
DNAJC19
DNAJC4
DNAJC8
DNALC4
DNMBP
DNPEP
DOCK10
DOCK5
DPPA3
DRAP1
DSC2
DTX1
DUOX1
DUSP1
DUSP9
DVL2
DYNLT1
DYNLT3
DYX1C1
E030011O05RIK
E130016E03RIK
E130018N17RIK
E130202H07RIK
E230001N04RIK
E2F1
E2F2
ALDH3A1
ALDH4A1
ALDH5A1
ALDH7A1
ALDH8A1
ALDOA
ALDOART2
ALG1
ALG10B
ALG12
ALG13
ALG14
ALG5
ALG6
ALG9
ALK
ALKBH1
ALKBH5
ALLC
ALMS1
ALOX12
ALOX15
ALOX5AP
ALPK1
ALPK3
ALPL
ALS2CL
ALX3
AMAC1
AMBN
AMD1
AMD2
AMDHD2
AMHR2
AMN1
AMOTL1
AMOTL2
AMPD1
AMPD3
AMT
AMTN
AMZ1
AMZ2
ANAPC10
ANAPC13
ANAPC5
ANAPC7
ANG
ANG2
ANGEL1
ANGEL2
ANGPT2
ANGPT4

E330016A19RIK	ANGPTL1
E430016P22RIK	ANGPTL2
E430024P14RIK	ANGPTL4
EAR12	ANK
EBI3	ANK1
ECH1	ANK2
ECM1	ANK3
EDC4	ANKFN1
EDF1	ANKHD1
EDG5	ANKLE1
EED	ANKRA2
EEF1A1	ANKRD1
EFEMP2	ANKRD10
EFHC1	ANKRD11
EFHD2	ANKRD12
EG212753	ANKRD13A
EG229879	ANKRD13C
EG234159	ANKRD16
EG238829	ANKRD17
EG245297	ANKRD23
EG245347	ANKRD28
EG271505	ANKRD33
EG333830	ANKRD33B
EG382645	ANKRD34A
EG385521	ANKRD35
EG432743	ANKRD43
EG434280	ANKRD44
EG434402	ANKRD45
EG434426	ANKRD46
EG434459	ANKRD50
EG545052	ANKRD55
EG545208	ANKRD56
EG546038	ANKRD58
EG546143	ANKRD6
EG547215	ANKRD9
EG620155	ANKS1
EG622052	ANKS1B
EG624866	ANKS3
EG625969	ANLN
EG626367	ANO10
EG627420	ANO2
EG627782	ANO6
EG628847	ANO9
EG629591	ANP32A
EG638580	ANP32B
EG653016	ANP32E
EG665513	ANTXR1
EG665897	ANTXRL
EG666332	ANUBL1
EG666435	ANXA1
EG666525	ANXA11
EG666560	ANXA2
EG667027	ANXA3

EG667179
EG667483
EG667489
EG667802
EG668730
EG668804
EGFBP2
EGLN3
EHD1
EIF2A
EIF2AK2
EIF2AK3
EIF2B1
EIF2B4
EIF2S2
EIF2S3X
EIF4A2
ELA1
ELOVL6
ELOVL7
EMP1
EMP3
ENG
ENO1
ENO2
ENSMUSG00000049830
ENSMUSG00000071543
ENSMUSG00000073728
ENSMUSG00000074379
ENSMUSG00000075025
EOMES
EPHB3
EPHX1
EPM2AIP1
EPN2
ERAF
ERBB2IP
ERLIN1
ERMN
ERO1L
ESD
ESRRA
ESRRB
ESX1
ETNK2
ETV4
EVI5L
EXDL2
EXOC3L
EXT1
EXTL1
EZH2
EZR

ANXA4
ANXA6
ANXA8
AOAH
AOX1
AOX3
AOX3L1
AOX4
AP1AR
AP1B1
AP1G1
AP1G2
AP1M1
AP1M2
AP1S1
AP1S3
AP2A2
AP2B1
AP2S1
AP3B1
AP3B2
AP3D1
AP3M2
AP3S2
AP4E1
AP4S1
APAF1
APBA2
APBB1
APBB2
APC
APEX1
APH1B
APIP
APITD1
APLF
APLN
APLNR
APLP1
APLP2
APOBEC1
APOBEC2
APOBEC3
APOC1
APOC4
APOD
APOE
APOL7A
APOM
APOO
APP
APPBP2
APPL2

F3
F630043A04RIK
FABP3
FADS2
FAF1
FAH
FAHD1
FAHD2A
FANCA
FANCI
FARS2
FASTKD1
FBLIM1
FBN2
FBP1
FBP2
FBXL3
FBXL8
FBXO15
FBXO2
FBXO24
FBXO27
FBXO36
FBXO44
FBXO45
FBXO6
FBXW10
FDFT1
FDPS
FDXR
FECH
FEM1B
FERD3L
FERMT3
FERT2
FETUB
FEZ1
FFAR2
FGD1
FGFR4
FGG
FHL1
FHL4
FIGNL1
FKBP15
FKBP5
FKBP6
FKBP7
FKBP8
FKBPL
FLOT1
FLOT2
FMN1
AQP1
AQP11
AQP2
AQP3
AQP5
AQP6
AQP7
AQP9
AQR
ARAP1
ARAP3
ARC
ARF1
ARF2
ARF3
ARF4
ARF6
ARFGAP1
ARFGAP2
ARFGAP3
ARFGEF1
ARFGEF2
ARFIP1
ARG2
ARGLU1
ARHGAP1
ARHGAP10
ARHGAP12
ARHGAP15
ARHGAP17
ARHGAP18
ARHGAP19
ARHGAP21
ARHGAP22
ARHGAP23
ARHGAP24
ARHGAP25
ARHGAP26
ARHGAP27
ARHGAP29
ARHGAP5
ARHGAP8
ARHGAP9
ARHGDIA
ARHGEF1
ARHGEF10
ARHGEF10L
ARHGEF11
ARHGEF15
ARHGEF16
ARHGEF17
ARHGEF18
ARHGEF19

FNBP1
FNDC3B
FOXN4
FOXRED1
FRMD4A
FRRS1
FRY
FRYL
FSTL1
FTHL17
FTL1
FTL2
FTSJ1
FUBP3
FURIN
FXR1
FXYD2
FXYD4
FXYD6
G0S2
G6PC3
GAA
GABARAP
GABARAPL2
GABPB1
GADD45A
GADD45B
GAL
GALNT11
GALNT6
GALT
GANAB
GAS2
GAS5
GBAS
GCAP3
GCAT
GCC1
GCHFR
GCM2
GCNT2
GCNT3
GDF15
GDI1
GDI2
GEMIN6
GFM2
GFRA3
GGA1
GGNBP1
GGNBP2
GGT1
GGT5
ARHGEF3
ARHGEF4
ARHGEF7
ARID1A
ARID1B
ARID3A
ARID3B
ARID3C
ARID4A
ARID5A
ARID5B
ARIH2
ARL11
ARL13B
ARL14
ARL15
ARL3
ARL4A
ARL4C
ARL4D
ARL5A
ARL6
ARL6IP1
ARL8B
ARMC10
ARMC2
ARMC4
ARMC6
ARMC7
ARMC8
ARMC9
ARMCX1
ARNT2
ARNTL
ARPC1A
ARPC1B
ARPC2
ARPC3
ARPM1
ARPP21
ARRB1
ARRB2
ARRDC1
ARRDC2
ARRDC3
ARSB
ARSG
ARSI
ART2B
ART5
ARTN
ARVCF
AS3MT

GGT6
GGTA1
GINS2
GIPC1
GIPC2
GJB3
GJB5
GJC1
GLA
GLCE
GLI2
GLIPR2
GLO1
GLOD4
GLRX
GM1335
GM1611
GM1631
GM1964
GM216
GM24
GM2A
GM525
GM555
GM830
GM867
GM9
GMEB1
GMFG
GMPR2
GNA11
GNA14
GNA15
GNAT3
GNB1L
GNB2
GNB4
GNE
GNG2
GNGT2
GNL3L
GNPDA1
GOT1
GPBP1
GPC4
GPI1
GPR124
GPR133
GPR160
GPR20
GPR56
GPR83
GPRC5A

ASAH1
ASAP1
ASAP2
ASAP3
ASB1
ASB10
ASB13
ASB14
ASB15
ASB17
ASB18
ASB2
ASB4
ASB6
ASB7
ASCC1
ASCC2
ASCC3
ASCL1
ASF1A
ASH1L
ASH2L
ASL
ASNS
ASPA
ASPH
ASPHD2
ASPM
ASPRV1
ASPSCR1
ASRGL1
ASS1
ASTN2
ASXL1
ASXL2
ATAD1
ATAD2B
ATAD4
ATAD5
ATCAY
ATE1
ATF1
ATF2
ATF3
ATF6
ATF7
ATF7IP
ATF7IP2
ATG10
ATG16L1
ATG16L2
ATG2B
ATG4C

GPRC5B
GPSN2
GPT2
GPX1
GPX2
GPX2-PS1
GPX7
GRAMD1A
GRASP
GRHL3
GRIK4
GRIN2C
GRIP2
GRN
GRPEL1
GRRP1
GRTP1
GSDMA1
GSDMA2
GSDMD
GSS
GSTA1
GSTA2
GSTA3
GSTA4
GSTZ1
GTF2A2
GTPBP1
GTPBP6
GTRGEO22
GTSF1
GYLTL1B
GYS1
H1F0
H2-M10.1
H2-M5
H2AFX
H2AFY2
H2AFY3
H6PD
HADH
HAGHL
HAL
HAP1
HAPLN3
HAT1
HBB-BH1
HBEGF
HC
HCFC2
HDAC1
HDAC6
HDDC2
ATG5
ATG7
ATIC
ATL2
ATL3
ATMIN
ATN1
ATOH1
ATOH7
ATOH8
ATOX1
ATP10D
ATP11A
ATP11B
ATP11C
ATP12A
ATP13A1
ATP13A2
ATP13A3
ATP1A1
ATP1A2
ATP1A3
ATP1A4
ATP1B1
ATP1B2
ATP1B3
ATP2A2
ATP2A3
ATP2B1
ATP2B2
ATP2B3
ATP2B4
ATP2C1
ATP2C2
ATP4A
ATP5A1
ATP5C1
ATP5E
ATP5F1
ATP5L
ATP5S
ATP6AP1L
ATP6AP2
ATP6V0A1
ATP6V0A2
ATP6V0B
ATP6V0C
ATP6V0D1
ATP6V0D2
ATP6V1A
ATP6V1B1
ATP6V1B2
ATP6V1C1

HDDC3
HEATR5B
HEBP1
HECTD3
HEMK1
HES1
HEXIM2
HFE
HIATL1
HIBADH
HIBCH
HIGD2A
HINT3
HIST1H2BP
HIST2H2AA1
HIST2H2BE
HIST2H3C2
HIVEP3
HK1
HMBS
HMCN2
HMG20A
HMG20B
HMGCL
HNRNPL
HOXA7
HOXC13
HOXD8
HPDL
HPS6
HRASLS3
HRB
HS3ST6
HS6ST1
HSCB
HSD17B1
HSD17B10
HSD17B7
HSF2BP
HSPA1A
HSPA1B
HSPA8
HSPB8
HTATIP2
HUS1B
HUWE1
HYAL1
HYLS1
IAH1
ICA1
ICA1L
ICAM1
IDH1
ATP6V1D
ATP6V1E1
ATP6V1F
ATP6V1H
ATP7B
ATP8A1
ATP8A2
ATP8B1
ATP8B2
ATP9A
ATP9B
ATPIF1
ATRNL1
ATXN1
ATXN2L
ATXN3
ATXN7
ATXN7L1
ATXN7L3
AU017455
AU018091
AU021034
AU022252
AU023871
AU040320
AU040829
AU042651
AUH
AURKAIP1
AURKB
AUTS2
AV249152
AVEN
AVPI1
AVPR1A
AW146154
AW209491
AW549877
AW554918
AW555464
AXIN1
AXIN2
AXL
AYM1
AZI1
AZIN1
B230120H23RIK
B230206F22RIK
B230206H07RIK
B230217C12RIK
B230219D22RIK
B230312A22RIK
B330016D10RIK

IDH2
IDH3A
IDH3G
IDI1
IER3
IER5L
IFI30
IFT172
IFT52
IFT57
IGBP1
IGF2
IGFBP7
IGH-V3660
IGLL1
IGSF21
IGSF5
IHH
IL10
IL13RA1
IL17RC
IL23A
IL4I1
ILDR1
ILKAP
IMMP2L
IMPA2
INHA
INHBB
IPP
IQCC
IQCG
IQGAP2
IRF8
IRF9
ISLR2
ISOC2B
ITGA6
ITPK1
ITPKA
IVNS1ABP
JAK3
JAKMIP1
JAM2
JUP
KALRN
KANK3
KANK4
KAZALD1
KCNH3
KCNJ12
KCNK4
KCNMB2

B3GALNT1
B3GALNT2
B3GALT1
B3GALT5
B3GALTL
B3GNT1
B3GNT2
B3GNT3
B3GNT5
B3GNT7
B3GNT8
B3GNTL1
B430010I23RIK
B430306N03RIK
B4GALNT1
B4GALNT2
B4GALNT3
B4GALNT4
B4GALT1
B4GALT3
B4GALT5
B4GALT6
B830017H08RIK
B930041F14RIK
BACE1
BACE2
BACH1
BACH2
BAD
BAG3
BAHCC1
BAHD1
BAI1
BAI2
BAI3
BAIAP2
BAIAP2L1
BAK1
BAMBI
BANF1
BANF2
BARD1
BARX1
BARX2
BASP1
BAT1A
BAT2D
BAT2L
BAT3
BATF3
BAX
BAZ1A
BAZ2A

KCNS3	BAZ2B
KCTD1	BB014433
KCTD19	BB123696
KCTD6	BBC3
KCTD7	BBS10
KCTD9	BBS12
KDELC2	BBS2
KDELR3	BBS5
KDR	BBS9
KEAP1	BBX
KIF22	BC002163
KIF3C	BC002230
KIFC1	BC003266
KIFC2	BC003267
KIFC3	BC003331
KIRREL2	BC004004
KIRREL3	BC005561
KIT	BC005764
KLB	BC006965
KLF2	BC011426
KLF4	BC013529
KLHDC7A	BC016201
KLHDC9	BC016495
KLHL10	BC017158
KLK1	BC017612
KLK1B1	BC017647
KLK1B24	BC018465
KLK1B26	BC018473
KLK1B3	BC018507
KLK1B5	BC020535
KLK1B9	BC021614
KLK8	BC021785
KLKB1	BC021891
KLRG2	BC022687
KNDC1	BC023744
KNG1	BC024139
KNTC1	BC024479
KRCC1	BC025920
KRT23	BC026439
KRT42	BC026585
KRT7	BC027231
KRT8	BC027344
KRTAP8-2	BC028528
KTELC1	BC030307
LACTB2	BC030336
LAGE3	BC030476
LAMA1	BC030500
LAMB3	BC030867
LAMC2	BC031353
LAMP1	BC031441
LAP3	BC031781
LAPTM5	BC032203
LASP1	BC048403

LCK
LDHAL6B
LDHB
LDHC
LDLR
LEPROT
LETMD1
LGALS2
LGALS3
LGALS8
LGALS9
LGMN
LHX3
LIAS
LINGO1
LIPM
LITAF
LIX1
LIX1L
LLGL2
LMOD3
LNPEP
LOC100043424
LOC208428
LOC218997
LOC219102
LOC219180
LOC227920
LOC228124
LOC232745
LOC235509
LOC237547
LOC238756
LOC240160
LOC241715
LOC245350
LOC269292
LOC270362
LOC278757
LOC380728
LOC381355
LOC382468
LOC382545
LOC382741
LOC383050
LOC383570
LOC383603
LOC384229
LOC384978
LOC386426
LOC432596
LOC432831
LOC432848
BC048507
BC049349
BC049807
BC050777
BC052040
BC055111
BC057022
BC057079
BC059842
BC060267
BC061194
BC066028
BC066135
BC067068
BC068281
BC089491
BCAM
BCAN
BCAP29
BCAP31
BCAR1
BCAR3
BCAS1
BCAS2
BCAS3
BCAT1
BCAT2
BCDIN3D
BCHE
BCKDHA
BCKDHB
BCL11A
BCL11B
BCL2A1C
BCL2L1
BCL2L10
BCL2L11
BCL2L14
BCL2L2
BCL3
BCL6
BCL7A
BCL7B
BCL9
BCL9L
BCLAF1
BCMO1
BCOR
BCORL1
BCR
BDH1
BDKRB1
BDKRB2

LOC433500	BDNF
LOC433944	BEGAIN
LOC434061	BEND3
LOC434156	BEND4
LOC434169	BEND5
LOC435250	BEND6
LOC435657	BEND7
LOC435752	BEST1
LOC435875	BEST3
LOC435963	BET1L
LOC544808	BET3L
LOC545261	BFAR
LOC545785	BFSP1
LOC546500	BFSP2
LOC546736	BHLHA9
LOC546886	BHLHE22
LOC621487	BHLHE23
LOC621842	BHLHE40
LOC622356	BHLHE41
LOC623572	BHMT
LOC624367	BHMT2
LOC624724	BICD1
LOC628133	BICD2
LOC628602	BIN1
LOC628850	BIN3
LOC632296	BIRC2
LOC632985	BIRC3
LOC635340	BIRC6
LOC637800	BIVM
LOC637900	BLCAP
LOC638112	BLK
LOC640324	BLM
LOC640665	BLMH
LOC664999	BLNK
LOC665232	BLOC1S3
LOC665814	BLVRA
LOC666583	BMF
LOC667036	BMP1
LOC667672	BMP10
LOC669236	BMP2K
LOC670325	BMP4
LOC670403	BMP5
LOC670457	BMP6
LOC670838	BMP7
LOC670845	BMP8A
LOC671039	BMP8B
LOC671464	BMPR1B
LOC671478	BMPR2
LOC671520	BNC2
LOC671913	BNIP3L
LOC672238	BOC
LOC672365	BOD1
LOC672650	BOK

LOC673378	BOLA1
LOC673638	BOLA2
LOC674900	BOLL
LOC675151	BOP1
LOC675992	BPTF
LOC676330	BRCA1
LOC676847	BRCC3
LOC677341	BRD1
LONRF1	BRD2
LONRF2	BRD3
LONRF3	BRD4
LOXL4	BRD9
LPIN1	BRDT
LPIN2	BRE
LPL	BRI3
LRCH4	BRI3BP
LRP1	BRIP1
LRP3	BRP16
LRRC2	BRP44L
LRRC27	BRPF1
LRRC28	BRPF3
LRRC29	BRSK2
LRRC34	BRUNOL4
LRRC49	BRUNOL5
LRRC56	BRWD1
LRRC61	BSCL2
LRRC8A	BSDC1
LRRC9	BSN
LRRN2	BSND
LRRN3	BSPRY
LTB4R1	BST1
LY6G6E	BSX
LY6K	BTAF1
LY75	BTBD10
LYCAT	BTBD11
LYN	BTBD12
LYPLA3	BTBD16
LYRM2	BTBD17
LYSMD4	BTBD2
LZTFL1	BTBD3
M6PRBP1	BTBD6
MACROD1	BTBD7
MAD2L2	BTBD9
MAGED2	BTC
MAGOH	BTD
MAN2B1	BTF3
MANBA	BTG1
MAP2K6	BTG2
MAP3K10	BTG3
MAP3K9	BTLA
MAP4K1	BTN1A1
MBC2	BTN2A2
MBD6	BTNL1

MBOAT5
MCAM
MCCC1
MCEE
MCF2
MCM8
MCTS2
MDGA1
MDM4
MED19
MED21
MEI1
MEST
METT5D1
METTL5
METTL9
MFGE8
MFI2
MFN1
MGL2
MIA1
MICALL2
MIF4GD
MINK1
MKRN2
MKS1
MLANA
MLF2
MLXIPL
MLYCD
MMAA
MMD
MMD2
MME
MMP11
MMP23
MMP9
MMRN2
MNAT1
MOBKL2A
MOBKL2C
MOBKL3
MOCOS
MOCS2
MORC4
MORN3
MOV10
MPZL2
MRAS
MRE11A
MRPL1
MRPL14
MRPL2
BTRC
BUB1
BUB1B
BUB3
BUD13
BZW2
C030002C11RIK
C030017K20RIK
C030034L19RIK
C030039L03RIK
C030044B11RIK
C130026L21RIK
C130039O16RIK
C130071C03RIK
C130074G19RIK
C130079G13RIK
C1D
C1GALT1
C1QB
C1QL1
C1QL2
C1QTNF2
C1QTNF3
C1QTNF4
C1QTNF9
C1RB
C1RL
C2
C230035I16RIK
C230081A13RIK
C230096C10RIK
C2CD2
C2CD2L
C2CD3
C2CD4B
C2CD4C
C330005M16RIK
C330007P06RIK
C330016O10RIK
C330019L16RIK
C330021F23RIK
C330023M02RIK
C330024D21RIK
C430004E15RIK
C530005A16RIK
C530008M17RIK
C630004H02RIK
C730027P07RIK
C77080
C77370
C80913
C85492
C86187

MRPL32
MRPL36
MRPL41
MRPL45
MRPL52
MRPL55
MRPS14
MRPS16
MRPS18C
MRPS36
MS4A1
MSLN
MSN
MST1
MST1R
MT1
MT2
MTAP1A
MTAP7
MTERFD3
MTHFD1
MTIF2
MTMR3
MTMR7
MTR
MTRF1
MTRF1L
MTTP
MUS81
MUT
MVK
MVP
MYH13
MYH14
MYH15
MYL4
MYLC2B
MYLPF
MYO10
MYO1C
MYO1F
MYO1G
MYO5C
MYOM2
MYOT
MYST4
N4BP1
NAGA
NAGK
NAGLU
NAGS
NAIF1
NANOG

C86695
C87436
C8B
CAB39
CAB39L
CABC1
CABIN1
CABLES1
CABLES2
CABP1
CABP2
CABP7
CABYR
CACHD1
CACNA1A
CACNA1B
CACNA1C
CACNA1D
CACNA1E
CACNA1G
CACNA1S
CACNA2D1
CACNA2D2
CACNA2D3
CACNB1
CACNB2
CACNB3
CACNG2
CACNG3
CACNG4
CACNG5
CACNG6
CACNG7
CACNG8
CACYBP
CADM1
CADM2
CADM3
CADM4
CADPS
CADPS2
CALB2
CALCOCO1
CALCOCO2
CALCRL
CALD1
CALHM2
CALM1
CALM2
CALML3
CALML4
CALR4
CALU

NAPG
NAPSA
NARFL
NARS
NAT11
NAT13
NAT9
NCAM1
NCF1
NCF4
NCOA4
NCOA6
NDG2
NDRG1
NDRG3
NDUFA13
NDUFA6
NDUFA8
NDUFB11
NDUFB3
NDUFB4
NDUFB5
NDUFV1
NEFL
NEK6
NEK8
NEURL2
NEUROD1
NF2
NFATC2IP
NFE2L2
NFKBIA
NFKBIL2
NFU1
NG23
NGFRAP1
NHLRC3
NID1
NKAIN4
NKIRAS2
NKX6-3
NLRC3
NLRP4C
NLRP4F
NMNAT3
NMU
NNMT
NOL3
NOS3
NOSIP
NOTCH1
NOTCH4
NPC2

CALY
CAMK1
CAMK1D
CAMK2A
CAMK2B
CAMK2D
CAMK2G
CAMK2N1
CAMK4
CAMKK2
CAMKV
CAMSAP1
CAMSAP1L1
CAMTA1
CAND1
CAND2
CANT1
CANX
CAP1
CAP2
CAPG
CAPN1
CAPN10
CAPN11
CAPN13
CAPN2
CAPN5
CAPN8
CAPNS1
CAPRIN1
CAPRIN2
CAPSL
CAPZA2
CAPZB
CAR10
CAR12
CAR13
CAR14
CAR3
CAR6
CAR7
CAR8
CAR9
CARD11
CARD14
CARHSP1
CARKD
CARM1
CARTPT
CASC1
CASC3
CASP3
CASP6

NPCD
NPDC1
NPEPPS
NPPB
NPR1
NPR3
NQO1
NQO2
NR2C2AP
NRARP
NRCAM
NRM
NSDHL
NSG2
NSMCE4A
NSUN4
NSUN6
NT5C3L
NT5DC1
NT5DC3
NTF5
NTHL1
NUBP2
NUBPL
NUDCD1
NUDT1
NUDT13
NUDT16L1
NUDT2
NUDT4
NUP155
NUP62
NUP62CL
NUP85
NUPR1
OAF
OAS2
OASL1
OAZ2
OCRL
OGG1
OGT
OLFR1040
OLFR1109
OLFR1239
OLFR1264
OLFR1268-PS1
OLFR1384
OLFR1428
OLFR1475
OLFR1491
OLFR309
OLFR361
CASP8AP2
CASQ1
CASQ2
CASR
CASS4
CAST
CASZ1
CATSPER1
CATSPER3
CATSPERG1
CATSPERG2
CBARA1
CBFA2T2
CBFA2T3
CBFB
CBLB
CBLC
CBR1
CBR3
CBR4
CBS
CBX2
CBX3
CBX4
CBX5
CBX6
CBX6-NPTXR
CBX7
CBX8
CC2D1B
CCAR1
CCBE1
CCBL2
CCDC101
CCDC105
CCDC108
CCDC109A
CCDC110
CCDC112
CCDC113
CCDC114
CCDC115
CCDC117
CCDC12
CCDC121
CCDC124
CCDC125
CCDC126
CCDC129
CCDC130
CCDC132
CCDC134
CCDC135

OLFR458
OLFR478
OLFR594
OLFR606
OLFR891
OMA1
OOEP
OPLAH
ORC3L
ORC6L
OSBPL7
OSGEPL1
OSGIN1
OSTA
OSTF1
OTTMUSG00000000712
OTTMUSG00000007018
OTTMUSG00000010009
OTTMUSG00000015351
OTTMUSG00000018358
OTTMUSG00000020946
OXNAD1
P2RX5
P2RY5
PABPC3
PADI2
PADI4
PADI6
PAFAH1B1
PANK1
PAPD4
PAQR4
PAQR7
PARP1
PARP2
PASK
PBP2
PBX2
PBX4
PCCA
PCCB
PCDHGC5
PCK2
PCM1
PCOLCE
PCYOX1L
PCYT1A
PDE8A
PDGFC
PDHB
PDK3
PDLIM2
PDXK

CCDC136
CCDC138
CCDC14
CCDC146
CCDC147
CCDC148
CCDC155
CCDC160
CCDC19
CCDC25
CCDC28A
CCDC3
CCDC30
CCDC33
CCDC34
CCDC37
CCDC38
CCDC39
CCDC40
CCDC44
CCDC45
CCDC46
CCDC47
CCDC48
CCDC50
CCDC51
CCDC54
CCDC55
CCDC57
CCDC6
CCDC60
CCDC61
CCDC62
CCDC63
CCDC64
CCDC66
CCDC67
CCDC68
CCDC70
CCDC71
CCDC72
CCDC73
CCDC75
CCDC76
CCDC77
CCDC8
CCDC83
CCDC84
CCDC85A
CCDC85C
CCDC87
CCDC88C
CCDC9

PDXP
PDZD11
PDZRN4
PEBP1
PECAM1
PECR
PEG12
PEPD
PER1
PEX11B
PEX11C
PEX13
PFDN4
PFDN5
PFKFB1
PFKFB3
PFKL
PFKP
PGAM2
PGBD5
PGC
PGM1
PGPEP1
PGRMC1
PHF1
PHF11
PHF19
PHF20
PHLDA1
PHYH
PHYHD1
PI4K2B
PIAS2
PIAS3
PIAS4
PIB5PA
PIGA
PIGC
PIGK
PIGP
PIK3IP1
PIM1
PIN1
PIN4
PINC
PIP4K2C
PIR
PJA2
PKM2
PKN1
PKNOX1
PKNOX2
PKP3
CCDC90A
CCDC91
CCDC92
CCDC93
CCDC96
CCDC97
CCDC99
CCHCR1
CCK
CCKAR
CCKBR
CCL1
CCL17
CCL22
CCL25
CCM2
CCNA1
CCNA2
CCNB1
CCNB1IP1
CCNC
CCND1
CCND2
CCND3
CCNE1
CCNE2
CCNF
CCNG1
CCNG2
CCNH
CCNJL
CCNL1
CCNL2
CCNT2
CCNY
CCNYL1
CCPG1
CCR10
CCR4
CCR6
CCR7
CCR9
CCRK
CCRL1
CCRN4L
CCS
CCT2
CCT4
CCT5
CCT7
CCT8
CCT8L1
CD14

PLA2G2C
PLA2G2E
PLA2G4B
PLA2G4E
PLA2G7
PLAA
PLAUR
PLCG2
PLEC1
PLEKHA2
PLEKHA6
PLEKHA7
PLEKHG6
PLEKHH1
PLEKHO2
PLK3
PLOD1
PLSCR1
PLSCR2
PLSCR4
PLTP
PLXDC1
PLXNA2
PLXNB2
PMEPA1
PMVK
PNCK
PNKD
PNPLA3
PNPLA6
PODNL1
POLD4
POLI
POLQ
POLR2G
POLR2K
POLR3GL
POMT1
PPAP2A
PPARD
PPARGC1B
PPCDC
PPFIA4
PPM1J
PPP1R10
PPP1R11
PPP1R14D
PPP1R3C
PPP2R2A
PPP2R2C
PPP2R4
PPP2R5A
PPP2R5C

CD151
CD164
CD164L2
CD180
CD1D2
CD2
CD209F
CD22
CD247
CD248
CD24A
CD274
CD276
CD28
CD2AP
CD2BP2
CD300A
CD300C
CD300E
CD300LB
CD300LG
CD320
CD33
CD34
CD37
CD38
CD4
CD40
CD47
CD5
CD55
CD59A
CD63
CD68
CD69
CD70
CD72
CD79A
CD79B
CD80
CD83
CD84
CD8B1
CD9
CD93
CD96
CD97
CDA
CDADC1
CDAN1
CDC123
CDC14A
CDC14B

PPP3CC
PPWD1
PQLC1
PRAF2
PRDM10
PRDM14
PRDM16
PRDX1
PRELID1
PRELID2
PRICKLE2
PRICKLE4
PRIM1
PRIM2
PRKAG1
PRKAR2A
PRKCZ
PRMT6
PRMT8
PROC
PROS1
PRPF18
PRPF31
PRPF38A
PRPH
PRPSAP2
PRR13
PRRC1
PRRX2
PRSS36
PSMB7
PSMC3IP
PSMD11
PSMD13
PSMD2
PSMD5
PSMD7
PSMD8
PSPH
PTGES
PTGIS
PTPN11
PTPRN
PTPRV
PTRF
PXN
PYCR1
QARS
QK
QPCTL
QSOX2
RAB1B
RAB27A

CDC16
CDC20
CDC23
CDC25A
CDC25C
CDC27
CDC2A
CDC2L5
CDC2L6
CDC37
CDC42BPA
CDC42BPB
CDC42EP1
CDC42EP2
CDC42EP3
CDC42EP4
CDC42EP5
CDC42SE1
CDC42SE2
CDC45L
CDC6
CDC7
CDC73
CDCA4
CDCA7
CDCA8
CDCP1
CDGAP
CDH1
CDH10
CDH12
CDH13
CDH16
CDH2
CDH22
CDH23
CDH26
CDH29
CDH3
CDH4
CDH5
CDH6
CDK2AP1
CDK2AP2
CDK5R2
CDK5RAP1
CDK5RAP2
CDK5RAP3
CDK6
CDK7
CDKAL1
CDKL1
CDKL2

RAB27B
RAB35
RAB4B
RABAC1
RABGEF1
RABGGTB
RAD51L1
RAD9
RAD9B
RAF1
RAG1AP1
RAGE
RALB
RAP2A
RAPGEF3
RARG
RASA4
RBBP5
RBP1
RBPJ
RBPMS2
RC3H2
RCE1
RCN1
RCOR1
RDH11
REEP5
REL
REN2
REPS2
RGN
RGS14
RHBDD1
RHBDF2
RHD
RHEB
RHOBTB2
RHOC
RHOV
RHOX5
RHOX6
RIN1
RIN3
RLBP1
RMI1
RMND5B
RNASEK
RND1
RND2
RNF125
RNF135
RNF144B
RNF17
CDKL3
CDKL4
CDKN1A
CDKN1B
CDKN1C
CDKN2A
CDKN2AIP
CDKN3
CDNF
CDO1
CDON
CDRT4
CDS1
CDS2
CDSN
CDT1
CDV3
CDX1
CDX2
CDX4
CDYL
CDYL2
CEACAM10
CEACAM15
CEACAM2
CEACAM9
CEBPA
CEBPB
CEBPD
CEBPE
CEBPG
CEBPZ
CECR2
CECR5
CECR6
CELA1
CELA2A
CELA3B
CELSR1
CELSR2
CELSR3
CENPB
CENPF
CENPJ
CENPK
CENPM
CENPQ
CENPT
CENPV
CEP120
CEP135
CEP170
CEP250

RNF181
RNGTT
ROD1
ROPN1L
RP23-136K12.4
RP23-248K2.4
RP23-304I1.3
RP23-54E4.8
RPA3
RPAIN
RPAP1
RPL10A
RPL17
RPL22
RPL26
RPL3
RPL36AL
RPL39
RPL7L1
RPL8
RPP25
RPS15A
RPS3A
RPS8
RPSA
RRAS
RRM1
RRM2
RRM2B
RRP9
RTKN
RTKN2
RTN1
RUNX1
RUSC2
RWDD1
RWDD3
RYR1
RYR2
S100A1
S100A13
S100A6
SAA1
SAA3
SASH3
SAT2
SC4MOL
SCAPER
SCARB1
SCARB2
SCARF1
SCD1
SCGB1C1

CEP290
CEP55
CEP63
CEP68
CEP70
CEP76
CEP78
CEP97
CEPT1
CERK
CERKL
CES1
CETN2
CETN3
CETN4
CFDP1
CFL1
CFL2
CFLAR
CFTR
CGA
CGGBP1
CGNL1
CHAD
CHADL
CHAF1B
CHCHD3
CHCHD5
CHCHD6
CHD1L
CHD2
CHD5
CHD6
CHD7
CHD9
CHGB
CHI3L1
CHIC2
CHID1
CHIT1
CHKA
CHKB-CPT1B
CHL1
CHMP1B
CHMP2A
CHMP2B
CHMP4B
CHMP4C
CHMP6
CHMP7
CHN1
CHN2
CHORDC1

SCGB3A1
SCHIP1
SCN7A
SCNN1A
SCP2
SCRN1
SCUBE2
SCYE1
SDCBP
SDCBP2
SDF2
SDHC
SDHD
SEC22A
SELENBP1
SELENBP2
SELM
SEMA4A
SEMA5B
SENP3
SEPX1
SERHL
SERINC2
SERPINB1A
SERPINB1C
SERPINB6A
SERPINB6C
SERPINE1
SERPING1
SERPINH1
SERTAD2
SERTAD3
SFN
SFRP1
SFRS7
SFRS9
SFT2D3
SFXN4
SGCB
SGCD
SH2D4A
SH2D4B
SH3BP1
SH3TC1
SH3TC2
SHE
SHISA4
SHKBP1
SHMT1
SIAH1A
SIP1
SIRPA
SIRT3

CHPT1
CHRAC1
CHRD
CHRDL2
CHRM1
CHRM3
CHRM4
CHRNA1
CHRNA4
CHRNA7
CHRNA9
CHRNB1
CHRNB2
CHRNB3
CHRNB4
CHRNG
CHST10
CHST11
CHST12
CHST13
CHST14
CHST15
CHST2
CHST3
CHST5
CHST8
CHSY3
CHTF8
CHUK
CIAPIN1
CIB2
CIC
CIDEA
CIITA
CILP
CIRBP
CIRH1A
CISD1
CIT
CITED2
CITED4
CIZ1
CK137956
CKAP2L
CKAP4
CKAP5
CKB
CKLF
CKM
CKMT1
CKS1B
CKS2
CLASP1

SIRT5
SKAP2
SKP2
SLC11A1
SLC15A1
SLC16A9
SLC22A18
SLC22A20
SLC22A7
SLC23A2
SLC25A1
SLC25A11
SLC25A13
SLC25A16
SLC25A19
SLC25A2
SLC25A20
SLC25A36
SLC25A39
SLC25A4
SLC25A43
SLC26A6
SLC27A3
SLC29A1
SLC2A5
SLC2A6
SLC2A8
SLC30A10
SLC30A2
SLC30A3
SLC31A1
SLC34A3
SLC35B1
SLC35B2
SLC35E4
SLC37A4
SLC38A4
SLC39A4
SLC44A1
SLC44A3
SLC44A4
SLC4A11
SLC4A3
SLC6A1
SLC6A3
SLC6A8
SLC7A1
SLC7A5
SLC7A6OS
SLC7A8
SLC9A1
SLC9A8
SLCO2A1
CLASP2
CLCA3
CLCC1
CLCF1
CLCN2
CLCN3
CLCN6
CLDN10A
CLDN14
CLDN18
CLDN2
CLDN23
CLDN3
CLDN4
CLDN5
CLDN7
CLDN9
CLDND1
CLEC11A
CLEC12A
CLEC16A
CLEC2D
CLEC2L
CLEC3A
CLGN
CLIC1
CLIC4
CLIC6
CLINT1
CLIP1
CLIP2
CLIP3
CLMN
CLN5
CLN6
CLN8
CLNK
CLNS1A
CLOCK
CLPB
CLPS
CLPX
CLSTN1
CLSTN2
CLSTN3
CLTA
CLTB
CLTC
CLVS1
CLYBL
CMA2
CMAH
CMAS

SLCO4A1
SLFN9
SLIT2
SMAD7
SMARCC2
SMARCD2
SMARCD3
SMC1B
SMC5
SMCR7
SMG7
SMTNL2
SMYD4
SNAP91
SNF1LK
SNHG6
SNRPA1
SNRPD1
SNRPF
SNX1
SNX17
SNX20
SNX6
SOCS2
SOD2
SOX17
SOX21
SPA17
SPARC
SPATA17
SPATS1
SPDEF
SPG21
SPG3A
SPHK1
SPIB
SPIC
SPIN4
SPINT1
SPINT2
SPNS3
SPP1
SPRYD4
SRC
SRGAP2
SRP14
SRR
SRRM1
SRXN1
SSB
SSBP4
SSNA1
ST3GAL1
CMBL
CMKLR1
CML2
CMTM3
CMTM4
CMTM7
CMTM8
CNBP
CNDP1
CNDP2
CNGA3
CNIH3
CNIH4
CNKSR1
CNKSR2
CNKSR3
CNN3
CNNM2
CNNM4
CNOT1
CNOT10
CNOT2
CNOT4
CNOT6
CNOT8
CNPY1
CNPY2
CNPY3
CNR1
CNR2
CNST
CNTFR
CNTN1
CNTN2
CNTN3
CNTN4
CNTN5
CNTNAP2
CNTNAP5C
CNTROB
COBL
COBLL1
COG1
COG3
COG5
COG8
COIL
COL11A2
COL12A1
COL13A1
COL14A1
COL15A1
COL16A1

ST8SIA1
STAC3
STAG3
STAMBPL1
STARD4
STARD6
STARD9
STAT1
STAT2
STAT3
STIP1
STK22S1
STK3
STK36
STK38
STMN3
STOML2
STRA8
STT3A
STXBP5L
SUCLA2
SULT5A1
SULT6B1
SUMO1
SUOX
SUSD2
SUV420H2
SYCE2
SYNGR2
SYNGR4
SYNPO2L
SYT5
TACC1
TACC3
TAF13
TAF7
TAGLN2
TALDO1
TARS
TARS2
TAS2R138
TBC1D8
TBCA
TBL2
TBX3
TCEA1
TCEAL7
TCF19
TCFAP2A
TCFAP2C
TCFCP2L1
TCFEB
TCL1

COL18A1
COL19A1
COL1A1
COL23A1
COL25A1
COL28A1
COL4A3BP
COL5A1
COL5A3
COL6A1
COL6A2
COL9A1
COL9A2
COLEC11
COLEC12
COMMD1
COMMD4
COMMD5
COMMD7
COMP
COMT1
COPA
COPB1
COPG
COPS3
COPS4
COPS7A
COPS7B
COPS8
COQ10A
COQ10B
COQ5
COQ7
CORIN
CORO1A
CORO1B
CORO1C
CORO2B
CORO6
COTL1
COX10
COX11
COX15
COX4I1
COX4I2
COX5B
COX6A1
COX6B2
COX7A1
COX7A2
COX7A2L
COX7C
COX8C

TCP11
TCTEX1D2
TDH
TEAD2
TEAD4
TEF
TEKT1
TEKT2
TERF2
TERT
TESC
TET2
TFF3
TFPI
TFRC
TGFBI
TGM1
TGM2
THNSL1
THOP1
THRAP3
THRSP
THTPA
TICAM1
TICAM2
TIMM44
TIMP1
TIPARP
TISP22
TJAP1
TJP3
TK1
TLE6
TLL1
TM4SF5
TM7SF2
TM7SF3
TMBIM1
TMCO4
TMCO6
TMED6
TMEM102
TMEM106A
TMEM117
TMEM120A
TMEM126B
TMEM136
TMEM141
TMEM14C
TMEM161B
TMEM166
TMEM17
TMEM179
CPA2
CPA4
CPA6
CPD
CPE
CPEB1
CPEB3
CPEB4
CPLX1
CPLX2
CPLX4
CPM
CPN1
CPN2
CPNE2
CPNE4
CPNE5
CPNE8
CPNE9
CPSF4L
CPSF6
CPT1A
CPT1B
CPVL
CPZ
CR1L
CR2
CRABP1
CRADD
CRB2
CRBN
CREB1
CREB3
CREB3L1
CREB3L2
CREB5
CREBBP
CREBL2
CREG1
CRELD2
CRHBP
CRIP1
CRIP2
CRIP3
CRIPT
CRISPLD1
CRISPLD2
CRKRS
CRLF1
CRMP1
CROCC
CRP
CRTAC1

TMEM19	CRTAP
TMEM191C	CRX
TMEM2	CRXOS1
TMEM205	CRY2
TMEM37	CRYAA
TMEM39A	CRYBA1
TMEM51	CRYBA2
TMEM62	CRYBB3
TMEM63C	CRYBG3
TMEM64	CRYGN
TMEM66	CRYGS
TMEM8	CRYL1
TMEM81	CRYZL1
TMEM82	CSAD
TMPRSS5	CSDE1
TMSB4X	CSF1
TMTC4	CSF1R
TMUB1	CSF2
TNIP1	CSF3R
TNK1	CSGALNACT1
TNNC1	CSGALNACT2
TNRC18	CSK
TOB1	CSMD1
TOM1	CSMD3
TOMM7	CSNK1A1
TPD52L1	CSNK1E
TPI-RS4	CSNK1G3
TPMT	CSNK2A1
TPPP3	CSNK2A2
TPST2	CSPG4
TPT1	CSPG5
TRAF3IP2	CSPP1
TRAIP	CSRNP1
TRAM2	CSRNP2
TRAPPC5	CSRNP3
TRERF1	CSRP1
TREX1	CSRP2
TRF	CSRP2BP
TRIAP1	CST3
TRIB3	CST6
TRIM13	CST7
TRIM16	CSTAD
TRIM25	CSTB
TRIM36	CSTF1
TRIM47	CSTF2
TRIM54	CSTF3
TRIM55	CSTL1
TRIM6	CTBP1
TRIM63	CTBP2
TRIM8	CTBS
TRIML1	CTCF
TRIML2	CTDP1
TRP53I13	CTDSP2

TRPD52L3
TRPM7
TSEN34
TSFM
TSGA14
TSPAN1
TSPAN32
TSPO
TTC19
TTC25
TTC28
TTC35
TTC4
TTC7
TTC9
TTF1
TTLL13
TTLL6
TUBA3A
TUBB2A
TUBB2B
TUBB3
TUBD1
TUBG2
TUG1
TULP2
TULP4
TUSC1
TXN1
TXNDC14
TXNIP
TYSND1
UAP1L1
UBA1
UBE2D3
UBE2M
UBE2W
UBE4A
UBN1
UBQLN4
UBTF
UBXD5
UCHL4
UCHL5IP
UCKL1
UFC1
UHRF1
UNC13A
UNC13B
UNC13D
UNC84B
UPB1
UPF1

CTDSPL
CTF1
CTGF
CTH
CTHRC1
CTLA4
CTNNA1
CTNNA2
CTNNA3
CTNNAL1
CTNNB1
CTNNBIP1
CTNNBL1
CTNND1
CTNND2
CTR9
CTRC
CTSA
CTSB
CTSC
CTSD
CTSLL3
CTTN
CTTNBP2
CTTNBP2NL
CTXN1
CTXN2
CTXN3
CUBN
CUEDC1
CUEDC2
CUGBP2
CUL1
CUL2
CUL4A
CUL7
CUTA
CUX1
CUX2
CWC15
CWH43
CX3CL1
CXADR
CXCL12
CXCL14
CXCL16
CXCL17
CXCR3
CXCR4
CXCR5
CXCR7
CXXC5
CYB5

UPP1	CYB561
UQCRB	CYB561D1
UQCRC1	CYB5B
USE1	CYB5R1
USP11	CYB5R2
USP3	CYB5R3
USP48	CYBB
USP49	CYBRD1
UXS1	CYCS
V1RB7	CYFIP1
V1RH7	CYFIP2
VAMP2	CYGB
VASH1	CYHR1
VASP	CYLC1
VAT1	CYP11A1
VAX2	CYP19A1
VCAM1	CYP26A1
VDAC3	CYP26B1
VEGFA	CYP27A1
VEGFC	CYP2A12
VIM	CYP2AB1
VMN2R10	CYP2B10
VPREB1	CYP2C37
VPREB3	CYP2C55
VPS11	CYP2C69
VPS13C	CYP2C70
VPS18	CYP2D12
VPS25	CYP2D22
VPS39	CYP2D9
VRK1	CYP2F2
VSIG2	CYP2J9
VSTM2L	CYP2S1
VWF	CYP2T4
WBP1	CYP2U1
WBP5	CYP3A13
WBSCR16	CYP4A10
WDR12	CYP4A31
WDR16	CYP4F13
WDR31	CYP4F16
WDR34	CYP4F18
WDR42A	CYP4F39
WDR5	CYP4X1
WDR51A	CYP8B1
WDR51B	CYPT12
WDR57	CYR61
WDR5B	CYS1
WDR78	CYTH1
WDR90	CYTH2
WFDC15A	CYTH3
WFDC2	CYTH4
WIPI1	CYTL1
WIPI2	CYTSA
WNT10A	CYTSB

WNT11	D030018L15RIK
WNT3A	D030028A08RIK
WNT6	D030074E01RIK
WSCD1	D0H4S114
WSCD2	D10627
XKRX	D10BWG1379E
XPNPEP1	D10ERTD322E
XPNPEP2	D10ERTD610E
XRCC5	D10JHU81E
YES1	D10WSU102E
YIF1A	D10WSU52E
YPEL2	D11BWG0517E
YWHAE	D130009I18RIK
ZADH1	D130040H23RIK
ZAP70	D130043K22RIK
ZBTB12	D14ABB1E
ZBTB3	D14ERTD436E
ZBTB32	D14ERTD668E
ZBTB44	D15ERTD621E
ZBTB45	D15WSU169E
ZC3H7A	D16ERTD472E
ZC3HAV1	D16H22S680E
ZCCHC11	D17WSU92E
ZCCHC4	D18ERTD653E
ZCCHC6	D19ERTD386E
ZCWPW1	D19WSU162E
ZDHHC14	D1BWG0212E
ZDHHC17	D1PAS1
ZDHHC3	D230025D16RIK
ZDHHC4	D230037D09RIK
ZEB1	D2ERTD391E
ZFAND1	D330028D13RIK
ZFHX2	D330038O06RIK
ZFP1	D3BWG0562E
ZFP106	D430019H16RIK
ZFP286	D430020J02RIK
ZFP295	D430041D05RIK
ZFP367	D430042O09RIK
ZFP36L1	D4BWG0951E
ZFP383	D4ERTD22E
ZFP414	D4WSU53E
ZFP42	D5ERTD579E
ZFP459	D630003M21RIK
ZFP516	D630029K05RIK
ZFP518B	D630032N06RIK
ZFP57	D630037F22RIK
ZFP639	D630039A03RIK
ZFP640	D630042P16RIK
ZFP650	D630045J12RIK
ZFP704	D6ERTD474E
ZFP750	D6WSU116E
ZFP78	D6WSU163E
ZFP811	D730001G18RIK

ZFP819
ZFP87
ZFP97
ZHX2
ZKSCAN2
ZMAT2
ZMAT4
ZMYM3
ZMYM6
ZMYND15
ZNHIT4
ZNRD1
ZRANB3

D730039F16RIK
D730040F13RIK
D7ERTD443E
D8ERTD82E
D930014E17RIK
D930015E06RIK
D930020B18RIK
D930048N14RIK
D9ERTD402E
DAAM1
DAB1
DAB2
DAB2IP
DACH1
DACT1
DACT2
DACT3
DAD1
DAG1
DAGLA
DAGLB
DAND5
DAO
DAP
DAP3
DAPK1
DAPL1
DAPP1
DARS
DAXX
DAZAP1
DAZAP2
DAZL
DBI
DBNL
DBP
DBPHT2
DBR1
DBX1
DCAF11
DCAF12
DCAF4
DCAF6
DCAF7
DCAF8
DCAKD
DCBLD1
DCC
DCDC2A
DCI
DCK
DCLK1
DCLK2

DCLK3
DCLRE1A
DCP1A
DCP1B
DCPS
DCST1
DCT
DCTD
DCTN1
DCTN2
DCTN4
DCTN6
DCUN1D3
DDAH1
DDAH2
DDB1
DDB2
DDC
DDHD1
DDHD2
DDIT4
DDN
DDOST
DDR1
DDRGK1
DDT
DDX10
DDX11
DDX20
DDX26B
DDX28
DDX3X
DDX3Y
DDX46
DDX49
DDX5
DDX54
DDX58
DDX59
DDX6
DEAF1
DEAR1
DEDD
DEDD2
DEF6
DEFA-PS13
DEFB1
DEFB11
DEFB13
DEFB15
DEFB40
DEFB42
DEFB7

DEFB8
DEGS2
DEK
DEM1
DENND1A
DENND1B
DENND2A
DENND2C
DENND3
DENND4A
DENND4B
DENND5A
DENND5B
DEPDC1B
DEPDC6
DEPDC7
DERA
DERL3
DES
DFFB
DGAT2
DGCR2
DGCR8
DGKA
DGKE
DGKG
DGKH
DGKI
DGKZ
DGUOK
DHCR24
DHCR7
DHDDS
DHDH
DHH
DHRS3
DHRS4
DHRS7
DHRS7C
DHTKD1
DHX16
DHX30
DHX32
DHX34
DHX35
DHX37
DHX40
DHX57
DHX58
DHX8
DHX9
DIAP1
DIAP2

DIAP3
DICER1
DIDO1
DIMT1
DIO3
DIO3OS
DIP2A
DIP2B
DIP2C
DIRAS2
DIRC2
DIS3L
DIS3L2
DISC1
DIXDC1
DKK3
DKKL1
DLC1
DLEC1
DLEU2
DLEU7
DLG1
DLG3
DLG5
DLGAP1
DLGAP3
DLGAP4
DLGAP5
DLK1
DLK2
DLL1
DLL4
DLST
DLX1AS
DLX2
DLX3
DLX4
DMBX1
DMD
DMGDH
DMRT1
DMRT2
DMRT3
DMRTA2
DMRTB1
DMRTC2
DMXL2
DNA2
DNAHC1
DNAHC10
DNAHC11
DNAHC2
DNAHC6

DNAHC8
DNAIC1
DNAIC2
DNAJA2
DNAJA3
DNAJB1
DNAJB11
DNAJB12
DNAJB13
DNAJB2
DNAJB3
DNAJB6
DNAJB8
DNAJC1
DNAJC10
DNAJC11
DNAJC12
DNAJC13
DNAJC15
DNAJC16
DNAJC18
DNAJC19
DNAJC21
DNAJC22
DNAJC25
DNAJC27
DNAJC3
DNAJC5
DNAJC5B
DNAJC6
DNAJC7
DNAJC8
DNALC4
DNASE1
DNASE1L3
DNER
DNLZ
DNM1
DNM1L
DNM2
DNM3
DNMBP
DNMT3A
DNMT3B
DNMT3L
DNPEP
DNTTIP2
DOC2B
DOCK1
DOCK10
DOCK2
DOCK4
DOCK5

DOCK6
DOCK7
DOCK8
DOCK9
DOHH
DOK2
DOK3
DOK5
DOPEY2
DOT1L
DPAGT1
DPCR1
DPEP2
DPF1
DPF3
DPH3
DPM1
DPP6
DPPA3
DPPA4
DPPA5A
DPT
DPY19L3
DPY30
DPYD
DPYS
DPYSL3
DPYSL5
DQX1
DR1
DRAM2
DRD4
DSC1
DSC2
DSCAM
DSCAML1
DSCR3
DSE
DSEL
DSG1C
DSG2
DSG4
DSN1
DSP
DSPP
DST
DSTN
DSTYK
DTD1
DTL
DTNA
DTNB
DTNBP1

DTWD1
DTX1
DTX2
DTX4
DUOX1
DUS2L
DUS3L
DUSP1
DUSP10
DUSP11
DUSP12
DUSP16
DUSP2
DUSP23
DUSP26
DUSP27
DUSP3
DUSP4
DUSP5
DUSP6
DUSP7
DUSP9
DUT
DVL1
DVL3
DVWA
DYM
DYNC1H1
DYNC1I1
DYNC1I2
DYNC1LI1
DYNC1LI2
DYNC2LI1
DYNLL1
DYNLL2
DYNLRB2
DYNLT1A
DYNLT1B
DYRK1A
DYRK1B
DYRK2
DYRK3
DYSF
DZIP1
E030003E18RIK
E030010A14RIK
E030011O05RIK
E030025P04RIK
E130012A19RIK
E130306D19RIK
E130309D02RIK
E130309D14RIK
E130309F12RIK

E130311K13RIK
E130317F20RIK
E230019M04RIK
E230025N22RIK
E230029C05RIK
E2F1
E2F2
E2F3
E2F6
E2F7
E330016A19RIK
E330026B02RIK
E330033B04RIK
E330034G19RIK
EAPP
EBAG9
EBF1
EBF2
EBF3
EBF4
EBPL
ECD
ECE1
ECHDC2
ECHDC3
ECM1
ECT2
EDA
EDA2R
EDAR
EDARADD
EDEM1
EDIL3
EDN1
EDN2
EDN3
EEA1
EED
EEF1A1
EEF1E1
EEF1G
EEF2
EEF2K
EEFSEC
EEPD1
EFCAB10
EFCAB2
EFCAB4B
EFCAB5
EFCAB6
EFEMP1
EFHA1
EFHC1

EFNA1
EFNA2
EFNA3
EFNA4
EFNA5
EFNB1
EFNB2
EFNB3
EFTUD1
EG240055
EGFL7
EGFLAM
EGFR
EGLN3
EGR1
EGR2
EGR4
EHD1
EHD2
EHD3
EHD4
EHF
EHHADH
EHMT1
EI24
EID2
EID3
EIF1
EIF1A
EIF1B
EIF2A
EIF2AK2
EIF2AK3
EIF2B2
EIF2B3
EIF2B5
EIF2C1
EIF2C2
EIF2C3
EIF2S2
EIF2S3X
EIF3B
EIF3C
EIF3E
EIF3H
EIF3I
EIF3K
EIF3L
EIF4A2
EIF4A3
EIF4B
EIF4E
EIF4E2

EIF4E3
EIF4EBP1
EIF4EBP2
EIF4G1
EIF4G3
EIF5
EIF5A
EIF5A2
ELAC1
ELAC2
ELAVL1
ELAVL2
ELF1
ELF2
ELF3
ELF5
ELFN1
ELFN2
ELK1
ELK3
ELK4
ELL
ELL2
ELL3
ELMO1
ELMO2
ELMO3
ELMOD1
ELMOD2
ELN
ELOVL2
ELOVL5
ELOVL6
ELOVL7
ELP3
ELP4
EMB
EMD
EMID1
EMID2
EMILIN1
EMILIN2
EML1
EML2
EML4
EML6
EMP1
EMP2
EMR1
EMX1
EN1
EN2
ENAH

ENC1
ENDOD1
ENG
ENHO
ENO1
ENOPH1
ENOX1
ENOX2
ENPEP
ENPP3
ENPP6
ENSA
ENTHD1
ENTPD1
ENTPD2
ENTPD4
ENTPD6
ENTPD7
ENY2
EOMES
EP300
EP400
EPAS1
EPB4.1
EPB4.1L1
EPB4.1L2
EPB4.1L3
EPB4.1L4A
EPB4.1L4B
EPB4.9
EPC2
EPCAM
EPHA10
EPHA2
EPHA4
EPHA5
EPHB1
EPHB2
EPHB3
EPHB4
EPHX1
EPHX3
EPM2A
EPM2AIP1
EPN1
EPN2
EPN3
EPPK1
EPS15
EPS15L1
EPS8
EPS8L2
EPS8L3

EPSTI1
ERAP1
ERAS
ERBB2IP
ERBB3
ERBB4
ERC1
ERC2
ERCC1
ERCC3
ERCC4
ERCC6
ERCC6L
ERF
ERG
ERGIC1
ERI3
ERICH1
ERLEC1
ERLIN1
ERMP1
ERN1
ERO1L
ERP27
ERP44
ERRFI1
ERV3
ES1
ES31
ESCO1
ESPL1
ESPN
ESR2
ESRP1
ESRP2
ESRRA
ESRRB
ESYT2
ESYT3
ETAA1
ETF1
ETFA
ETFB
ETHE1
ETL4
ETNK1
ETS1
ETS2
ETV1
ETV2
ETV3
ETV4
ETV5

ETV6
EVC
EVI5
EVI5L
EVL
EVPL
EVX1
EWSR1
EXD2
EXOC1
EXOC2
EXOC3L
EXOC4
EXOC6
EXOC6B
EXOC7
EXOC8
EXOG
EXOSC10
EXOSC3
EXPH5
EXT1
EXT2
EXTL1
EXTL3
EYA2
EYA3
EYA4
EZH2
EZR
F10
F11
F11R
F2
F2R
F2RL1
F2RL2
F3
F730016J06RIK
F730043M19RIK
F730047E07RIK
F8
F830045P16RIK
F830116E18RIK
FA2H
FAAH
FABP1
FABP12
FABP3
FABP6
FADD
FADS1
FADS2

FAF1
FAF2
FAHD2A
FAIM2
FAIM3
FAM100B
FAM101A
FAM101B
FAM102A
FAM102B
FAM103A1
FAM105A
FAM105B
FAM107A
FAM107B
FAM108A
FAM108B
FAM109A
FAM110B
FAM110C
FAM111A
FAM113B
FAM114A1
FAM114A2
FAM115A
FAM115C
FAM116A
FAM117A
FAM117B
FAM118A
FAM118B
FAM119A
FAM120A
FAM120B
FAM120C
FAM122B
FAM123A
FAM123B
FAM123C
FAM124B
FAM125B
FAM126A
FAM128B
FAM129A
FAM129B
FAM129C
FAM131B
FAM131C
FAM133B
FAM134B
FAM134C
FAM135A
FAM135B

FAM136A
FAM13A
FAM13B
FAM13C
FAM149B
FAM155A
FAM159A
FAM159B
FAM160B1
FAM161A
FAM162A
FAM163A
FAM167A
FAM167B
FAM168A
FAM168B
FAM169A
FAM169B
FAM170B
FAM172A
FAM175A
FAM175B
FAM176A
FAM176B
FAM178A
FAM178B
FAM180A
FAM184A
FAM186B
FAM188B
FAM189A1
FAM189A2
FAM189B
FAM18A
FAM18B
FAM190A
FAM192A
FAM193A
FAM195A
FAM195B
FAM196B
FAM198B
FAM19A1
FAM19A2
FAM19A3
FAM19A4
FAM19A5
FAM20A
FAM20B
FAM20C
FAM26E
FAM26F
FAM32A

FAM35A
FAM36A
FAM38A
FAM38B
FAM3B
FAM3C
FAM40A
FAM40B
FAM43A
FAM43B
FAM46B
FAM46C
FAM49A
FAM50B
FAM53A
FAM53B
FAM55C
FAM55D
FAM57A
FAM58B
FAM59A
FAM5C
FAM60A
FAM63A
FAM65A
FAM65C
FAM69A
FAM69B
FAM69C
FAM71A
FAM71E1
FAM71E2
FAM71F1
FAM73A
FAM76A
FAM76B
FAM78A
FAM78B
FAM81A
FAM82A1
FAM82B
FAM83A
FAM83D
FAM84A
FAM86
FAM92B
FAM96A
FAM98B
FANCA
FANCC
FANCE
FANCG
FANCI

FANCL
FAR1
FARP1
FARS2
FARSA
FAS
FASL
FASN
FASTK
FAT1
FAT2
FAT4
FBF1
FBLIM1
FBLN1
FBLN2
FBLN5
FBN2
FBP1
FBP2
FBRS
FBRSL1
FBXL12
FBXL13
FBXL15
FBXL16
FBXL17
FBXL18
FBXL19
FBXL2
FBXL20
FBXL22
FBXL3
FBXL4
FBXL5
FBXL6
FBXL7
FBXO10
FBXO11
FBXO15
FBXO16
FBXO17
FBXO2
FBXO21
FBXO22
FBXO25
FBXO27
FBXO28
FBXO3
FBXO30
FBXO31
FBXO32
FBXO33

FBXO36
FBXO41
FBXO42
FBXO43
FBXO47
FBXO6
FBXO8
FBXO9
FBXW10
FBXW11
FBXW13
FBXW15
FBXW17
FBXW20
FBXW4
FBXW5
FBXW7
FBXW8
FCF1
FCGBP
FCGR1
FCGR3
FCGR4
FCGRT
FCHO1
FCHO2
FCHSD2
FCRL1
FCRL5
FCRLB
FCRLS
FDFT1
FDX1
FDX1L
FEM1A
FEM1B
FEM1C
FEN1
FERMT2
FERMT3
FERT2
FETUB
FEV
FEZ1
FEZ2
FEZF2
FFAR2
FFAR3
FGD1
FGD2
FGD4
FGD5
FGD6

FGF1
FGF10
FGF12
FGF14
FGF15
FGF17
FGF21
FGF23
FGF3
FGF4
FGF5
FGF6
FGF8
FGF9
FGFR1
FGFR1OP
FGFR2
FGFR3
FGFR4
FGG
FGGY
FGL1
FGR
FH1
FHAD1
FHDC1
FHIT
FHL2
FHL3
FHL4
FHOD1
FHOD3
FICD
FIG4
FIGNL1
FILIP1
FILIP1L
FITM2
FJX1
FKBP11
FKBP3
FKBP5
FKBP6
FKBP8
FKBP9
FLCN
FLI1
FLNA
FLNB
FLNC
FLOT2
FLRT1
FLRT2

FLRT3
FLT1
FLT3
FLT4
FLYWCH1
FLYWCH2
FMC1
FMN1
FMN2
FMNL1
FMNL2
FMNL3
FMO1
FMOD
FN1
FN3KRP
FNBP1
FNBP1L
FNBP4
FNDC1
FNDC3A
FNDC3B
FNDC5
FNTB
FOLH1
FOS
FOSB
FOSL2
FOXA1
FOXA2
FOXA3
FOXB1
FOXB2
FOXC1
FOXD1
FOXD3
FOXE1
FOXF1A
FOXH1
FOXI1
FOXI3
FOXJ1
FOXJ2
FOXJ3
FOXK1
FOXK2
FOXL1
FOXM1
FOXN2
FOXN3
FOXN4
FOXO1
FOXO3

FOXO6
FOXP1
FOXP2
FOXP4
FOXQ1
FRAS1
FRAT1
FREM1
FREM2
FREQ
FRMD3
FRMD4A
FRMD4B
FRMD5
FRMD6
FRMD8
FRMPD1
FRMPD3
FRMPD4
FRRS1
FRS2
FRS3
FRY
FRYL
FRZB
FSCB
FSCN1
FSCN2
FSD2
FSHB
FSIP1
FSTL1
FSTL4
FSTL5
FTCD
FTH1
FTL1
FTMT
FTO
FTSJ1
FUBP1
FUBP3
FUCA1
FUCA2
FUNDC1
FURIN
FUT10
FUT11
FUT2
FUT7
FUT9
FXN
FXR1

FXYD2
FXYD3
FXYD4
FXYD5
FXYD6
FYB
FYN
FYTTD1
FZD10
FZD2
FZD3
FZD4
FZD5
FZD6
FZD7
FZD8
FZD9
FZR1
G0S2
G2E3
G3BP1
G3BP2
G630016D24RIK
G630025P09RIK
G6B
GAA
GAB1
GABARAPL1
GABARAPL2
GABBR1
GABBR2
GABPA
GABPB1
GABPB2
GABRA5
GABRB2
GABRB3
GABRG3
GABRP
GABRR1
GABRR2
GABRR3
GAD1
GAD2
GADD45A
GADD45B
GADD45G
GADL1
GAL
GAL3ST3
GAL3ST4
GALE
GALK2

GALM
GALNT10
GALNT12
GALNT13
GALNT2
GALNT3
GALNT4
GALNT6
GALNT7
GALNT9
GALNTL1
GALNTL2
GALNTL4
GALNTL5
GALP
GALR2
GALT
GAMT
GAN
GANAB
GAP43
GAPDH
GAPVD1
GARNL3
GARS
GART
GAS1
GAS2L3
GAS5
GAS6
GAS7
GATA2
GATA3
GATA5
GATA6
GATAD2A
GATAD2B
GATSL3
GBA
GBAS
GBE1
GBGT1
GBP2
GBP5
GBP6
GBX1
GBX2
GCA
GCAP14
GCAT
GCDH
GCGR
GCH1

GCK
GCLC
GCLM
GCM1
GCM2
GCN1L1
GCNT1
GCNT2
GCNT3
GCNT4
GCOM1
GDA
GDAP1
GDAP1L1
GDAP2
GDE1
GDF15
GDF3
GDF6
GDI2
GDPD3
GDPD5
GEM
GEN1
GFI1
GFI1B
GFM1
GFM2
GFOD1
GFOD2
GFPT1
GFPT2
GFRA1
GFRA2
GFRA3
GFRA4
GGA1
GGNBP1
GGNBP2
GGT5
GGT6
GGT7
GGTA1
GHDC
GHR
GHRHR
GHSR
GIMAP8
GINS2
GINS3
GIP
GIPC1
GIPC2

GIT1
GIT2
GIYD2
GJA1
GJA3
GJA5
GJB1
GJB3
GJB4
GJB5
GJB6
GJC1
GJC2
GJD3
GJD4
GJE1
GK5
GKAP1
GLB1
GLCCI1
GLCE
GLDC
GLDN
GLG1
GLI1
GLI2
GLIPR2
GLIS1
GLIS2
GLIS3
GLO1
GLOD5
GLP1R
GLRA4
GLRP1
GLRX
GLRX2
GLRX3
GLRX5
GLS
GLT1D1
GLT25D1
GLT25D2
GLT28D2
GLT8D2
GLT8D3
GLTP
GLTPD1
GLTPD2
GLTSCR1
GLUD1
GLYCAM1
GLYCTK

GLYR1
GM10035
GM10069
GM1027
GM10451
GM10565
GM10778
GM10863
GM10941
GM11194
GM11330
GM11346
GM11437
GM11487
GM11541
GM11545
GM11744
GM11961
GM11978
GM12166
GM12169
GM12528
GM12789
GM12824
GM12888
GM13128
GM13152
GM13154
GM1322
GM13242
GM13251
GM13280
GM13363
GM1337
GM13547
GM13646
GM13749
GM13889
GM14005
GM14092
GM14203
GM14207
GM15217
GM1527
GM1564
GM1568
GM1574
GM1587
GM1631
GM16386
GM16432
GM16515
GM16517

GM16532
GM1943
GM1968
GM216
GM2176
GM239
GM266
GM3219
GM347
GM414
GM4371
GM438
GM4769
GM4792
GM4850
GM4894
GM4922
GM4925
GM4931
GM4934
GM4937
GM505
GM5077
GM5089
GM5105
GM5113
GM5124
GM5127
GM5129
GM5132
GM5134
GM5148
GM5158
GM52
GM525
GM5424
GM5434
GM5447
GM5468
GM5475
GM5506
GM5512
GM5531
GM5544
GM5567
GM5607
GM5627
GM572
GM5766
GM5801
GM5820
GM5887
GM595

GM5972
GM608
GM6251
GM628
GM648
GM6524
GM6578
GM6644
GM6710
GM672
GM6907
GM694
GM6981
GM7092
GM71
GM7120
GM7244
GM7265
GM7334
GM7348
GM7367
GM7714
GM7904
GM806
GM815
GM826
GM829
GM833
GM839
GM8439
GM853
GM8580
GM8615
GM8783
GM8801
GM8817
GM884
GM9376
GM941
GM949
GM962
GM973
GM9733
GM98
GM9904
GMDS
GMEB1
GMFB
GMFG
GMPPA
GMPPB
GMPR
GMPS

GNA11
GNA12
GNA13
GNA14
GNA15
GNAI1
GNAI2
GNAI3
GNAL
GNAL1
GNAO1
GNAQ
GNAS
GNAT2
GNB1
GNB1L
GNB2
GNB2L1
GNB4
GNB5
GNE
GNG10
GNG13
GNG2
GNG3
GNG4
GNG7
GNGT2
GNPDA1
GNPNAT1
GNRH1
GNRHR
GNS
GOLGA4
GOLGA7
GOLGA7B
GOLM1
GOLPH3
GOPC
GORAB
GORASP2
GOSR1
GOT1
GOT2
GP5
GPA33
GPAT2
GPATCH2
GPATCH3
GPATCH8
GPBP1
GPC1
GPC3

GPC4
GPC5
GPC6
GPD1L
GPD2
GPHB5
GPHN
GPI1
GPN2
GPN3
GPNMB
GPR1
GPR107
GPR114
GPR116
GPR12
GPR120
GPR123
GPR124
GPR125
GPR132
GPR133
GPR135
GPR142
GPR150
GPR151
GPR155
GPR156
GPR157
GPR158
GPR160
GPR161
GPR176
GPR177
GPR179
GPR180
GPR19
GPR20
GPR26
GPR31C
GPR37L1
GPR39
GPR4
GPR45
GPR50
GPR55
GPR56
GPR6
GPR62
GPR64
GPR68
GPR75
GPR77

GPR88
GPR97
GPR98
GPRC2A-RS5
GPRC5A
GPRC5B
GPRC5C
GPRC5D
GPRIN2
GPRIN3
GPS2
GPSM1
GPT2
GPX1
GPX4
GPX5
GPX7
GRAMD1A
GRAMD1B
GRAMD3
GRAMD4
GRAP
GRAP2
GRASP
GRB10
GRB14
GRB2
GRB7
GREB1
GREB1L
GRHL1
GRHL2
GRHL3
GRHPR
GRIA2
GRIA4
GRID1
GRID2
GRIFIN
GRIK2
GRIK3
GRIK4
GRIK5
GRIN1
GRIN2A
GRIN2B
GRIN3B
GRINA
GRINL1A
GRIP1
GRIPAP1
GRIT
GRK4

GRK5
GRM4
GRM5
GRM8
GRN
GRP
GRPEL1
GRRP1
GRSF1
GRTP1
GSC
GSC2
GSDMC
GSDMD
GSE1
GSG1L
GSK3B
GSN
GSPT1
GSR
GSS
GSTA2
GSTA3
GSTA4
GSTM1
GSTM3
GSTM6
GSTM7
GSTO1
GSTP1
GSTT2
GSX1
GSX2
GTF2A1
GTF2E1
GTF2F2
GTF2H1
GTF2H4
GTF2I
GTF2IRD1
GTF2IRD2
GTF3A
GTF3C5
GTF3C6
GTPBP4
GTPBP6
GTPBP8
GT(ROSA)26SOR
GTSE1
GTSF1
GTSF1L
GUCA1B
GUCA2A

GUCY1B2
GUCY2D
GUCY2E
GUCY2G
GUF1
GUK1
GULP1
GUSB
GYLTL1B
GYPC
GYS1
GYS2
GZF1
GZMB
H13
H1F0
H2-AB1
H2AFX
H2AFY
H2AFY2
H2AFZ
H2-BL
H2-DMA
H2-M5
H2-OA
H2-Q10
H3F3A
H6PD
HAAO
HABP2
HABP4
HACE1
HACL1
HADH
HADHB
HAGH
HAL
HAND1
HAND2
HAP1
HAPLN4
HARS2
HAS1
HAS3
HAT1
HAUS1
HAUS4
HAUS8
HAVCR2
HBA-A1
HBEGF
HBP1
HBQ1

HBS1L
HBXIP
HCCS
HCFC1
HCFC2
HCK
HCLS1
HCN1
HCN4
HCRTR1
HCRTR2
HDAC1
HDAC4
HDAC5
HDAC6
HDAC7
HDAC8
HDGF
HDGFL1
HDLBP
HEATR1
HEATR2
HEATR5A
HEATR5B
HEATR6
HEBP1
HEBP2
HECTD1
HECTD2
HECW1
HECW2
HEG1
HELB
HELLS
HELQ
HELT
HELZ
HEMGN
HEPACAM
HERC1
HERC3
HERC4
HERPUD1
HERPUD2
HES1
HES3
HES5
HES6
HEXA
HEXB
HEXDC
HEXIM1
HFE2

HFM1
HGSNAT
HHAT
HHATL
HHEX
HHIPL1
HIAT1
HIBADH
HIC1
HIC2
HIF1AN
HIF3A
HIGD1A
HILS1
HINT3
HIP1
HIP1R
HIPK1
HIPK2
HIPK3
HIRA
HIST1H1A
HIST1H1C
HIST1H2AD
HIST1H2AH
HIST1H2AI
HIST1H2BF
HIST1H2BP
HIST1H3D
HIST1H3E
HIST1H3H
HIST1H4H
HIST2H2AA1
HIST2H2BE
HIST2H4
HIST3H2BA
HIVEP1
HIVEP2
HIVEP3
HK1
HK2
HKDC1
HLCS
HLTF
HLX
HMBS
HMCN1
HMG20A
HMG20B
HMGA1
HMGA2
HMGB1
HMGB2

HMGB4
HMGCL
HMGCR
HMGN1
HMGN2
HMGN3
HMGN5
HMGXB3
HMGXB4
HMHA1
HMOX1
HMX1
HMX3
HN1L
HNF1A
HNF1B
HNMT
HNRNPA0
HNRNPA2B1
HNRNPA3
HNRNPD
HNRNPF
HNRNPH2
HNRNPK
HNRNPL
HNRNPR
HNRPDL
HNRPLL
HOMER1
HOMER2
HOMER3
HOMEZ
HOOK2
HORMAD2
HOXA1
HOXA10
HOXA11
HOXA13
HOXA3
HOXA7
HOXA9
HOXB13
HOXB3
HOXB9
HOXC12
HOXC13
HOXC4
HOXC5
HOXC8
HOXD1
HOXD12
HOXD3
HOXD4

HOXD8
HOXD9
HPCA
HPCAL1
HPCAL4
HPD
HPDL
HPGD
HPGDS
HPN
HPS3
HPS5
HPS6
HPSE
HPSE2
HPX
HRH1
HRH2
HRH3
HRNR
HRSP12
HS1BP3
HS2ST1
HS3ST2
HS3ST3B1
HS3ST5
HS3ST6
HS6ST1
HS6ST3
HSBP1
HSBP1L1
HSD11B1
HSD17B11
HSD17B12
HSD17B14
HSD17B2
HSD17B3
HSD17B6
HSD17B7
HSDL2
HSF1
HSF2
HSF2BP
HSH2D
HSP90AA1
HSP90AB1
HSPA14
HSPA1A
HSPA1B
HSPA1L
HSPA4
HSPA5
HSPA8

HSPA9
HSPB1
HSPB11
HSPB3
HSPB7
HSPB8
HSPB9
HSPBAP1
HSPE1
HSPG2
HTATIP2
HTR1B
HTR1D
HTR2B
HTR2C
HTR4
HTR5A
HTR7
HTRA1
HTRA3
HTRA4
HTT
HUNK
HUWE1
HVCN1
HYAL1
HYAL3
HYAL4
HYAL5
HYAL6
HYDIN
HYI
I1C0022H11RIK
IAH1
IARS2
IBTK
ICA1
ICAM1
ICAM4
ICAM5
ICK
ICMT
ICOSL
ICT1
ID1
ID2
ID3
ID4
IDE
IDH2
IDH3A
IDO2
IDS

IER2
IER3
IER5
IER5L
IFFO1
IFFO2
IFI202B
IFITM1
IFITM2
IFITM3
IFITM6
IFLTD1
IFNA13
IFNAR1
IFNAR2
IFNG
IFNGR2
IFRD1
IFT122
IFT52
IFT80
IFT81
IGDCC3
IGDCC4
IGF1
IGF1R
IGF2BP1
IGF2BP2
IGF2BP3
IGF2R
IGFALS
IGFBP2
IGFBP4
IGFBP5
IGFBP7
IGLON5
IGSF1
IGSF11
IGSF21
IGSF3
IGSF5
IGSF9
IGSF9B
IHH
IKBKB
IKZF3
IKZF4
IKZF5
IL10
IL10RA
IL12A
IL12B
IL12RB1

IL13
IL15
IL16
IL17A
IL17B
IL17D
IL17F
IL17RC
IL17RD
IL1B
IL1R1
IL1RAPL1
IL20RB
IL22
IL23R
IL27RA
IL28A
IL28B
IL28RA
IL2RA
IL3
IL33
IL34
IL3RA
IL4
IL4RA
IL5
IL6RA
IL6ST
IL7
IL7R
IL8RA
IL9R
ILDR1
ILF2
ILF3
ILK
IMMP2L
IMMT
IMPA2
IMPACT
IMPDH2
IMPG2
INA
INADL
INCENP
ING2
ING3
INHA
INHBB
INHBC
INMT
INO80

INO80C
INO80D
INO80E
INPP4A
INPP4B
INPP5A
INPP5B
INPP5D
INPP5F
INPP5J
INPP5K
INPPL1
INS1
INS2
INSIG1
INSIG2
INSL3
INSM1
INSR
INTS10
INTS3
INTS4
INTS6
INTS7
INTS9
INVS
IP6K1
IP6K2
IP6K3
IPCEF1
IPMK
IPO11
IPO5
IPO7
IPPK
IQCA
IQCD
IQCE
IQCF1
IQCF4
IQCF6
IQCH
IQCK
IQGAP1
IQGAP2
IQSEC1
IQSEC2
IQSEC3
IRAK1
IRAK1BP1
IRAK2
IRAK3
IREB2

IRF1
IRF2
IRF2BP2
IRF6
IRF8
IRF9
IRG1
IRGC1
IRGM2
IRS1
IRS2
IRX1
IRX2
IRX3
IRX4
IRX5
IRX6
ISG15
ISG20
ISG20L2
ISL1
ISL2
ISLR
ISLR2
ISOC2A
ISOC2B
ISX
ISY1
ITCH
ITFG2
ITFG3
ITGA1
ITGA10
ITGA11
ITGA3
ITGA5
ITGA6
ITGA8
ITGA9
ITGAE
ITGAL
ITGAM
ITGAV
ITGB1BP2
ITGB2
ITGB3
ITGB3BP
ITGB4
ITGB5
ITGB6
ITGB7
ITGBL1
ITIH2

ITIH4
ITIH5
ITM2B
ITM2C
ITPK1
ITPKA
ITPKB
ITPKC
ITPR1
ITPR2
ITPR3
ITPRIP
ITPRIPL1
ITPRIPL2
ITSN1
ITSN2
IVD
IVNS1ABP
JAG1
JAG2
JAGN1
JAK2
JAK3
JAKMIP1
JAKMIP2
JAKMIP3
JAM2
JAM3
JARID2
JAZF1
JDP2
JHDM1D
JKAMP
JMJD1C
JMJD4
JMJD5
JMJD6
JMJD8
JMY
JOSD2
JPH2
JPH3
JRK
JUB
JUN
JUP
KANK1
KANK2
KANK3
KANK4
KARS
KAT2A
KAT2B

KATNA1
KATNAL1
KATNAL2
KATNB1
KAZALD1
KBTBD11
KBTBD2
KBTBD3
KCMF1
KCNA2
KCNA3
KCNA6
KCNAB2
KCNB1
KCNC1
KCNC2
KCNC3
KCNC4
KCND2
KCNE1
KCNE2
KCNE4
KCNF1
KCNG1
KCNG3
KCNG4
KCNH1
KCNH3
KCNH8
KCNIP3
KCNJ10
KCNJ11
KCNJ12
KCNJ14
KCNJ16
KCNJ2
KCNJ4
KCNJ5
KCNJ6
KCNJ8
KCNK1
KCNK10
KCNK12
KCNK13
KCNK16
KCNK3
KCNK4
KCNK5
KCNK6
KCNK9
KCNMA1
KCNMB2
KCNN1

KCNN3
KCNN4
KCNQ1
KCNQ2
KCNQ3
KCNQ4
KCNS3
KCNT2
KCNU1
KCTD1
KCTD12
KCTD14
KCTD15
KCTD16
KCTD18
KCTD20
KCTD21
KCTD4
KCTD6
KCTD7
KCTD9
KDELC2
KDELR3
KDM1A
KDM2A
KDM2B
KDM3A
KDM3B
KDM4C
KDM5A
KDM5B
KDM6B
KDR
KEAP1
KHDRBS1
KIF11
KIF13A
KIF13B
KIF14
KIF16B
KIF18A
KIF18B
KIF19A
KIF1A
KIF1B
KIF20B
KIF21A
KIF21B
KIF23
KIF24
KIF26A
KIF26B
KIF27

KIF2C
KIF3A
KIF3C
KIF4
KIF5A
KIF5B
KIF5C
KIF6
KIF9
KIFC1
KIFC2
KIFC3
KIN
KIRREL
KIRREL2
KIRREL3
KIS2
KISS1R
KIT
KL
KLB
KLC2
KLC3
KLF10
KLF12
KLF13
KLF14
KLF15
KLF16
KLF17
KLF2
KLF3
KLF4
KLF5
KLF6
KLF7
KLF8
KLF9
KLHDC10
KLHDC4
KLHDC7A
KLHDC7B
KLHDC8A
KLHDC8B
KLHL12
KLHL18
KLHL2
KLHL20
KLHL22
KLHL25
KLHL29
KLHL31
KLHL32

KLHL34
KLHL36
KLHL38
KLHL4
KLHL5
KLHL7
KLK10
KLK15
KLK4
KLK5
KLK6
KLK7
KLK9
KLRAQ1
KLRB1B
KLRB1C
KLRG1
KLRG2
KNCN
KNDC1
KNG1
KPNA2
KPNB1
KRAS
KRBA1
KRCC1
KREMEN1
KREMEN2
KRR1
KRT17
KRT18
KRT19
KRT222
KRT23
KRT33A
KRT33B
KRT42
KRT7
KRT8
KRT80
KRT9
KRTAP11-1
KRTAP17-1
KRTAP2-4
KRTAP31-2
KRTAP4-1
KRTAP7-1
KRTAP9-5
KRTDAP
KSR1
KSR2
KTELC1
KTI12

KTN1
L1CAM
L1TD1
L3MBTL
L3MBTL3
LACE1
LACTB
LACTB2
LAD1
LAG3
LAMA1
LAMA2
LAMA3
LAMA5
LAMB1-1
LAMB2
LAMB3
LAMC1
LAMC2
LAMC3
LAMP1
LAMP2
LANCL1
LANCL2
LAO1
LAP3
LAPTM4A
LAPTM4B
LAPTM5
LARGE
LARP1
LARP4B
LARP6
LARP7
LARS2
LAS1L
LASP1
LASS2
LASS4
LASS5
LASS6
LAT
LATS1
LATS2
LAX1
LBR
LBX1
LBXCOR1
LCA5
LCK
LCLAT1
LCOR
LCORL

LCP1
LCP2
LCT
LCTL
LDB1
LDB2
LDB3
LDHA
LDHB
LDLR
LDLRAD2
LDLRAD3
LDLRAP1
LDOC1
LDOC1L
LECT1
LEF1
LEFTY1
LEKR1
LEMD1
LEMD2
LEMD3
LENEP
LENG8
LEO1
LEP
LEPR
LEPROT
LETM1
LETMD1
LFNG
LGALS2
LGALS3
LGALS3BP
LGALS7
LGALS9
LGI1
LGI2
LGMN
LGR4
LGTN
LHB
LHCGR
LHFPL2
LHFPL3
LHFPL4
LHX1
LHX2
LHX3
LHX4
LHX5
LHX6
LHX8

LHX9
LIF
LIFR
LIG1
LIG4
LIM2
LIMA1
LIMCH1
LIMD1
LIMK1
LIMK2
LIMS1
LIMS2
LIN28
LIN28B
LIN54
LIN9
LINGO1
LINGO2
LINGO3
LINS2
LIPC
LIPG
LIPH
LIPT2
LITAF
LIX1
LIX1L
LLGL1
LLGL2
LLPH
LMAN1
LMAN1L
LMAN2
LMBRD1
LMBRD2
LMCD1
LMNA
LMNB1
LMNB2
LMO2
LMO7
LMOD1
LMOD2
LMOD3
LMX1B
LNP
LNPEP
LNX1
LNX2
LOC100039801
LOC100043315
LOC100233175

LOC100233207
LOC16697
LOC624853
LOC665622
LOH12CR1
LONRF1
LONRF2
LOXHD1
LOXL1
LOXL2
LOXL3
LOXL4
LPAR1
LPAR2
LPAR4
LPAR5
LPAR6
LPCAT2
LPCAT3
LPCAT4
LPGAT1
LPHN1
LPHN3
LPIN1
LPIN2
LPIN3
LPL
LPP
LRAT
LRBA
LRCH1
LRCH3
LRCH4
LRFN1
LRFN2
LRIG1
LRIG2
LRIG3
LRMP
LRP1
LRP10
LRP12
LRP1B
LRP2
LRP3
LRP4
LRP5
LRP6
LRP8
LRPAP1
LRRC1
LRRC10
LRRC15

LRRC2
LRRC20
LRRC24
LRRC25
LRRC26
LRRC28
LRRC30
LRRC32
LRRC33
LRRC34
LRRC38
LRRC4
LRRC41
LRRC45
LRRC49
LRRC4B
LRRC50
LRRC51
LRRC52
LRRC57
LRRC58
LRRC59
LRRC61
LRRC67
LRRC7
LRRC8A
LRRC8B
LRRC8C
LRRC8D
LRRC9
LRRFIP1
LRRIQ4
LRRK1
LRRN2
LRRN4
LRRN4CL
LRSAM1
LRWD1
LSM10
LSM11
LSM14A
LSM14B
LSM2
LSM3
LSM4
LSM6
LST1
LTA4H
LTB
LTB4R1
LTB4R2
LTBP1
LTBP3

LTBP4
LUC7L
LUZP1
LY6C2
LY6E
LY6F
LY6G6D
LY6G6E
LY6K
LY75
LY86
LYN
LYPD4
LYPD6
LYPD6B
LYPLA1
LYRM1
LYRM5
LYSMD1
LYSMD3
LYVE1
LYZL4
LZTFL1
LZTR1
LZTS1
LZTS2
M6PR
MACF1
MACROD1
MACROD2
MAD1L1
MAD2L1
MAD2L1BP
MADCAM1
MADD
MAEA
MAEL
MAF
MAF1
MAFA
MAFB
MAFF
MAFG
MAG
MAGEB18
MAGEB3
MAGED2
MAGEL2
MAGI1
MAGI2
MAGI3
MAGIX
MAK

MAK10
MAK16
MAL
MALAT1
MALT1
MAMDC2
MAML1
MAML2
MAML3
MAMLD1
MAN1A2
MAN1C1
MAN2A1
MAN2B2
MAN2C1
MANBA
MANBAL
MANEA
MANEAL
MANSC1
MAP1LC3B
MAP2K1
MAP2K2
MAP2K3
MAP2K5
MAP2K6
MAP3K1
MAP3K12
MAP3K13
MAP3K14
MAP3K3
MAP3K4
MAP3K5
MAP3K7
MAP3K7IP1
MAP3K7IP2
MAP3K8
MAP3K9
MAP4K1
MAP4K3
MAP4K4
MAP4K5
MAP6D1
MAPK11
MAPK13
MAPK14
MAPK15
MAPK1IP1L
MAPK4
MAPK6
MAPK8
MAPK9
MAPKAP1

MAPKAPK3
MAPKAPK5
MAPRE2
MAPRE3
MAPT
Mar-01
Mar-10
Mar-02
Mar-05
Mar-07
MARCKS
MARCKSL1
MARCO
MARK2
MARK3
MARK4
MARS
MARS2
MARVELD3
MASP2
MAST1
MAST4
MASTL
MAT2A
MAT2B
MATK
MATN1
MATN2
MATN3
MATN4
MAVS
MAX
MB
MBD2
MBD3
MBI-39
MBIP
MBNL1
MBNL2
MBNL3
MBOAT1
MBOAT2
MBP
MBTD1
MBTPS1
MC3R
MC4R
MCART1
MCART6
MCC
MCCC2
MCF2
MCF2L

MCFD2
MCHR1
MCL1
MCM2
MCM3
MCM3AP
MCM5
MCM6
MCM9
MCOLN1
MCOLN2
MCPH1
MCPT-PS1
MCRS1
MCTP2
MCTS2
MDFI
MDFIC
MDGA1
MDH2
MDK
MDM1
MDM2
MDM4
ME2
MECOM
MECP2
MECR
MED1
MED11
MED12
MED13
MED13L
MED14
MED16
MED17
MED18
MED21
MED22
MED23
MED24
MED25
MED26
MED27
MED28
MED29
MED30
MED7
MED8
MED9
MEF2A
MEF2B
MEF2D

MEG3
MEGF10
MEGF11
MEGF8
MEI1
MEIG1
MEIS1
MEIS2
MELK
MEN1
MEOX1
MEOX2
MEP1A
MEP1B
MEPCE
MEPE
MERTK
MESDC1
MESDC2
MESP1
METAP2
METAPL1
METT11D1
METTL1
METTL11A
METTL11B
METTL2
METTL7A1
METTL9
MEX3A
MEX3B
MEX3C
MEX3D
MFAP3
MFAP3L
MFAP5
MFGE8
MFHAS1
MFI2
MFN2
MFNG
MFSD1
MFSD10
MFSD11
MFSD2A
MFSD4
MFSD5
MFSD7A
MFSD7B
MFSD7C
MGA
MGAT1
MGAT3

MGAT4C
MGAT5
MGAT5B
MGEA5
MGL1
MGLL
MGMT
MGP
MGRN1
MGST2
MGST3
MIAT
MIB2
MICAL1
MICAL2
MICAL3
MICALL1
MICALL2
MID1
MIDN
MIER1
MIER2
MIER3
MIF
MILL2
MINA
MINK1
MINPP1
MIR1-1
MIR1-2
MIR122A
MIR124A-1
MIR124A-3
MIR129-1
MIR129-2
MIR130A
MIR133A-2
MIR135B
MIR138-1
MIR145
MIR146
MIR148A
MIR152
MIR17
MIR181A-1
MIR181C
MIR182
MIR183
MIR184
MIR187
MIR190
MIR191
MIR192

MIR196A-1
MIR196B
MIR200B
MIR200C
MIR202
MIR203
MIR205
MIR206
MIR21
MIR210
MIR216B
MIR219-1
MIR23A
MIR24-1
MIR26B
MIR290
MIR296
MIR297-1
MIR298
MIR29A
MIR29B-1
MIR300
MIR302B
MIR30A
MIR30D
MIR31
MIR320
MIR322
MIR33
MIR330
MIR331
MIR34B
MIR350
MIR365-2
MIR448
MIR464
MIR469
MIR511
MIR574
MIR670
MIR674
MIR678
MIR680-2
MIR684-1
MIR688
MIR692-1
MIR702
MIR704
MIR713
MIR7-2
MIR759
MIR760
MIR762

MIR802
MIR874
MIR9-1
MIR9-2
MIR92B
MIR9-3
MIR96
MIRLET7B
MIRLET7C-2
MIRN193
MIS12
MITF
MKI67
MKI67IP
MKKS
MKL1
MKL2
MKLN1
MKNK1
MKRN1
MKRN3
MLANA
MLEC
MLF1IP
MLF2
MLH3
MLL1
MLL2
MLL3
MLL5
MLLT1
MLLT10
MLLT11
MLLT3
MLLT4
MLLT6
MLPH
MLXIP
MMAA
MMD2
MME
MMGT1
MMGT2
MMP11
MMP14
MMP17
MMP2
MMP20
MMP25
MMP28
MMP9
MMRN1
MMRN2

MN1
MND1
MNT
MNX1
MOAP1
MOBKL1A
MOBKL2A
MOBKL2B
MOBKL2C
MOBKL3
MOBP
MOCOS
MOCS2
MOCS3
MOGAT1
MOGAT2
MON1B
MON2
MORC1
MORC2A
MORC3
MORF4L1
MORN1
MORN3
MOSC1
MOSC2
MOSPD4
MOV10
MPDZ
MPHOSPH8
MPHOSPH9
MPP6
MPP7
MPPE1
MPPED1
MPPED2
MPRIP
MPV17L
MPV17L2
MPZL1
MPZL2
MR1
MRAP2
MRAS
MRC2
MREG
MRGPRG
MRM1
MRPL1
MRPL12
MRPL13
MRPL14
MRPL15

MRPL17
MRPL20
MRPL21
MRPL27
MRPL33
MRPL34
MRPL35
MRPL36
MRPL39
MRPL4
MRPL40
MRPL45
MRPL48
MRPL51
MRPS10
MRPS23
MRPS24
MRPS28
MRPS35
MRPS5
MRPS6
MRPS9
MRRF
MRS2
MRVI1
MSC
MSGN1
MSH2
MSH3
MSH4
MSH6
MSI1
MSI2
MSL1
MSL3L2
MSLN
MSMB
MSN
MSRA
MSRB2
MSRB3
MST1R
MSTN
MSX1
MSX1AS
MSX2
MSX3
MT1
MT2
MT3
MTA1
MTA2
MTA3

MTAG2
MTAP1A
MTAP1B
MTAP1S
MTAP2
MTAP4
MTAP6
MTAP7
MTAP7D1
MTCH2
MTDH
MTERF
MTERFD1
MTF1
MTF2
MTFR1
MTHFD1
MTHFD1L
MTHFSD
MTIF2
MTIF3
MTMR12
MTMR14
MTMR15
MTMR3
MTMR4
MTMR7
MTMR9
MTOR
MTR
MTRF1L
MTRR
MTSS1
MTSS1L
MTUS1
MTUS2
MTVR2
MUC13
MUC20
MUC4
MUC5AC
MUC6
MUL1
MUM1
MURC
MUSK
MUTED
MVK
MVP
MX2
MXD1
MXD3
MXD4

MXI1
MXRA7
MYADM
MYB
MYBBP1A
MYBL2
MYBPC1
MYC
MYCBP
MYCBP2
MYCBPAP
MYCL1
MYCN
MYD88
MYH10
MYH11
MYH13
MYH14
MYH2
MYH3
MYH7
MYH7B
MYH9
MYL10
MYL12B
MYL4
MYLIP
MYLK
MYLK2
MYLK3
MYLPF
MYNN
MYO10
MYO15
MYO16
MYO18A
MYO18B
MYO19
MYO1B
MYO1C
MYO1D
MYO1E
MYO1F
MYO1G
MYO1H
MYO3A
MYO3B
MYO5A
MYO5B
MYO5C
MYO6
MYOC
MYOF

MYOM1
MYOM2
MYOM3
MYOT
MYOZ2
MYOZ3
MYPN
MYPOP
MYRIP
MYST1
MYST2
MYST3
MYST4
MYT1
MYT1L
MZF1
N28178
N4BP1
N4BP2
N6AMT2
NAAA
NAB1
NAB2
NACC1
NACC2
NADK
NADSYN1
NAF1
NAGA
NAGK
NAGS
NANOG
NANP
NAP1L1
NAP1L4
NAP1L5
NAPA
NAPB
NAPEPLD
NAPG
NAPRT1
NARFL
NARG1L
NASP
NAT1
NAT10
NAT11
NAT13
NAT6
NAT8
NAV1
NAV2
NBAS

NBEAL1
NBEAL2
NBL1
NBN
NCALD
NCAM1
NCAN
NCAPD2
NCAPH2
NCDN
NCEH1
NCF2
NCK2
NCKAP5
NCKAP5L
NCKIPSD
NCOA1
NCOA3
NCOA5
NCOA6
NCOA7
NCOR1
NCOR2
NCRNA00085
NDC80
NDE1
NDFIP1
NDOR1
NDP
NDRG1
NDRG3
NDRG4
NDST1
NDST3
NDUFA10
NDUFA11
NDUFA12
NDUFA4L2
NDUFA6
NDUFA8
NDUFA9
NDUFAF2
NDUFAF3
NDUFAF4
NDUFB2
NDUFB4
NDUFB5
NDUFB7
NDUFS1
NDUFS4
NDUFS6
NDUFS8
NDUFV1

NDUFV3
NEAT1
NEB
NEBL
NECAB2
NECAB3
NECAP2
NEDD4
NEDD4L
NEDD9
NEFL
NEGR1
NEIL2
NEIL3
NEK10
NEK6
NEK7
NEK8
NEK9
NENF
NEO1
NES
NESPAS
NETO1
NETO2
NEURL1A
NEURL2
NEUROD1
NEUROD2
NEUROD4
NEUROG1
NF1
NF2
NFAM1
NFASC
NFAT5
NFATC1
NFATC2
NFATC2IP
NFATC3
NFATC4
NFE2
NFE2L1
NFE2L2
NFE2L3
NFIB
NFIC
NFIX
NFKB1
NFKB2
NFKBIA
NFKBIB
NFKBIZ

NFU1
NFX1
NFYB
NFYC
NGDN
NGF
NGFR
NGFRAP1
NHEDC1
NHEJ1
NHLH1
NHLH2
NHLRC1
NHLRC2
NHLRC3
NHP2L1
NHS
NHSL1
NHSL2
NICN1
NID1
NID2
NIN
NINJ2
NINL
NIPA1
NIPA2
NIPBL
NIPSNAP1
NIPSNAP3B
NKAIN1
NKAIN4
NKD1
NKIRAS1
NKTR
NKX2-1
NKX2-3
NKX2-6
NKX2-9
NKX6-1
NKX6-2
NKX6-3
NLGN1
NLGN2
NLK
NLN
NLRC4
NLRP10
NLRP12
NLRP14
NLRP3
NLRP4A
NLRP4F

NLRP9C
NMB
NMD3
NME1
NME2
NME4
NME7
NMI
NMNAT2
NMRAL1
NMT1
NMT2
NMUR2
NNAT
NNMT
NNT
NOD1
NODAL
NOL10
NOL4
NOL6
NOL9
NOLC1
NOMO1
NOP10
NOP58
NOS1
NOS1AP
NOS3
NOSTRIN
NOTCH1
NOTCH2
NOTCH3
NOTO
NOTUM
NOV
NOVA1
NOX3
NOXA1
NPAS2
NPAS4
NPBWR1
NPC1
NPC2
NPEPL1
NPEPPS
NPFFR2
NPHP1
NPHP3
NPHP4
NPHS1
NPHS1AS
NPL

NPM1
NPM3
NPNT
NPPB
NPPC
NPR1
NPR3
NPTN
NPTX1
NPTX2
NPTXR
NPY1R
NPY6R
NQO1
NQO2
NR1D1
NR1D2
NR1I3
NR2C2
NR2C2AP
NR2E1
NR2F1
NR2F2
NR2F6
NR3C2
NR4A1
NR4A3
NR5A1
NR5A2
NR6A1
NRARP
NRAS
NRBF2
NRCAM
NRD1
NRF1
NRG1
NRG4
NRIP1
NRM
NRP
NRP2
NRSN1
NRSN2
NRTN
NRXN1
NRXN2
NRXN3
NSD1
NSDHL
NSG2
NSMAF
NSMCE2

NSMCE4A
NSUN2
NSUN4
NSUN6
NSUN7
NT5C2
NT5DC1
NT5DC2
NT5DC3
NT5M
NTF3
NTF5
NTM
NTN1
NTN4
NTNG2
NTRK2
NTRK3
NTS
NTSR2
NUAK1
NUAK2
NUB1
NUBP1
NUCKS1
NUDCD1
NUDCD2
NUDCD3
NUDT1
NUDT14
NUDT16
NUDT19
NUDT2
NUDT4
NUDT7
NUF2
NUFIP1
NUMA1
NUMB
NUMBL
NUP107
NUP153
NUP155
NUP160
NUP188
NUP205
NUP210
NUP210L
NUP214
NUP35
NUP50
NUP54
NUP62CL

NUP93
NUP98
NUPL2
NUSAP1
NUT
NUTF2
NVL
NWD1
NXF1
NXN
NXNL1
NXPH1
NXPH3
NXPH4
NYNRIN
NYX
OAF
OAS2
OAS3
OASL1
OASL2
OBFC1
OBFC2A
OBSL1
OCIAD1
OCIAD2
OCLN
ODC1
ODF1
ODF2
ODF3B
ODF3L1
ODF3L2
ODF4
ODZ2
ODZ3
ODZ4
OFCC1
OGDH
OGDHL
OGT
OIT1
OLA1
OLFM1
OLFM2
OLFML3
OLFR1044
OLFR1122
OLFR1126
OLFR129
OLFR1318
OLFR1338
OLFR1339

OLFR1350
OLFR1371
OLFR1373
OLFR1389
OLFR1390
OLFR1402
OLFR1423
OLFR1431
OLFR1450
OLFR1451
OLFR15
OLFR1513
OLFR1514
OLFR156
OLFR159
OLFR19
OLFR26
OLFR267
OLFR273
OLFR275
OLFR288
OLFR305
OLFR307
OLFR324
OLFR39
OLFR453
OLFR464
OLFR49
OLFR52
OLFR54
OLFR545
OLFR547
OLFR70
OLFR750
OLFR76
OLFR761
OLFR821
OLFR822
OLFR835
OLFR836
OLFR873
OLFR881
OLFR883
OLFR90
OLFR92
OLFR923
OLFR924
OLFR926
OLFR93
OLFR976
OLFR978
OLIG1
OLIG2

OLIG3
OMA1
ONECUT1
ONECUT2
ONECUT3
OOEP
OPA1
OPA3
OPALIN
OPCML
OPLAH
OPN4
OPRL1
OPTC
OPTN
ORAI1
ORAI2
ORAI3
ORC2L
ORC5L
ORF61
ORF63
ORM1
ORM2
ORMDL3
OS9
OSBP
OSBP2
OSBPL10
OSBPL1A
OSBPL2
OSBPL3
OSBPL5
OSBPL6
OSBPL7
OSBPL8
OSBPL9
OSCAR
OSGEP
OSM
OSMR
OSR1
OSR2
OSTF1
OSTM1
OTOF
OTOP1
OTOR
OTOS
OTP
OTUB2
OTUD1
OTUD3

OTUD7A
OTUD7B
OTX1
OTX2
OTX2OS1
OVOL1
OVOL2
OXA1L
OXGR1
OXNAD1
OXR1
OXSM
OXSR1
OXT
P140
P2RX4
P2RX5
P2RX7
P2RY2
P4HA1
P4HA2
PA2G4
PABPC1
PABPC1L
PABPC3
PABPC4
PACRG
PACRGL
PACS1
PACSIN1
PACSIN2
PADI1
PADI2
PADI4
PAFAH1B3
PAFAH2
PAG1
PAIP2
PAIP2B
PAK1
PAK1IP1
PAK2
PAK4
PAK6
PAK7
PALLD
PALM
PALM2
PALMD
PAM
PAN3
PANK1
PANK2

PANK3
PANK4
PAOX
PAPD4
PAPD5
PAPLN
PAPOLA
PAPPA
PAPSS1
PAPSS2
PAQR3
PAQR4
PAQR5
PAQR6
PAQR7
PAQR8
PAQR9
PARD3
PARD3B
PARD6B
PARD6G
PARK2
PARK7
PARL
PARN
PARP1
PARP10
PARP12
PARP14
PARP16
PARP4
PARP6
PARP9
PARS2
PARVA
PARVB
PARVG
PATL2
PATZ1
PAWR
PAX1
PAX2
PAX5
PAX6
PAX6OS1
PAX7
PAX8
PAX9
PAXIP1
PBK
PBLD
PBX1
PBX3

PBX4
PBXIP1
PCBD1
PCBD2
PCBP1
PCBP3
PCBP4
PCCB
PCDH1
PCDH17
PCDH21
PCDH8
PCDH9
PCDHA1
PCDHA10
PCDHA11
PCDHA12
PCDHA2
PCDHA3
PCDHA4
PCDHA5
PCDHA6
PCDHA7
PCDHA8
PCDHA9
PCDHAC1
PCDHAC2
PCDHB22
PCDHGA1
PCDHGA10
PCDHGA11
PCDHGA12
PCDHGA2
PCDHGA3
PCDHGA4
PCDHGA5
PCDHGA6
PCDHGA7
PCDHGA8
PCDHGA9
PCDHGB1
PCDHGB2
PCDHGB4
PCDHGB5
PCDHGB6
PCDHGB7
PCDHGB8
PCDHGC3
PCDHGC4
PCDHGC5
PCF11
PCGF2
PCGF3

PCGF5
PCGF6
PCIF1
PCK1
PCLO
PCMTD1
PCNT
PCNX
PCNXL2
PCOLCE
PCOLCE2
PCP4L1
PCSK1N
PCSK2
PCSK6
PCTK2
PCTK3
PCTP
PCX
PCYOX1L
PCYT1B
PDAP1
PDCD1
PDCD10
PDCD2
PDCD2L
PDCD4
PDCD6IP
PDCD7
PDCL2
PDCL3
PDE10A
PDE11A
PDE1A
PDE1B
PDE3A
PDE3B
PDE4A
PDE4C
PDE4D
PDE4DIP
PDE5A
PDE6A
PDE6C
PDE6H
PDE7B
PDE8A
PDE8B
PDE9A
PDGFA
PDGFB
PDGFC
PDGFD

PDGFRA
PDGFRB
PDGFRL
PDHB
PDHX
PDIA4
PDIA5
PDIA6
PDIK1L
PDK1
PDK3
PDK4
PDLIM1
PDLIM2
PDLIM3
PDLIM5
PDP1
PDP2
PDPK1
PDPN
PDS5A
PDSS1
PDSS2
PDX1
PDXDC1
PDXK
PDZD2
PDZD4
PDZK1
PDZK1IP1
PDZRN3
PDZRN4
PEA15A
PEBP1
PEBP4
PECAM1
PECI
PECR
PEF1
PEG10
PEG3
PELI1
PELI2
PELP1
PEMT
PENK
PEPD
PERP
PET112L
PET117
PEX10
PEX12
PEX14

PEX16
PEX26
PEX7
PFAS
PFDN1
PFDN4
PFKFB3
PFKFB4
PFKM
PFKP
PFN2
PFTK2
PGA5
PGAP1
PGBD1
PGBD5
PGC
PGD
PGF
PGGT1B
PGLYRP2
PGM1
PGM2
PGM3
PGPEP1
PGRMC1
PGRMC2
PGS1
PHACTR1
PHACTR3
PHACTR4
PHB
PHC1
PHC2
PHF12
PHF13
PHF15
PHF16
PHF17
PHF19
PHF2
PHF20
PHF21A
PHF21B
PHF23
PHF3
PHF6
PHF8
PHIP
PHKB
PHKG1
PHKG2
PHLDA1

PHLDA2
PHLDB1
PHLDB2
PHLPP1
PHOSPHO1
PHOSPHO2
PHOX2A
PHOX2B
PHXR4
PHYH
PHYHD1
PI4K2A
PI4K2B
PI4KB
PIAS1
PIAS3
PIAS4
PIBF1
PIF1
PIGH
PIGL
PIGM
PIGT
PIGX
PIGYL
PIGZ
PIK3AP1
PIK3C2B
PIK3C2G
PIK3CA
PIK3CB
PIK3CD
PIK3CG
PIK3IP1
PIK3R1
PIK3R2
PIK3R3
PIK3R5
PIK3R6
PIM1
PIM3
PIN1
PINK1
PION
PIP4K2A
PIP4K2C
PIP5K1B
PIPOX
PISD-PS2
PITPNA
PITPNB
PITPNC1
PITPNM2

PITPNM3
PITX1
PITX2
PIWIL1
PIWIL2
PIWIL4
PJA1
PKD1
PKD1L2
PKD2
PKDCC
PKDREJ
PKHD1
PKIB
PKM2
PKMYT1
PKN1
PKNOX1
PKNOX2
PKP1
PKP2
PKP3
PKP4
PLA1A
PLA2G10
PLA2G12B
PLA2G15
PLA2G16
PLA2G1B
PLA2G2C
PLA2G2D
PLA2G4B
PLA2G4D
PLA2G5
PLA2G6
PLA2G7
PLA2R1
PLAC1
PLAT
PLAU
PLAUR
PLB1
PLBD1
PLCB1
PLCB3
PLCB4
PLCD1
PLCE1
PLCG2
PLCH1
PLCH2
PLCL1
PLCXD1

PLD1
PLD2
PLD3
PLD4
PLD5
PLEC1
PLEKHA1
PLEKHA2
PLEKHA3
PLEKHA4
PLEKHA5
PLEKHA6
PLEKHA7
PLEKHB1
PLEKHB2
PLEKHF1
PLEKHF2
PLEKHG1
PLEKHG3
PLEKHG4
PLEKHG5
PLEKHG6
PLEKHH1
PLEKHH2
PLEKHM1
PLEKHM2
PLEKHM3
PLEKHO2
PLG
PLIN3
PLK1S1
PLK3
PLK5
PLLP
PLOD1
PLP1
PLS1
PLSCR1
PLSCR4
PLTP
PLVAP
PLXDC1
PLXDC2
PLXNA1
PLXNA2
PLXNA4
PLXNB2
PLXNC1
PM20D1
PMAIP1
PMEPA1
PMFBP1
PML

PMM1
PMP22
PMPCA
PMVK
PNKD
PNKP
PNLDC1
PNLIP
PNMA2
PNMAL2
PNO1
PNP1
PNP2
PNPLA1
PNPLA2
PNPLA3
PNPLA7
PNRC1
PNRC2
PODNL1
PODXL
POFUT2
POGZ
POLA1
POLA2
POLD1
POLE2
POLE4
POLG
POLI
POLL
POLQ
POLR1B
POLR1D
POLR1E
POLR2A
POLR2E
POLR2H
POLR3B
POLR3D
POLR3E
POLR3GL
POLR3K
POMC
POMP
POMT2
PON2
POP4
POP5
POPDC2
POR
PORCN
POU2F1

POU2F2
POU2F3
POU3F1
POU3F2
POU3F3
POU4F1
POU4F2
POU5F1
POU6F1
PPA1
PPA2
PPAN
PPAP2A
PPAP2B
PPAPDC1A
PPAPDC3
PPARA
PPARD
PPARG
PPARGC1A
PPARGC1B
PPAT
PPCDC
PPDPF
PPFIA1
PPFIA2
PPFIA4
PPFIBP1
PPFIBP2
PPIA
PPIB
PPIC
PPIE
PPM1A
PPM1B
PPM1D
PPM1H
PPM1J
PPM1K
PPM1L
PPME1
PPOX
PPP1CB
PPP1CC
PPP1R10
PPP1R12A
PPP1R13B
PPP1R13L
PPP1R14A
PPP1R14D
PPP1R15B
PPP1R16A
PPP1R1B

PPP1R2
PPP1R3C
PPP1R9A
PPP1R9B
PPP2CA
PPP2CB
PPP2R1A
PPP2R1B
PPP2R2A
PPP2R2B
PPP2R2C
PPP2R3A
PPP2R3C
PPP2R4
PPP2R5A
PPP2R5C
PPP2R5D
PPP3CA
PPP3CC
PPP4R1L
PPP5C
PPRC1
PPTC7
PPWD1
PPYR1
PQLC1
PRAM1
PRAMEF12
PRAMEF8
PRAMEL5
PRAMEL7
PRC1
PRCC
PRDM1
PRDM10
PRDM12
PRDM14
PRDM16
PRDM2
PRDM9
PRDX1
PRDX2
PRDX3
PRDX6
PRDX6-RS1
PREI4
PRELID2
PRELP
PREP
PREPL
PREX1
PREX2
PRG4

PRICKLE1
PRICKLE2
PRIM2
PRIMA1
PRKAA1
PRKAA2
PRKAB1
PRKAB2
PRKACA
PRKACB
PRKAG1
PRKAG2
PRKAR1A
PRKAR1B
PRKAR2A
PRKAR2B
PRKCA
PRKCB
PRKCD
PRKCDBP
PRKCE
PRKCH
PRKCI
PRKCZ
PRKD2
PRKD3
PRKDC
PRKG1
PRKG2
PRKRA
PRKRIP1
PRKRIR
PRLH
PRLHR
PRLR
PRMT1
PRMT10
PRMT3
PRMT6
PRMT8
PRND
PRNP
PROC
PRODH
PRODH2
PROK2
PROKR1
PROKR2
PROL1
PROS1
PRPF4B
PRPF6
PRPS1

PRPSAP1
PRR14
PRR15
PRR5
PRRC1
PRRG2
PRRG4
PRRT4
PRRX1
PRRX2
PRRXL1
PRSS16
PRSS27
PRSS34
PRSS36
PRSS39
PRSS45
PRSS48
PRSS8
PRTG
PRUNE
PRUNE2
PRX
PSAP
PSAT1
PSD2
PSEN2
PSIP1
PSKH1
PSMA1
PSMA3
PSMA6
PSMA7
PSMB1
PSMB10
PSMB2
PSMB4
PSMB6
PSMB7
PSMB9
PSMC1
PSMC3
PSMC4
PSMC5
PSMC6
PSMD1
PSMD12
PSMD13
PSMD14
PSMD4
PSMD7
PSMD9
PSME2

PSME4
PSMG1
PSMG3
PSMG4
PSORS1C2
PSPH
PSTK
PSTPIP2
PTAR1
PTBP1
PTCH1
PTCH2
PTCHD2
PTDSS1
PTDSS2
PTEN
PTGER3
PTGES
PTGFRN
PTGIS
PTGR1
PTGR2
PTGS1
PTGS2
PTH1R
PTH2
PTHLH
PTK2
PTMA
PTMS
PTOV1
PTP4A1
PTP4A2
PTPDC1
PTPLA
PTPLAD2
PTPLB
PTPN1
PTPN11
PTPN12
PTPN13
PTPN14
PTPN18
PTPN20
PTPN21
PTPN3
PTPN4
PTPN5
PTPN9
PTPRA
PTPRB
PTPRD
PTPRE

PTPRF
PTPRG
PTPRJ
PTPRK
PTPRM
PTPRN2
PTPRO
PTPRQ
PTPRR
PTPRS
PTPRT
PTPRU
PTRF
PTTG1
PTTG1IP
PTX3
PUM1
PURA
PUS7L
PVR
PVRL1
PVRL2
PVRL3
PVRL4
PVT1
PWP2
PWWP2A
PWWP2B
PXDN
PXK
PXMP4
PXN
PYCARD
PYCR2
PYGB
PYGL
PYGO1
PYROXD2
PYY
QARS
QK
QPCT
QRICH2
QSER1
QSOX1
QSOX2
QTRT1
R3HCC1
R3HDM2
R3HDML
R74862
RAB1
RAB11A

RAB11FIP1
RAB11FIP2
RAB11FIP3
RAB11FIP4
RAB11FIP5
RAB12
RAB14
RAB15
RAB17
RAB19
RAB20
RAB21
RAB23
RAB25
RAB27A
RAB2A
RAB30
RAB31
RAB32
RAB34
RAB37
RAB38
RAB39
RAB3A
RAB3B
RAB3D
RAB3GAP1
RAB3GAP2
RAB3IL1
RAB3IP
RAB40B
RAB4A
RAB5A
RAB5C
RAB6
RAB6B
RAB7
RAB7L1
RAB9
RABAC1
RABEP2
RABGAP1
RABGAP1L
RABGEF1
RABGGTB
RABIF
RABL3
RABL4
RAC1
RAC2
RACGAP1
RAD17
RAD18

RAD21
RAD23B
RAD51
RAD52
RAD54B
RAD54L2
RAD9B
RADIL
RAE1
RAF1
RAI1
RAI14
RALA
RALB
RALBP1
RALGAPA1
RALGAPA2
RALGAPB
RALGDS
RALGPS1
RALGPS2
RALY
RAMP1
RAN
RANBP10
RANBP17
RANBP3
RANBP3L
RANBP9
RANGAP1
RAP1B
RAP1GAP
RAP1GAP2
RAP1GDS1
RAP2A
RAP2C
RAPGEF1
RAPGEF3
RAPGEF4
RAPGEF5
RAPH1
RAPSN
RARA
RARB
RARG
RARRES1
RARRES2
RARS
RARS2
RASA2
RASA4
RASAL2
RASD1

RASD2
RASGEF1A
RASGEF1B
RASGRF1
RASGRF2
RASGRP2
RASGRP3
RASL10A
RASL11A
RASL11B
RASSF10
RASSF3
RASSF5
RASSF7
RASSF8
RAVER1
RAX
RB1
RB1CC1
RBBP5
RBBP7
RBBP8
RBBP9
RBKS
RBL2
RBM14
RBM15
RBM17
RBM18
RBM25
RBM26
RBM34
RBM39
RBM4
RBM41
RBM42
RBM44
RBM47
RBM9
RBMS1
RBMS2
RBMS3
RBMX
RBMXL2
RBMXRT
RBP2
RBP7
RBPJ
RBPJL
RBPMS
RBPMS2
RBX1
RC3H1

RCAN1
RCAN2
RCAN3
RCC1
RCC2
RCE1
RCL1
RCN1
RCOR1
RCOR2
RCOR3
RCSD1
RD3
RDH10
RDH14
RDM1
RDX
RECQL5
REEP1
REEP3
REEP4
REEP5
REFBP2
RELA
RELL1
RELL2
RENBP
REPIN1
REPS1
REPS2
RER1
RERE
RERG
REST
RETN
RETNLA
RETNLG
REV1
REV3L
REX2
REXO1
RFC2
RFC4
RFC5
RFESD
RFFL
RFK
RFPL4
RFT1
RFTN1
RFTN2
RFWD2
RFX1

RFX2
RFX3
RFX5
RFX7
RFX8
RFXANK
RG9MTD1
RGL2
RGMA
RGMB
RGN
RGNEF
RGS12
RGS14
RGS16
RGS20
RGS3
RGS5
RGS6
RGS8
RGS9
RGS9BP
RHBDD2
RHBDF1
RHBDF2
RHBDL2
RHBDL3
RHBG
RHCG
RHD
RHEB
RHOB
RHOBTB2
RHOBTB3
RHOF
RHOH
RHOQ
RHOT1
RHOU
RHOV
RHOX9
RHPN1
RHPN2
RIAN
RIBC2
RIC3
RIF1
RILPL1
RIMBP2
RIMBP3
RIMKLA
RIMKLB
RIMS1

RIMS2
RIMS3
RIMS4
RIN1
RIN2
RINL
RIOK1
RIOK3
RIPK4
RIPPLY1
RIPPLY2
RIPPLY3
RIT1
RIT2
RLBP1
RLF
RLIM
RLN1
RLTPR
RMND5B
RN4.5S
RNASE1
RNASE10
RNASE6
RNASE9
RNASEH1
RNASEH2A
RNASEH2B
RNASEH2C
RNASEK
RNASEN
RND1
RND2
RND3
RNF10
RNF103
RNF11
RNF111
RNF112
RNF115
RNF121
RNF122
RNF125
RNF126
RNF13
RNF130
RNF138
RNF139
RNF144A
RNF144B
RNF145
RNF150
RNF157

RNF165
RNF169
RNF17
RNF170
RNF180
RNF181
RNF183
RNF185
RNF187
RNF19A
RNF19B
RNF2
RNF214
RNF216
RNF219
RNF220
RNF31
RNF32
RNF34
RNF38
RNF39
RNF4
RNF43
RNF44
RNF5
RNF7
RNF8
RNFT1
RNGTT
RNH1
RNPC3
RNPEP
RNPS1
RNU1B2
ROBLD3
ROBO1
ROBO4
ROCK1
ROCK2
ROD1
ROR1
ROR2
RORA
RORB
RP1
RP1L1
RP2H
RP9
RPA1
RPAP1
RPAP3
RPF1
RPGRIP1

RPH3A
RPH3AL
RPIA
RPL10A
RPL10L
RPL11
RPL14
RPL17
RPL19
RPL21
RPL22
RPL22L1
RPL24
RPL27
RPL29
RPL37A
RPL38
RPL39L
RPL41
RPL7
RPLP0
RPLP1
RPN1
RPP14
RPP25
RPPH1
RPRD2
RPRL2
RPS10
RPS11
RPS12
RPS13
RPS14
RPS15A
RPS16
RPS17
RPS18
RPS19
RPS20
RPS21
RPS24
RPS26
RPS27A
RPS3
RPS3A
RPS4X
RPS6KA1
RPS6KA2
RPS6KA5
RPS6KB1
RPS6KL1
RPTN
RPTOR

RPUSD4
RRAGA
RRAGB
RRAGC
RRAGD
RRAS2
RREB1
RRM1
RRM2
RRM2B
RRN3
RRP15
RRP1B
RRP8
RRP9
RRS1
RSAD1
RSBN1
RSBN1L
RSC1A1
RSF1
RSHL1
RSL24D1
RSPH4A
RSPH9
RSPO4
RSPRY1
RSRC1
RSU1
RTBDN
RTCD1
RTDR1
RTKN
RTKN2
RTN1
RTN3
RTN4
RTN4IP1
RTN4R
RTN4RL1
RTN4RL2
RTP1
RUFY1
RUFY3
RUFY4
RUNDC2A
RUNX1
RUNX1T1
RUNX2
RUNX3
RUSC1
RUSC2
RWDD2A

RWDD4A
RXFP1
RXFP2
RXRA
RXRG
RYBP
RYK
RYR2
S100A1
S100A10
S100A16
S100A3
S100A6
S100A7A
S100Z
S1PR1
S1PR2
S1PR3
SAAL1
SACS
SAE1
SAFB2
SAG
SALL1
SALL2
SALL3
SALL4
SAMD1
SAMD14
SAMD4
SAMD4B
SAMD5
SAMD7
SAMHD1
SAMM50
SAP130
SAP18
SAP30
SAP30BP
SAP30L
SAPS1
SAPS3
SAR1A
SARM1
SARS
SART1
SASH1
SASH3
SASS6
SAT2
SATB1
SATB2
SBK1

SBK2
SBNO1
SC4MOL
SC5D
SCAI
SCAMP1
SCAMP2
SCAMP3
SCAMP4
SCAMP5
SCAP
SCAPER
SCARA3
SCARB1
SCARB2
SCARF1
SCARF2
SCARNA13
SCARNA3A
SCARNA3B
SCCPDH
SCD1
SCD2
SCD3
SCD4
SCEL
SCFD2
SCG2
SCG3
SCGB1A1
SCGB1C1
SCGB3A1
SCGN
SCHIP1
SCLT1
SCMH1
SCML4
SCN10A
SCN1A
SCN2B
SCN3B
SCN4A
SCN4B
SCN5A
SCN8A
SCN9A
SCNN1A
SCNN1G
SCOC
SCP2
SCPEP1
SCPPPQ1
SCRG1

SCRIB
SCRN1
SCRT1
SCRT2
SCT
SCUBE1
SCUBE2
SCUBE3
SCYL2
SCYL3
SDAD1
SDC3
SDC4
SDCCAG1
SDCCAG3
SDCCAG8
SDHC
SDHD
SDK1
SDK2
SDR9C7
SDSL
SEC11A
SEC11C
SEC14L1
SEC14L3
SEC14L4
SEC23A
SEC23B
SEC23IP
SEC24A
SEC24B
SEC31A
SEC31B
SEC61B
SEC61G
SEC63
SEH1L
SEL1L
SEL1L2
SEL1L3
SELE
SELENBP1
SELENBP2
SELK
SELL
SELPLG
SEMA3B
SEMA3D
SEMA3E
SEMA4A
SEMA4B
SEMA4D

SEMA5A
SEMA5B
SEMA6B
SEMA6C
SEMA6D
SEMA7A
SENP6
SENP7
Sep-15
SEPHS1
SEPHS2
SEPN1
SEPSECS
Sep-01
Sep-11
Sep-03
Sep-06
Sep-08
Sep-09
SEPX1
SERBP1
SERF2
SERGEF
SERHL
SERINC1
SERINC2
SERINC5
SERP2
SERPINA3H
SERPINA3N
SERPINB1A
SERPINB6A
SERPINB6C
SERPINB9
SERPINC1
SERPINE1
SERPING1
SERPINI1
SERTAD2
SERTAD3
SERTAD4
SESN1
SESN2
SESN3
SESTD1
SET
SETBP1
SETD4
SETD5
SETD7
SETDB1
SETMAR
SETX

SEZ6
SEZ6L
SEZ6L2
SF3A1
SF3B1
SF3B5
SFN
SFPI1
SFPQ
SFRP1
SFRP4
SFRS1
SFRS13A
SFRS13B
SFRS15
SFRS18
SFRS2
SFRS2IP
SFRS3
SFRS4
SFRS5
SFRS6
SFRS8
SFRS9
SFTPB
SFXN1
SFXN4
SFXN5
SGCA
SGCB
SGCD
SGCE
SGK1
SGK3
SGMS1
SGMS2
SGOL2
SGPL1
SGPP1
SGSH
SGSM1
SGSM3
SGTA
SGTB
SH2B1
SH2B3
SH2D3C
SH2D4A
SH2D4B
SH2D5
SH3BGR
SH3BGRL3
SH3BP1

SH3BP4
SH3BP5L
SH3GL1
SH3GL2
SH3GL3
SH3KBP1
SH3PXD2A
SH3PXD2B
SH3RF1
SH3RF2
SH3RF3
SH3TC1
SHANK2
SHANK3
SHB
SHBG
SHC1
SHC3
SHC4
SHD
SHE
SHF
SHH
SHISA2
SHISA4
SHISA5
SHISA7
SHKBP1
SHMT2
SHOC2
SHOX2
SHPRH
SHQ1
SHROOM1
SHROOM2
SHROOM3
SI
SIAE
SIAH1A
SIAH1B
SIAH2
SIAH3
SIDT2
SIGIRR
SIGLEC5
SIGLECG
SIK1
SIK2
SIK3
SIKE1
SIL1
SIM1
SIM2

SIN3A
SIN3B
SIP1
SIPA1
SIPA1L1
SIPA1L2
SIPA1L3
SIRPA
SIRT1
SIRT3
SIRT4
SIRT6
SIT1
SIX1
SIX2
SIX3
SIX3OS1
SIX4
SIX6
SIX6OS1
SKAP1
SKAP2
SKI
SKIL
SKP1A
SKP2
SLAIN1
SLAIN2
SLAMF6
SLAMF9
SLBP
SLC10A1
SLC10A7
SLC11A2
SLC12A1
SLC12A2
SLC12A3
SLC12A4
SLC12A5
SLC12A7
SLC12A8
SLC12A9
SLC13A3
SLC13A5
SLC14A2
SLC15A1
SLC16A1
SLC16A11
SLC16A13
SLC16A14
SLC16A5
SLC16A6
SLC16A7

SLC16A9
SLC17A5
SLC17A7
SLC17A9
SLC19A1
SLC19A2
SLC19A3
SLC1A1
SLC1A3
SLC1A4
SLC1A5
SLC1A7
SLC20A1
SLC22A14
SLC22A16
SLC22A3
SLC22A5
SLC22A6
SLC22A8
SLC23A1
SLC23A2
SLC24A1
SLC24A2
SLC24A3
SLC24A4
SLC24A5
SLC24A6
SLC25A1
SLC25A10
SLC25A11
SLC25A12
SLC25A13
SLC25A14
SLC25A15
SLC25A16
SLC25A17
SLC25A19
SLC25A2
SLC25A21
SLC25A22
SLC25A24
SLC25A25
SLC25A26
SLC25A27
SLC25A28
SLC25A3
SLC25A30
SLC25A33
SLC25A34
SLC25A36
SLC25A39
SLC25A4
SLC25A40

SLC25A42
SLC25A43
SLC25A5
SLC26A10
SLC26A11
SLC26A2
SLC26A4
SLC26A5
SLC26A8
SLC26A9
SLC27A1
SLC27A3
SLC27A4
SLC27A5
SLC28A1
SLC28A3
SLC29A1
SLC29A2
SLC29A3
SLC29A4
SLC2A1
SLC2A10
SLC2A12
SLC2A2
SLC2A3
SLC2A4
SLC2A6
SLC2A7
SLC2A9
SLC30A1
SLC30A10
SLC30A2
SLC30A5
SLC31A2
SLC32A1
SLC34A2
SLC34A3
SLC35A1
SLC35A3
SLC35B1
SLC35B2
SLC35B3
SLC35C1
SLC35C2
SLC35D1
SLC35E4
SLC35F1
SLC35F2
SLC35F4
SLC35F5
SLC36A2
SLC36A3
SLC37A2

SLC37A3
SLC38A1
SLC38A10
SLC38A2
SLC38A4
SLC38A7
SLC38A8
SLC38A9
SLC39A10
SLC39A11
SLC39A12
SLC39A14
SLC39A3
SLC39A4
SLC39A8
SLC39A9
SLC3A2
SLC40A1
SLC41A1
SLC41A2
SLC41A3
SLC43A1
SLC43A2
SLC43A3
SLC44A1
SLC44A2
SLC44A3
SLC44A5
SLC45A1
SLC45A3
SLC45A4
SLC46A1
SLC46A3
SLC47A1
SLC48A1
SLC4A1
SLC4A10
SLC4A11
SLC4A1AP
SLC4A3
SLC4A4
SLC4A5
SLC4A8
SLC4A9
SLC5A11
SLC5A4A
SLC5A4B
SLC5A5
SLC5A6
SLC6A1
SLC6A11
SLC6A12
SLC6A14

SLC6A15
SLC6A17
SLC6A20A
SLC6A3
SLC6A5
SLC6A6
SLC6A7
SLC6A8
SLC6A9
SLC7A1
SLC7A10
SLC7A11
SLC7A12
SLC7A14
SLC7A15
SLC7A5
SLC7A8
SLC8A3
SLC9A1
SLC9A3R1
SLC9A8
SLC9A9
SLCO2A1
SLCO4A1
SLCO5A1
SLFN5
SLFN9
SLIT1
SLIT2
SLIT3
SLK
SLMO1
SLN
SMAD1
SMAD2
SMAD3
SMAD4
SMAD6
SMAD7
SMAGP
SMAP1
SMARCA2
SMARCA4
SMARCAD1
SMARCC1
SMARCC2
SMARCD1
SMARCD2
SMARCD3
SMARCE1
SMC2
SMC3
SMCHD1

SMCR7
SMCR7L
SMG1
SMG6
SMG7
SMOC1
SMOC2
SMOX
SMPD1
SMPD3
SMPD4
SMPDL3B
SMS
SMTN
SMTNL2
SMU1
SMUG1
SMURF1
SMURF2
SMYD1
SMYD3
SNAI1
SNAP91
SNAPC3
SNCAIP
SNCG
SND1
SNED1
SNF8
SNHG10
SNHG11
SNHG6
SNN
SNORA74A
SNORA75
SNORD12
SNORD58B
SNORD72
SNRNP200
SNRNP48
SNRPA
SNRPB2
SNRPE
SNRPN
SNTA1
SNTB2
SNTG1
SNTG2
SNTN
SNUPN
SNX1
SNX10
SNX11

SNX12
SNX15
SNX17
SNX18
SNX2
SNX20
SNX21
SNX24
SNX25
SNX26
SNX29
SNX3
SNX30
SNX31
SNX32
SNX4
SNX5
SNX6
SNX8
SNX9
SOAT1
SOAT2
SOBP
SOCS1
SOCS2
SOCS3
SOCS5
SOCS6
SOCS7
SOD1
SOD2
SOD3
SOLH
SORBS1
SORBS2
SORBS3
SORCS1
SORCS2
SORCS3
SORL1
SORT1
SOS1
SOST
SOX1
SOX10
SOX11
SOX12
SOX13
SOX14
SOX17
SOX18
SOX2
SOX21

SOX2OT
SOX3
SOX30
SOX4
SOX5
SOX6
SOX7
SOX9
SP1
SP100
SP110
SP2
SP3
SP4
SP5
SP6
SP7
SP8
SPACA1
SPACA3
SPAG16
SPAG17
SPAG6
SPAG8
SPAG9
SPAM1
SPARC
SPARCL1
SPATA1
SPATA13
SPATA17
SPATA19
SPATA2
SPATA21
SPATA22
SPATA24
SPATA2L
SPATA3
SPATA5
SPATA6
SPATC1
SPATS1
SPATS2
SPC24
SPC25
SPCS3
SPDEF
SPEER3
SPEER5-PS1
SPEF1
SPEF2
SPEG
SPEN

SPESP1
SPG11
SPG21
SPG7
SPHK2
SPIB
SPIC
SPIN4
SPINK2
SPINT1
SPINT2
SPIRE1
SPIRE2
SPNA2
SPNB1
SPNB2
SPNB3
SPNB4
SPNS2
SPNS3
SPO11
SPOCD1
SPOCK1
SPOCK2
SPOP
SPP1
SPP2
SPPL3
SPR
SPRED1
SPRED2
SPRED3
SPRY1
SPRY2
SPRY4
SPRYD3
SPSB1
SPSB3
SPSB4
SPTLC2
SPTLC3
SPZ1
SQLE
SRBD1
SRC
SRCRB4D
SRD5A1
SRD5A3
SREBF1
SREBF2
SRF
SRFBP1
SRGAP1

SRGAP2
SRGAP3
SRL
SRP14
SRP19
SRP68
SRP9
SRPK1
SRPK2
SRPR
SRR
SRRM1
SRRM2
SRRM3
SRRM4
SRXN1
SS18
SSBP2
SSBP3
SSBP4
SSFA2
SSH1
SSH2
SSPN
SSPO
SSR2
SSR3
SSTR2
SSTR3
SSU72
SSX2IP
ST13
ST14
ST3GAL2
ST3GAL3
ST3GAL4
ST3GAL5
ST5
ST6GAL1
ST6GAL2
ST6GALNAC2
ST6GALNAC3
ST6GALNAC4
ST6GALNAC5
ST6GALNAC6
ST7
ST8SIA1
ST8SIA2
ST8SIA3
ST8SIA4
ST8SIA5
ST8SIA6
STAB2

STAC
STAC2
STAC3
STAG1
STAMBPL1
STAP1
STAR
STARD10
STARD13
STARD3NL
STARD4
STARD8
STAT3
STAT5B
STC1
STC2
STEAP2
STEAP3
STFA3
STIM1
STIM2
STIP1
STK10
STK11IP
STK24
STK3
STK30
STK31
STK32A
STK32C
STK35
STK38
STK38L
STK39
STMN1
STMN2
STMN3
STMN4
STOM
STOML1
STOML2
STON1
STON2
STOX2
STRA13
STRA8
STRADA
STRADB
STRAP
STRC
STRN
STRN3
STT3B

STUB1
STX11
STX12
STX16
STX1B
STX2
STX3
STX4A
STX6
STX7
STX8
STXBP1
STXBP2
STXBP5
STXBP5L
STYK1
SUB1
SUCLA2
SUCLG1
SUCLG2
SUDS3
SUFU
SUGT1
SUHW4
SULF1
SULF2
SULT1C1
SULT5A1
SULT6B1
SUMF1
SUMO2
SUMO3
SUNC1
SUSD2
SUSD4
SUSD5
SUV39H1
SUV39H2
SUV420H1
SUZ12
SV2A
SV2C
SVIL
SVIP
SVOP
SVS1
SWAP70
SYAP1
SYCN
SYCP1
SYDE2
SYK
SYN1

SYN3
SYNC
SYNCRIP
SYNE1
SYNE2
SYNGR2
SYNJ2
SYNPO
SYNPO2L
SYNPR
SYP
SYPL
SYS1
SYT1
SYT11
SYT13
SYT15
SYT16
SYT2
SYT3
SYT4
SYT6
SYT7
SYT8
SYT9
SYTL1
SYTL2
SYVN1
T
T2
TAAR5
TAAR6
TAAR9
TAC2
TAC4
TACC1
TACC2
TACC3
TACR1
TACSTD2
TADA2A
TADA3
TAF1
TAF13
TAF15
TAF1D
TAF3
TAF4A
TAF5L
TAF7
TAF8
TAF9
TAGAP

TAGLN2
TAL1
TAL2
TALDO1
TANC1
TANC2
TANK
TAOK1
TAOK3
TAPBP
TAPT1
TARBP1
TARS
TARSL2
TAS1R2
TAS2R118
TASP1
TAX1BP1
TBATA
TBC1D1
TBC1D10A
TBC1D10C
TBC1D12
TBC1D14
TBC1D15
TBC1D16
TBC1D2
TBC1D21
TBC1D22B
TBC1D23
TBC1D24
TBC1D25
TBC1D2B
TBC1D30
TBC1D4
TBC1D7
TBC1D8
TBC1D9
TBC1D9B
TBCA
TBCB
TBCC
TBCCD1
TBCD
TBCEL
TBK1
TBKBP1
TBL1X
TBL1XR1
TBR1
TBRG1
TBRG4
TBX10

TBX15
TBX2
TBX20
TBX21
TBX3
TBX4
TBX5
TBXAS1
TCAM1
TCEA1
TCEA2
TCEB1
TCEB3
TCF12
TCF15
TCF19
TCF20
TCF21
TCF23
TCF25
TCF3
TCF4
TCF7
TCF7L1
TCF7L2
TCFAP2A
TCFAP2C
TCFAP4
TCFCP2
TCFCP2L1
TCFE3
TCFEB
TCHH
TCHP
TCL1
TCL1B2
TCOF1
TCP1
TCP11
TCP11L1
TCP11L2
TCTA
TCTE1
TCTE2
TCTEX1D1
TCTN2
TDGF1
TDH
TDRD12
TDRD3
TDRD5
TDRD7
TDRD9

TEAD1
TEAD2
TEAD3
TEAD4
TEC
TECPR1
TECPR2
TECR
TECT1
TECTA
TEDDM1
TEF
TEK
TEKT2
TEKT3
TEKT5
TELO2
TENC1
TEP1
TERC
TERF1
TERF2
TERF2IP
TERT
TESC
TESK2
TET1
TET2
TEX10
TEX14
TEX15
TEX19.1
TEX2
TEX21
TEX261
TEX264
TEX9
TFB1M
TFDP1
TFPI
TFRC
TG
TGDS
TGFA
TGFBI
TGFBR1
TGFBR2
TGFBR3
TGIF1
TGIF2
TGM1
TGM2
TGM3

TGS1
TH1L
THA1
THADA
THAP1
THAP6
THBD
THBS1
THBS3
THBS4
THEG
THEM4
THEMIS
THNSL2
THOC2
THOC3
THOC4
THPO
THRA
THRAP3
THRB
THRSP
THSD4
THSD7A
THUMPD1
THUMPD2
THUMPD3
THY1
TIAL1
TIAM1
TIAM2
TICAM1
TICAM2
TIFAB
TIGD2
TIGD3
TIGD4
TIMD4
TIMM17A
TIMM50
TIMP2
TIMP3
TINAGL1
TIPARP
TIPIN
TIPRL
TJAP1
TJP2
TJP3
TK1
TKT
TKTL2
TLCD1

TLE3
TLE4
TLK1
TLK2
TLL1
TLL2
TLN1
TLN2
TLR1
TLR2
TLR4
TLR5
TLR8
TLR9
TLX1
TLX2
TLX3
TM2D1
TM2D3
TM4SF20
TM4SF5
TM6SF1
TM7SF2
TM7SF3
TM7SF4
TM9SF2
TM9SF3
TMBIM1
TMBIM4
TMBIM6
TMC1
TMC3
TMC6
TMC7
TMC8
TMCC1
TMCC2
TMCC3
TMCO4
TMCO5
TMCO5B
TMCO6
TMCO7
TMED10
TMED6
TMED8
TMED9
TMEFF1
TMEM100
TMEM102
TMEM104
TMEM106A
TMEM106B

TMEM106C
TMEM107
TMEM108
TMEM11
TMEM114
TMEM115
TMEM117
TMEM119
TMEM120A
TMEM121
TMEM123
TMEM125
TMEM130
TMEM131
TMEM132C
TMEM132D
TMEM132E
TMEM135
TMEM139
TMEM141
TMEM144
TMEM146
TMEM150C
TMEM151B
TMEM154
TMEM158
TMEM160
TMEM161B
TMEM163
TMEM165
TMEM167
TMEM17
TMEM170
TMEM171
TMEM173
TMEM175
TMEM176A
TMEM177
TMEM179
TMEM180
TMEM183A
TMEM184B
TMEM184C
TMEM188
TMEM19
TMEM194B
TMEM195
TMEM2
TMEM20
TMEM200A
TMEM201
TMEM202
TMEM203

TMEM205
TMEM209
TMEM211
TMEM212
TMEM219
TMEM22
TMEM220
TMEM222
TMEM229A
TMEM229B
TMEM231
TMEM232
TMEM233
TMEM26
TMEM28
TMEM30B
TMEM30C
TMEM33
TMEM37
TMEM38B
TMEM39A
TMEM40
TMEM41B
TMEM44
TMEM45A
TMEM45B
TMEM47
TMEM48
TMEM49
TMEM50A
TMEM50B
TMEM51
TMEM52
TMEM53
TMEM54
TMEM55A
TMEM55B
TMEM57
TMEM59L
TMEM63A
TMEM63B
TMEM63C
TMEM64
TMEM65
TMEM68
TMEM74
TMEM79
TMEM8
TMEM80
TMEM82
TMEM85
TMEM87B
TMEM88

TMEM89
TMEM8B
TMEM9
TMEM90B
TMEM91
TMEM92
TMEM93
TMEM97
TMEM98
TMEM9B
TMF1
TMHS
TMIE
TMOD1
TMOD2
TMOD3
TMPO
TMPRSS11A
TMPRSS11D
TMPRSS13
TMPRSS2
TMPRSS5
TMSB10
TMSB4X
TMTC1
TMTC2
TMTC3
TMUB1
TNF
TNFAIP2
TNFAIP3
TNFAIP6
TNFAIP8
TNFAIP8L1
TNFAIP8L3
TNFRSF10B
TNFRSF11A
TNFRSF13B
TNFRSF13C
TNFRSF14
TNFRSF17
TNFRSF19
TNFRSF1B
TNFRSF21
TNFRSF8
TNFSF10
TNFSF11
TNFSF12-TNFSF13
TNFSF15
TNFSF18
TNFSF4
TNFSF9
TNIK

TNIP1
TNIP2
TNK1
TNK2
TNKS
TNKS1BP1
TNKS2
TNN
TNNI1
TNNT1
TNNT2
TNNT3
TNP1
TNPO1
TNPO2
TNPO3
TNR
TNRC18
TNRC6A
TNRC6B
TNRC6C
TNS1
TNS3
TNS4
TNXB
TOB1
TOB2
TOLLIP
TOM1L1
TOM1L2
TOMM20
TOMM40
TOMM5
TOMM7
TOMM70A
TOP1
TOP1MT
TOP2A
TOP2B
TOP3A
TOP3B
TOPBP1
TOPORS
TOR1A
TOR1B
TOR2A
TOR3A
TOX
TOX2
TOX3
TPBG
TPCN1
TPCN2

TPD52
TPD52L1
TPH2
TPM1
TPM3
TPR
TPRA1
TPRKB
TPST1
TPST2
TPT1
TPT1P
TPX2
TRA2B
TRAF1
TRAF2
TRAF3
TRAF3IP1
TRAF3IP2
TRAF3IP3
TRAF4
TRAFD1
TRAIP
TRAK1
TRAK2
TRAM1
TRAM2
TRANK1
TRAPPC10
TRAPPC3
TRAPPC4
TRAPPC9
TRCG1
TRDN
TREML2
TREML4
TRERF1
TRFR2
TRH
TRHDE
TRHR2
TRIB1
TRIB2
TRIB3
TRIM11
TRIM13
TRIM14
TRIM16
TRIM17
TRIM2
TRIM24
TRIM25
TRIM26

TRIM27
TRIM28
TRIM29
TRIM3
TRIM31
TRIM33
TRIM34
TRIM36
TRIM41
TRIM44
TRIM45
TRIM47
TRIM54
TRIM56
TRIM59
TRIM6
TRIM60
TRIM62
TRIM63
TRIM66
TRIM67
TRIM71
TRIM72
TRIM8
TRIM9
TRIML1
TRIML2
TRIO
TRIOBP
TRIP11
TRIP12
TRIP13
TRIP4
TRIT1
TRNAU1AP
TRNP1
TROAP
TROVE2
TRP53
TRP53BP2
TRP53I11
TRP53INP2
TRP53RK
TRP63
TRP73
TRPC1
TRPC7
TRPM1
TRPM3
TRPM4
TRPM7
TRPS1
TRPV2

TRPV4
TRRAP
TRUB1
TSC1
TSC22D1
TSC22D2
TSC22D3
TSC22D4
TSEN2
TSFM
TSGA10
TSGA14
TSHZ2
TSHZ3
TSIX
TSKS
TSKU
TSN
TSNAXIP1
TSPAN11
TSPAN12
TSPAN13
TSPAN14
TSPAN15
TSPAN17
TSPAN18
TSPAN32
TSPAN33
TSPAN4
TSPAN5
TSPAN6
TSPAN8
TSPAN9
TSPO
TSPYL1
TSPYL5
TSR2
TSSC1
TSSC4
TSTA3
TSTD2
TSX
TTBK1
TTC1
TTC12
TTC13
TTC15
TTC18
TTC19
TTC21A
TTC21B
TTC25
TTC28

TTC29
TTC32
TTC34
TTC38
TTC39B
TTC39C
TTC5
TTC7
TTC7B
TTC8
TTC9
TTC9B
TTF1
TTL
TTLL1
TTLL11
TTLL12
TTLL13
TTLL5
TTLL6
TTLL8
TTN
TTPA
TTPAL
TTR
TTYH1
TTYH2
TTYH3
TUB
TUBA1A
TUBA1C
TUBA3B
TUBA4A
TUBB2A
TUBB2B
TUBB3
TUBB6
TUBD1
TUBG1
TUBG2
TUBGCP3
TUFM
TUFT1
TULP1
TULP2
TULP3
TUSC1
TUSC3
TUSC4
TWF1
TWIST2
TWISTNB
TWSG1

TXLNB
TXN1
TXN2
TXNDC12
TXNDC16
TXNDC3
TXNDC5
TXNDC6
TXNIP
TXNL1
TXNRD1
TXNRD2
TYK2
TYMS
TYRO3
TYRP1
TYSND1
TYW3
U2AF1
U46068
UACA
UAP1L1
UBA2
UBAC2
UBAP2
UBAP2L
UBASH3B
UBB
UBC
UBE2A
UBE2D1
UBE2D2
UBE2D3
UBE2E2
UBE2F
UBE2G1
UBE2G2
UBE2H
UBE2I
UBE2J1
UBE2K
UBE2L3
UBE2M
UBE2O
UBE2Q1
UBE2Q2
UBE2QL1
UBE2R2
UBE2S
UBE2V1
UBE2W
UBE3C
UBE4B

UBL3
UBL4B
UBL7
UBLCP1
UBN2
UBP1
UBQLN1
UBQLN4
UBR1
UBR2
UBR4
UBR5
UBR7
UBTD1
UBTD2
UBTF
UBXN1
UBXN10
UBXN11
UBXN2A
UBXN2B
UBXN8
UCHL3
UCK2
UCMA
UCP1
UEVLD
UFC1
UGCG
UGDH
UGGT1
UGGT2
UGT1A1
UGT1A10
UGT1A2
UGT1A5
UGT1A6A
UGT1A6B
UGT1A7C
UGT1A9
UGT2B34
UGT3A1
UHRF1
UHRF1BP1
UHRF1BP1L
UHRF2
UIMC1
ULK1
ULK2
ULK3
ULK4
UMODL1
UMPS

UNC13A
UNC13B
UNC45A
UNC45B
UNC5A
UNC5B
UNC5C
UNC5CL
UNC84A
UNC84B
UNC93B1
UNCX
UNG
UNK
UPB1
UPF1
UPF2
UPF3A
UPK1A
UPK2
UPK3A
UPK3B
UPP1
UPP2
UQCC
UQCR
UQCRB
UQCRC1
UQCRQ
URM1
USE1
USH2A
USHBP1
USO1
USP1
USP10
USP12
USP13
USP15
USP16
USP2
USP20
USP22
USP24
USP25
USP26
USP28
USP29
USP31
USP33
USP36
USP37
USP38

USP4
USP43
USP44
USP46
USP48
USP49
USP53
USP6NL
USP7
USP8
USP9X
USPL1
UST
UTF1
UTP11L
UTP14A
UTP14B
UTP20
UTP23
UTP3
UTRN
UTS2R
UTY
UVRAG
UXS1
V1RC2
V1RC25
V1RE1
V1RE8
V1RJ3
VAMP2
VAMP4
VANGL1
VANGL2
VAPA
VAPB
VARS
VASH1
VASH2
VASP
VAT1
VAT1L
VAV1
VAV2
VAV3
VAX1
VAX2OS1
VAX2OS2
VBP1
VCAM1
VCAN
VCL
VCP

VDAC1
VDR
VEGFA
VEPH1
VEZF1
VGF
VGLL3
VGLL4
VHL
VIL1
VILL
VIM
VIT
VKORC1L1
VMN2R100
VMN2R107
VMN2R108
VMN2R112
VMN2R24
VMN2R66
VMN2R67
VMN2R87
VMN2R90
VMN2R-PS14
VNN3
VPREB2
VPS11
VPS13B
VPS13D
VPS18
VPS24
VPS28
VPS37B
VPS37C
VPS37D
VPS39
VPS41
VPS45
VPS4A
VPS53
VPS72
VPS8
VRK1
VSIG8
VSTM2L
VSX1
VSX2
VTA1
VTI1A
VTN
VWA2
VWA3A
VWA5B1

VWC2
VWC2L
VWCE
VWF
WAPAL
WASF1
WASF2
WASF3
WASL
WBP1
WBP11
WBP2
WBP2NL
WBP5
WBP7
WBSCR16
WBSCR17
WBSCR25
WBSCR27
WDFY2
WDFY3
WDFY4
WDHD1
WDR1
WDR13
WDR19
WDR20A
WDR25
WDR26
WDR34
WDR36
WDR37
WDR38
WDR4
WDR41
WDR43
WDR45
WDR47
WDR5
WDR51B
WDR52
WDR59
WDR61
WDR62
WDR63
WDR7
WDR70
WDR73
WDR75
WDR81
WDR82
WDR89
WDR91

WDR93
WDTC1
WDYHV1
WEE1
WFDC1
WFDC2
WFDC6A
WFS1
WHAMM
WHRN
WHSC1
WHSC1L1
WIBG
WIF1
WIPF1
WIPF2
WIPI1
WIPI2
WISP2
WISP3
WIZ
WNK1
WNK2
WNT10A
WNT11
WNT2B
WNT3
WNT3A
WNT4
WNT5B
WNT6
WNT7B
WNT8B
WRAP53
WRNIP1
WSB1
WSB2
WSCD1
WSCD2
WWC1
WWC2
WWOX
WWP1
WWP2
WWTR1
X99384
XAF1
XCL1
XIAP
XIRP1
XKR5
XKR8
XPA

XPNPEP1
XPNPEP2
XPNPEP3
XPO1
XPO4
XPO6
XPO7
XPOT
XPR1
XRCC1
XRCC2
XRCC5
XRCC6BP1
XRN2
XYLB
XYLT1
YAF2
YAP1
YARS
YARS2
YBX2
YEATS4
YIPF1
YIPF3
YIPF5
YIPF6
YPEL1
YPEL2
YPEL3
YPEL4
YPEL5
YTHDC1
YTHDF3
YWHAB
YWHAE
YWHAG
YWHAH
YWHAQ
YWHAZ
ZADH2
ZAN
ZAP70
ZBBX
ZBED3
ZBED4
ZBP1
ZBTB1
ZBTB10
ZBTB12
ZBTB16
ZBTB17
ZBTB2
ZBTB20

ZBTB24
ZBTB25
ZBTB32
ZBTB34
ZBTB37
ZBTB38
ZBTB39
ZBTB4
ZBTB40
ZBTB42
ZBTB43
ZBTB44
ZBTB45
ZBTB46
ZBTB48
ZBTB7A
ZBTB7B
ZBTB7C
ZBTB8A
ZBTB8B
ZBTB9
ZC3H11A
ZC3H12A
ZC3H12D
ZC3H13
ZC3H15
ZC3H18
ZC3H3
ZC3H4
ZC3H6
ZC3H7A
ZC3H8
ZC3HAV1L
ZC3HC1
ZCCHC10
ZCCHC12
ZCCHC14
ZCCHC16
ZCCHC17
ZCCHC24
ZCCHC3
ZCCHC7
ZCCHC9
ZCRB1
ZDBF2
ZDHHC1
ZDHHC13
ZDHHC14
ZDHHC17
ZDHHC18
ZDHHC19
ZDHHC2
ZDHHC20

ZDHHC21
ZDHHC22
ZDHHC25
ZDHHC7
ZDHHC9
ZEB1
ZEB2
ZFAND1
ZFAND2A
ZFAND5
ZFAT
ZFHX2
ZFHX3
ZFHX4
ZFML
ZFP1
ZFP106
ZFP11
ZFP110
ZFP111
ZFP113
ZFP114
ZFP12
ZFP120
ZFP13
ZFP142
ZFP146
ZFP148
ZFP157
ZFP160
ZFP161
ZFP184
ZFP185
ZFP191
ZFP202
ZFP212
ZFP213
ZFP217
ZFP219
ZFP229
ZFP236
ZFP248
ZFP251
ZFP263
ZFP27
ZFP273
ZFP28
ZFP280B
ZFP280C
ZFP281
ZFP282
ZFP286
ZFP287

ZFP292
ZFP3
ZFP316
ZFP319
ZFP322A
ZFP324
ZFP326
ZFP329
ZFP335
ZFP341
ZFP345
ZFP346
ZFP354A
ZFP358
ZFP36
ZFP362
ZFP365
ZFP366
ZFP369
ZFP36L1
ZFP36L2
ZFP37
ZFP382
ZFP385A
ZFP385B
ZFP385C
ZFP386
ZFP39
ZFP395
ZFP398
ZFP40
ZFP407
ZFP41
ZFP410
ZFP42
ZFP423
ZFP428
ZFP438
ZFP444
ZFP445
ZFP446
ZFP449
ZFP451
ZFP455
ZFP458
ZFP459
ZFP462
ZFP467
ZFP493
ZFP498
ZFP503
ZFP507
ZFP512

ZFP513
ZFP516
ZFP518B
ZFP521
ZFP526
ZFP532
ZFP536
ZFP54
ZFP541
ZFP568
ZFP57
ZFP59
ZFP592
ZFP598
ZFP60
ZFP606
ZFP607
ZFP608
ZFP609
ZFP612
ZFP617
ZFP618
ZFP619
ZFP628
ZFP629
ZFP637
ZFP639
ZFP64
ZFP641
ZFP644
ZFP646
ZFP647
ZFP651
ZFP652
ZFP655
ZFP658
ZFP661
ZFP667
ZFP668
ZFP687
ZFP689
ZFP691
ZFP692
ZFP7
ZFP703
ZFP704
ZFP706
ZFP708
ZFP710
ZFP712
ZFP715
ZFP719
ZFP71-RS1

ZFP72
ZFP74
ZFP740
ZFP760
ZFP763
ZFP771
ZFP775
ZFP777
ZFP78
ZFP780B
ZFP788
ZFP799
ZFP800
ZFP809
ZFP810
ZFP811
ZFP819
ZFP820
ZFP825
ZFP827
ZFP831
ZFP839
ZFP84
ZFP846
ZFP867
ZFP869
ZFP870
ZFP872
ZFP882
ZFP90
ZFPM1
ZFPM2
ZFR
ZFR2
ZFX
ZFY1
ZFYVE1
ZFYVE16
ZFYVE26
ZFYVE27
ZFYVE28
ZFYVE9
ZG16
ZGLP1
ZHX2
ZIC2
ZIC3
ZIC4
ZIC5
ZIM1
ZKSCAN1
ZKSCAN16
ZKSCAN17

ZKSCAN3
ZKSCAN5
ZMAT3
ZMAT4
ZMIZ1
ZMIZ2
ZMYM1
ZMYM3
ZMYM4
ZMYM6
ZMYND11
ZMYND12
ZMYND19
ZMYND8
ZNHIT2
ZNHIT6
ZNRF1
ZNRF2
ZNRF3
ZNRF4
ZP3R
ZPBP
ZPBP2
ZRANB1
ZRANB2
ZRANB3
ZRSR1
ZRSR2
ZSCAN10
ZSCAN18
ZSCAN2
ZSCAN21
ZSCAN22
ZSCAN5B
ZSWIM1
ZSWIM3
ZSWIM5
ZSWIM6
ZW10
ZWINT
ZXDC
ZYG11B
ZYX
ZZEF1
ZZZ3
