# Supplementary material for: Identification of the missing pluripotency mediator downstream of leukaemia inhibitory factor
Source: EMBO J. 2013 Aug 13;32(19):2561–74. doi: 10.1038/emboj.2013.177 (PMC3791366; doi:10.1038/emboj.2013.177)
Supplement: Source data for Figure 4 [file emboj2013177df4.pdf]

Source data related to Figure 4B

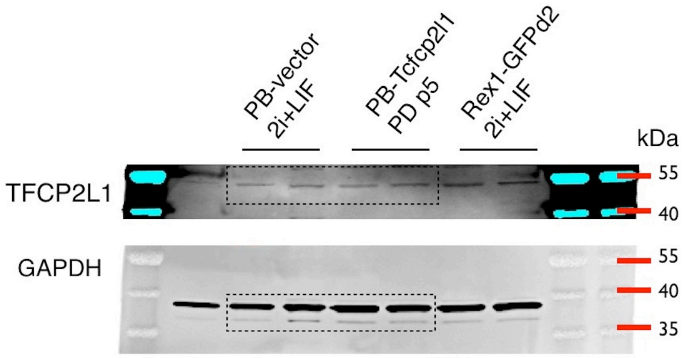

Un-cropped Western blot of Rex1GFPd2 cells un-transfected or transfected with an empty vector (PB-vector) cultured in 2i+LIF, and PB-Tfcp2l1 cells cultured in PD for 5 passages. For each line 2 biological replicates were loaded. GAPDH served as a loading control. Blots were imaged on a LI-COR Odyssey Imager.
